# Design, synthesis, *in vitro*, and *in silico* studies of novel isatin-hybrid hydrazones as potential triple-negative breast cancer agents†

**DOI:** 10.1039/d4ra07650h

**Published:** 2025-01-13

**Authors:** Iqra Munir, Zahra Batool, Faizullah Khan, Javid Hussain, Ajmal Khan, Suraj N. Mali, Vishnu Vasanthi Radhakrishnan, Bijo Mathew, Tahani Mazyad Almutairi, Ahmed Al-Harrasi, Muhammad Safwan Akram, Zahid Shafiq

**Affiliations:** a Institute of Chemical Sciences, Bahauddin Zakariya University Multan-60800 Pakistan zahidshafiq@bzu.edu.pk; b Department of Pharmacy, Abdul Wali Khan University Mardan KPK Pakistan aharrasi@unizwa.edu.om; c Department of Biological Sciences and Chemistry, University of Nizwa Oman; d Natural and Medical Sciences Research Centre, University of Nizwa P. O. Box 33, PC 616, Birkat Al Mauz Nizwa Sultanate of Oman; e School of Pharmacy, DY Patil Deemed To Be University Navi Mumbai India; f Dept. Pharmaceutical Chemistry, Amrita School of Pharmacy, Amrita Vishwa Vidyapeetham Cochin India; g Department of Chemistry, College of Science, King Saud University Riyadh 11451 Saudi Arabia; h School of Science & Health, Teesside University Middlesbrough TS1 3BA UK; i National Horizons Centre, Teesside University 38 John Dixon Ln Darlington DL1 1HG UK; j Department of Chemical and Biological Engineering, College of Engineering, Korea University 145 Anam-ro, Seongbuk-gu Seoul 02841 Republic of Korea

## Abstract

Recent advances in cancer therapy have been made possible by monoclonal antibodies, domain antibodies, antibody drug conjugates, *etc.* The most impact has come from controlling cell cycle checkpoints through checkpoint inhibitors. This manuscript explores the potential of a series of novel *N*-benzyl isatin based hydrazones (5–25), which were synthesized and evaluated as anti-breast cancer agents. The synthesized hydrazones of *N*-benzyl isatin were screened *in vitro* against two cell lines, the MDA-MB-231 breast cancer cell line and the MCF-10A breast epithelial cell line. The results indicated that all compounds showed great potential against the triple-negative MDA-MB-231 breast cancer cell line. Compound 23 with nitro substitution at the 4th position of the phenyl ring exhibited significant antiproliferative potential for the MDA-MB-231 with an IC_50_ value of 15.8 ± 0.6 μM. Molecular dynamics and molecular docking simulations were performed to get a deeper understanding of the interactions between the synthesized compounds and cancer cells.

## Abstract 2

Recent advances in cancer therapy have been made possible by monoclonal antibodies, domain antibodies, antibody drug conjugates, *etc.*

## Graphical abstract

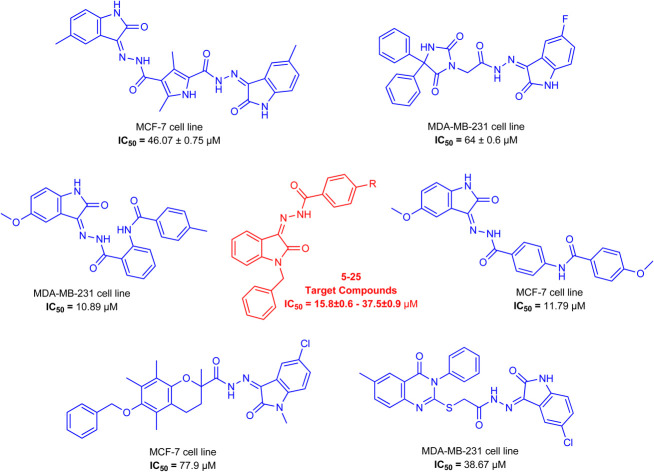

## 1 Introduction

One of the leading causes of mortality worldwide is cancer, which is seen as a significant barrier to increasing life expectancy.^1–6^ Tumor cells' ability to adapt to hypoxia and acidosis is one of the primary mechanisms supporting the growth and spread of malignancies. Many solid tumors have an acidic microenvironment because of the excess lactic acid produced by cancer cells due to anaerobic glucose metabolism and inadequate vascular clearance. The hypoxic tumor microenvironment accelerates tumor growth, decreases patient survival rates, and makes a variety of malignancies more aggressive.^7,8^

Triple Negative Breast Cancer (TNBC) is one of the most aggressive forms of Breast Cancer (BC), which is associated with a very poor prognosis. It is a broad category of tumors with a variety of biological, clinical, and morphological characteristics.^9,10^ BC was the most invasive and significant cause of death for women in 2020, accounting for 684 996 deaths and over 2.3 million new diagnoses.^11^ For BC therapy, in addition to chemotherapy and radiotherapy, new developments in chemotherapy alternatives have raised overall survival (OS) rates.^12,13^ Unfortunately, there are often significant drawbacks to the chemotherapy that is currently on the market, most notably their lack of specificity, which can be hazardous to the body and eventually result in the development of multidrug resistance.^14,15^ Furthermore, TNBC is still an incurable condition. Given these circumstances, taking advantage of prospective anti-BC chemotherapeutics is necessary. In order to address the issue various strategies have been adopted.

Hydrazones are a class of synthetic organic molecules with HC=N–NH-bond and are now of significant technical and economic relevance for various pharmacological advantages including antibacterial, anticancer,^16–19^ antiprotozoal, anti-inflammatory,^20^ antioxidant, cardioprotective *etc.*^21,22^ Products having distinct biological characteristics can be formed by combining hydrazones with various functional groups.^23^ Hydrazones are viable for synthesizing new moieties because of the functional nitrogen electron pair. They can extensively be used as adaptable ligands in coordination chemistry.^24^

**Fig. 1:**
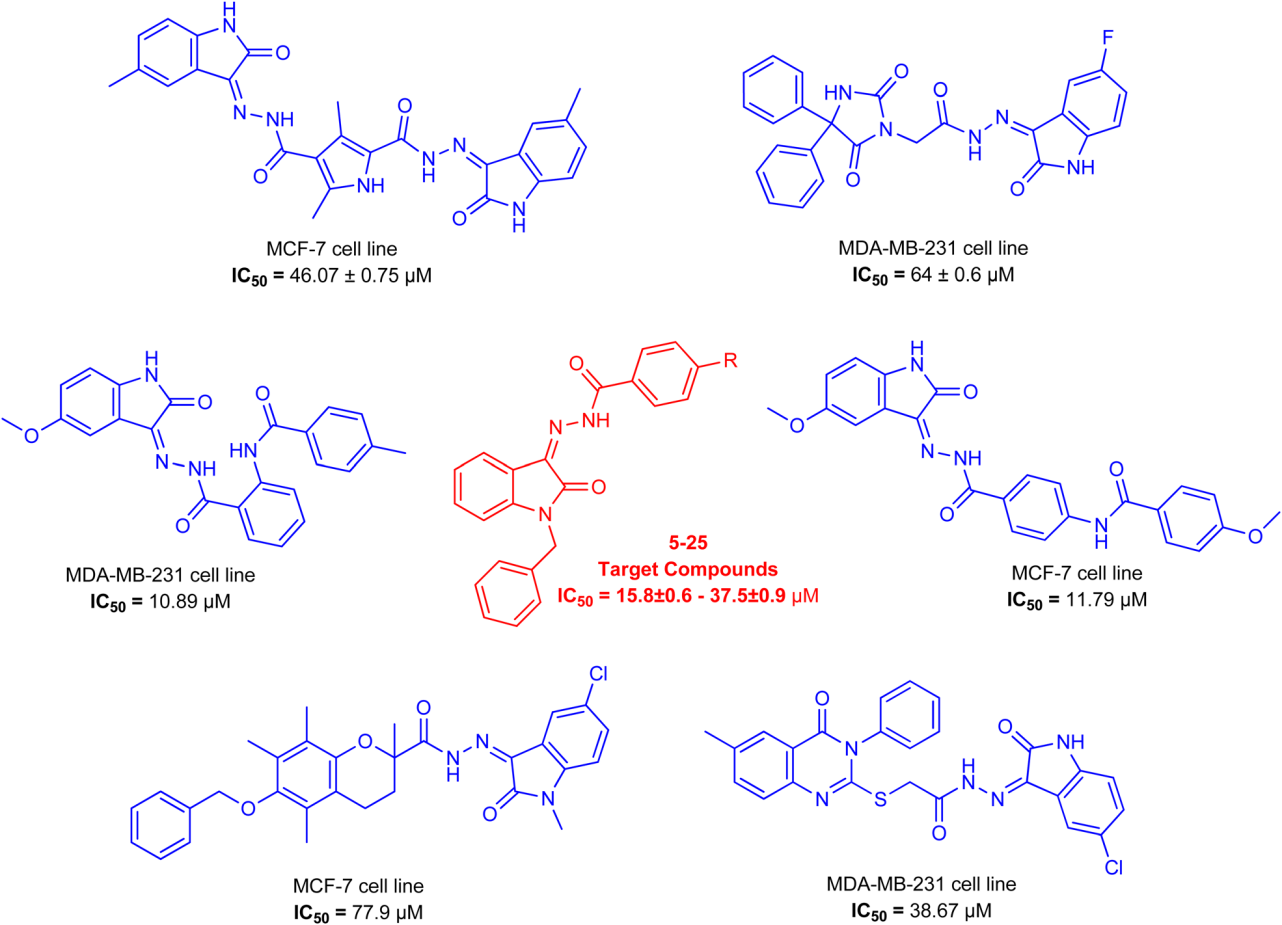
Structures of reported isatin based hydrazones.

As a unique class in drug design and discovery, isatin (1*H*-indole-2,3-dione) is one of the most advantageous scaffolds of heterocyclic systems, with a diversity of remarkable biological activities, such as antibacterial,^25^ anticonvulsant, and mostly anticancer properties.^26–29^ Isatin is a heterocycle scaffold that is the foundation of many drugs. Many isatin based derivatives have obtained significant importance in medicinal chemistry such as anti-cancer, anti-tumor and anti-inflammatory. Isatin targets multiple cellular mechanism, including angiogenesis, cell cycle, checkpoint pathways, and multiple kinases. As a result, the isatin nucleus has been widely employed in the synthesis of a variety of potent oxindole-based small molecules with anticancer properties that target various cellular and enzymatic targets, including the initiation of apoptosis in different human cancer cell lines^30,31^ and the inhibition of the carbonic anhydrase IX isoform^32^ linked to cancer. In several ground-breaking compounds and research, isatin has demonstrated efficacious potential against breast cancer cells. Some of the previously reported isatin-based hydrazones along with their IC_50_ values are displayed in Fig. 1.^19,33–37^

To obtain medicines through a unique mode of action or generate a synergistic impact, blending two or more pharmacophoric moieties in a single molecule is a primary strategy for developing drugs.^38,39^ In light of the previous findings and in continuation of our earlier research on discovering new antiproliferative activities,^40–42^ hybrid compounds with a hydrazone moiety and an *N*-benzyl isatin moiety were synthesized employing the condensation reaction in the hope of generating new, potent anticancer molecules. The synthesized compounds were further explored through molecular docking to find their binding interactions with the cancer cells (Table 1).

**Table 1:** Summary of clinical trials and approval for isatin derivatives according to ClinicalTrials.gov database^43^

Name of drug	Year	FDA approved clinical indications
Sunitinib	2006	Gastrointestinal stromal tumors and advanced renal cell carcinoma
	2011	Pancreatic cancer
	2017	Adjuvant agent for recurrent renal carcinoma
	2019	Phase 2 in metastatic pancreatic neuroendocrine tumor
	2020	Metastatic renal cell carcinoma
Toceranib	2009	Canine mast cell tumor
Nintedanib	2018	Phase 3 completed for refractory metastatic colorectal cancer
	2018	Phase 3 completed for combination with paclitaxel and carboplatin for use in ovarian cancer (first line therapy)
	2018	Phase 3 completed for combination with docetaxel for use in non-small cell lung cancer
	2019	Phase 1 completed for combination with letrozole for breast cancer in postmenopausal women
	2019	Phase 2 completed for recurrent or metastatic breast cancer
	2019	Phase 2 terminated for metastatic HER2-negative inflammatory breast cancer
	2019	Phase 2 completed for advanced ovarian cancer
Orantinib	2011	Phase 1/2 completed for use in advanced hepatocellular carcinoma
	2017	Phase 3 in hepatocellular carcinoma
Semaxinib	2003	Phase 2 completed for use in persistent and recurrent cervical cancer
	2004	Phase 3 completed for use as combination with 5-fluorouracil, leucovorin, and irinotecan in metastatic colorectal cancer
	2009	Phase 2 completed for use in advanced/recurrent head and neck cancer

## 2 Results and discussion

### 2.1. Chemistry

**Scheme 1:**
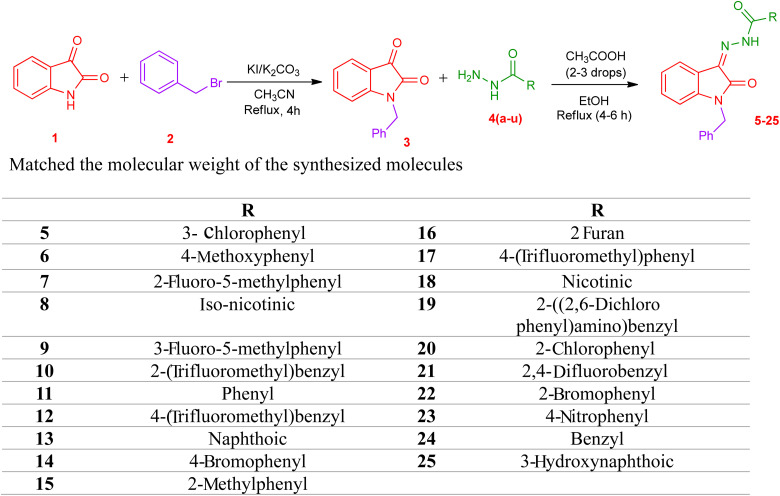
Synthesis of hydrazone derivatives (5–25).

The synthetic procedure of compounds 5–25 is outlined in Scheme 1. Synthesis of *N* benzyl isatin-based hydrazones was done in two steps. 1-Benzylindoline-2,3-dione (3) was synthesized from commercially available indoline-2,3-dione (1) in the first step. *N*-Benzylation of isatin was carried out *via* a reaction of isatin with benzyl bromide in the solvent acetonitrile in the presence of potassium carbonate and potassium iodide to yield 3.

In the second step, hydrazone was synthesized by refluxing the equimolar ratio of *N*-benzyl isatin and the respective hydrazide 4(a–u) in methanol, and acetic acid was used as a catalyst. The solid product obtained is filtered to yield *N* benzyl isatin based hydrazones 5–25 (Scheme 1).

The structures of the synthesized compounds 5–25 were confirmed by FTIR, ^1^H NMR, and ^13^C NMR spectroscopy. In the FTIR spectra, the absorption of the carbonyl group in hydrazide moiety and NH bands in CONH were observed in the 1651 cm^−1^ and 3203–2892 cm^−1^ regions, respectively.

The ^1^H NMR spectra showed a singlet peak at 11.58–13.94 ppm which can be attributed to the NH group of carbohydrazide. In compounds 19–25, another peak is observed in the range of 11.32–14.64 ppm which can be attributed to iminol group. This indicates the possibility of the formation of tautomer in some of the synthesized compounds. The CH_2_ of the benzyl moiety attached to the N position of the isatin appeared as a singlet in the region of 4.96–5.04 ppm. All other aromatic protons can be seen in the range of 7–8 ppm.

In ^13^C NMR spectroscopy data of compound 19–25, peaks displayed an increase in the number of carbon atoms compared to the desired synthesized compound. There were 3 peaks (151 MHz) shown in the carbonyl region 160–210 ppm which shows there was a phenomenon of tautomerism.

The molecular ion peaks in the HRMS spectra were represented as [M + H]^+^, and they perfectly matched the molecular weight of the synthesized molecules.

### 2.2. Biological activity

Breast cancer cells MDA-MB-231 were used to investigate the synthesized compounds' anticancer properties. Because these cancer cells lack estrogen receptors (ER), progesterone receptors (PR), and human epidermal growth factor receptor 2 (HER2), they are also known as triple-negative breast cancer cells (TNBC).^44^ These receptors are generally utilized as target proteins in cancer treatment and are often overexpressed in cancer cells. MDA-MB-231 cells, on the other hand, exhibit minimal or no receptor expression and are an aggressive, non-targetable form of cancer.^45^ Additionally, the cells exhibit resistance to routinely used chemotherapeutic medicines. One of the most used medications for TNBC treatment, doxorubicin, exhibits substantial toxicity but also has serious adverse effects, including cardiotoxicity.^46^ At the same time human normal breast epithelial cell line MCF-10A was kept as a control in the experiment to check out whether the cytotoxic effects of the compounds were selective for malignant cells in comparison to non-malignant cells. Variable concentrations (6.5 μM, 12.5 μM, 25 μM, and 50 μM) of the synthesized compounds 5–25 were used to investigate the prohibition of growth in the human breast cancer cell line MDA-MB-231 and the non-tumorigenic MCF-10. The MTT [3-(4,5-dimethylthiazol-2-yl)-2,5-diphenyltetrazolium bromide] assay was used to determine the reduction in cancer cell viability induced by cytotoxic agents. For both cell lines, the IC_50_ values, percent inhibition, and viability of compounds 5–25 are displayed in Table 2. IBM SPSS Statistics 26 software was used for the analysis of dose–response and computation of IC_50_ values. Findings of the MTT assay demonstrate that all compounds displayed potent activity against MDA-MB-231 cells. Among all, compound 23 showed more potential toward the MDA-MB-231 cell line possessing the IC_50_ value of 15.8 ± 0.6 μM. For MCF-10A cell lines, these results showed that the cells were less susceptible to the actions of the synthesized compounds, particularly compound 23, which appeared to cause more cell death in breast cancer cells.

**Table 2:** The percent viability and inhibition of synthetic derivatives on breast cancer cell line MDA-MB-231 and normal cell line MCF-10

	Structures	Breast cancer cell line MDA-MB-231		Normal cell line MCF-10	
		% viability	IC_50_ values	% viability	IC_50_ values
5	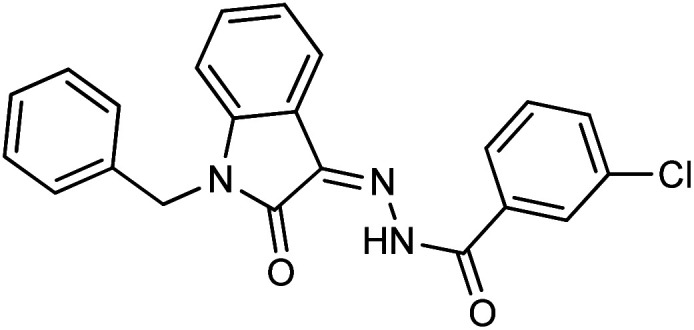	27.11	26.4 ± 0.8	80.14	>50
6	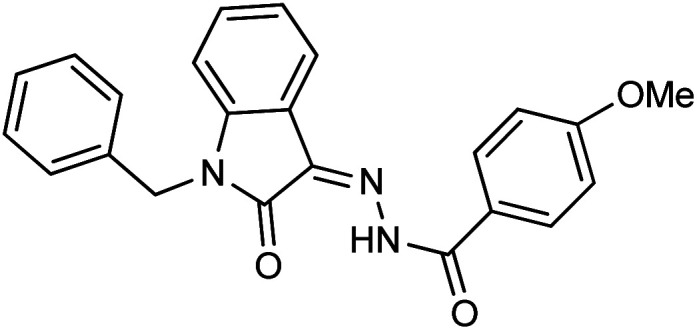	26.13	25.8 ± 0.4	81.13	>50
7	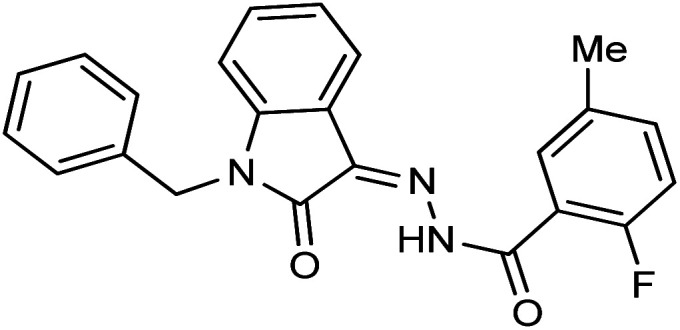	30.01	33.6 ± 0.5	79.34	>50
8	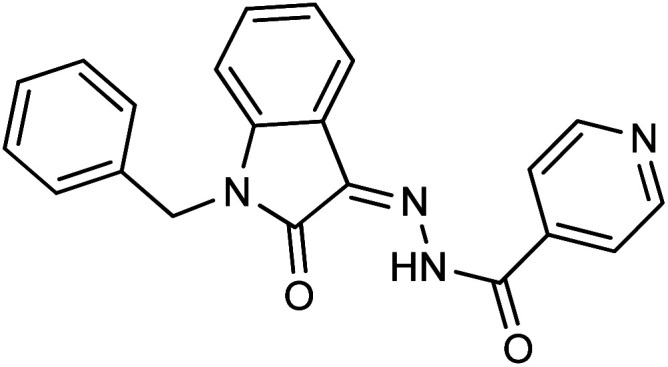	19.13	16.8 ± 0.9	78.14	>50
9	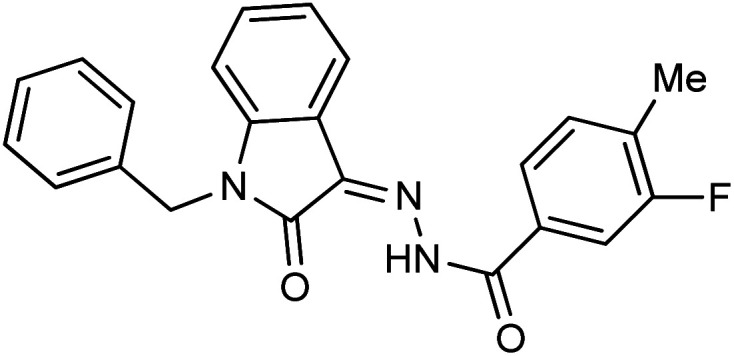	25.61	27.4 ± 0.5	76.19	>50
10	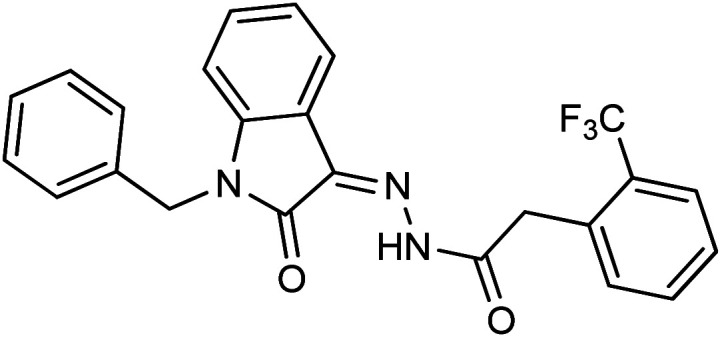	29.12	25.4 ± 0.8	84.89	>50
11	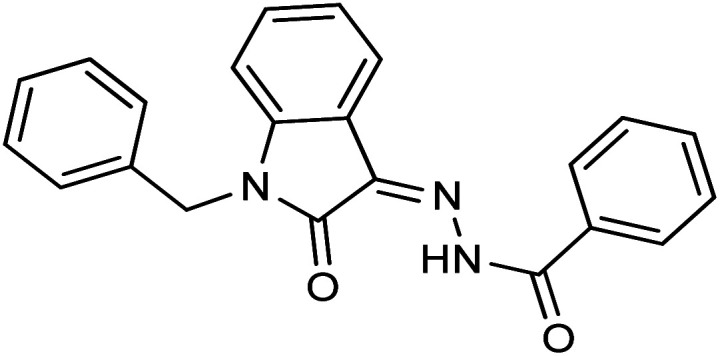	25.41	28.4 ± 0.5	81.99	>50
12	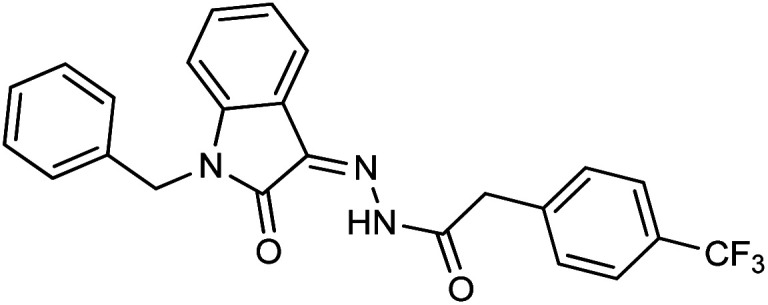	36.52	31.2 ± 0.9	80.44	>50
13	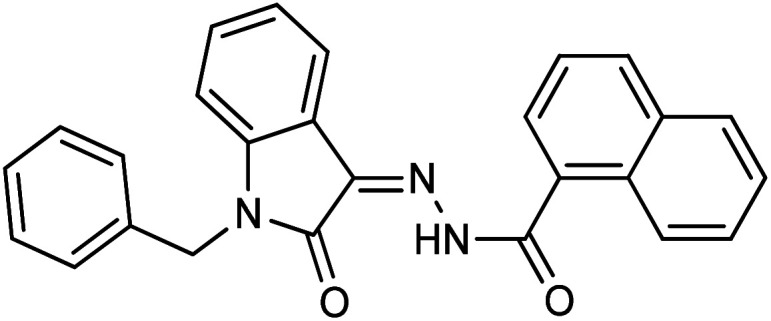	29.01	28.6 ± 0.5	79.09	>50
14	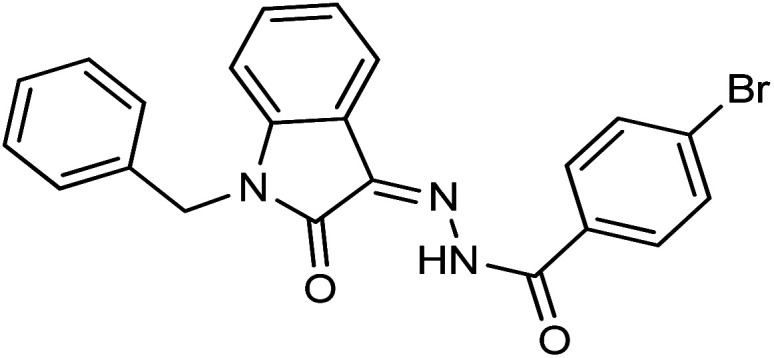	42.11	36.8 ± 0.7	85.37	>50
15	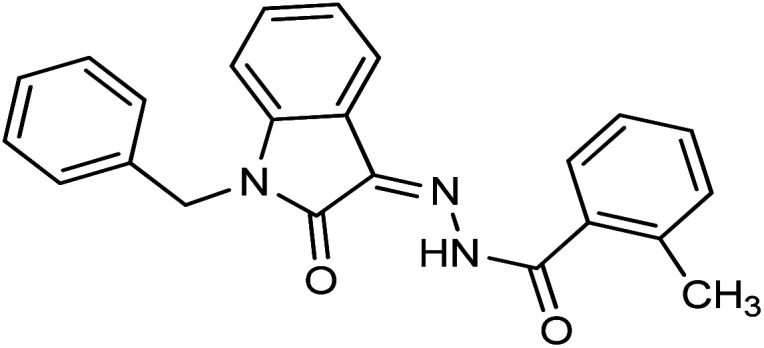	23.77	24.4 ± 0.6	84.34	>50
16	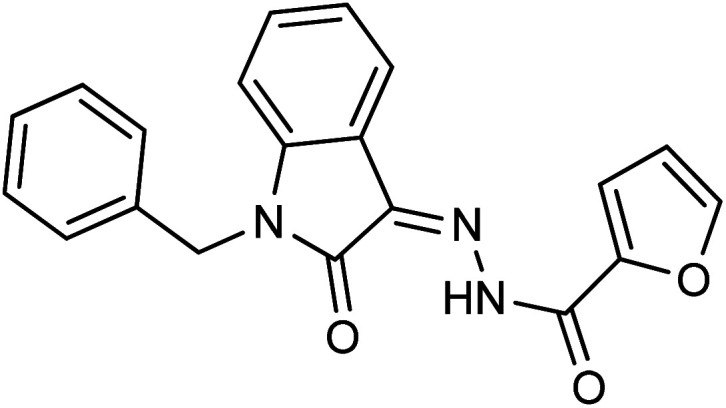	29.40	29.2 ± 0.7	79.91	>50
17	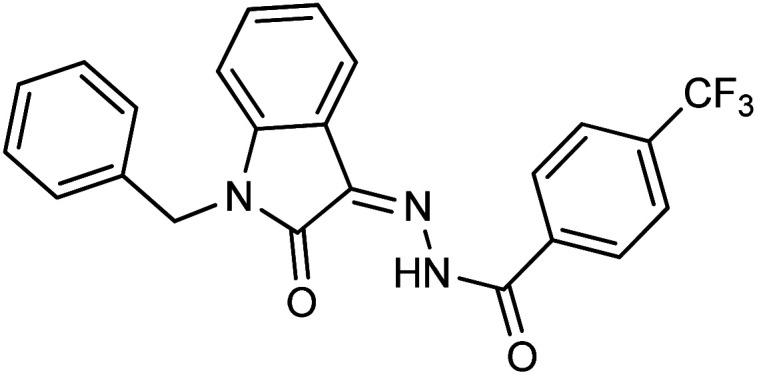	38.09	37.5 ± 0.9	77.56	>50
18	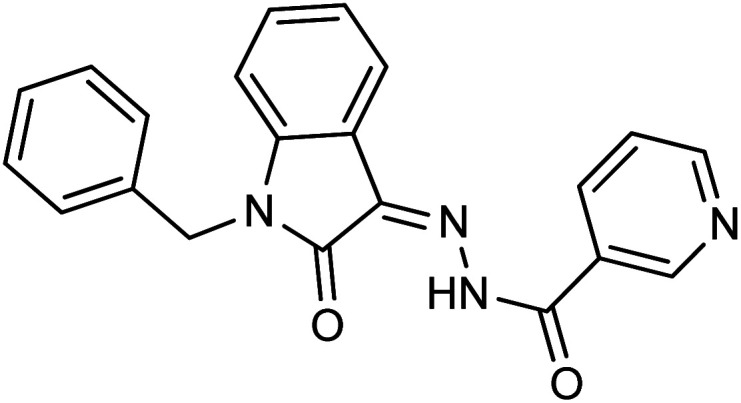	41.19	34.6 ± 0.4	85.13	>50
19	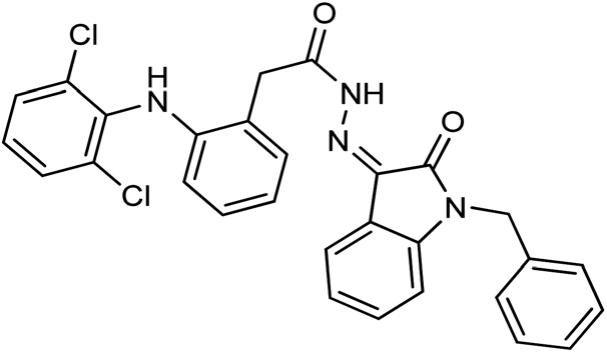	37.23	29.2 ± 0.4	80.33	>50
20	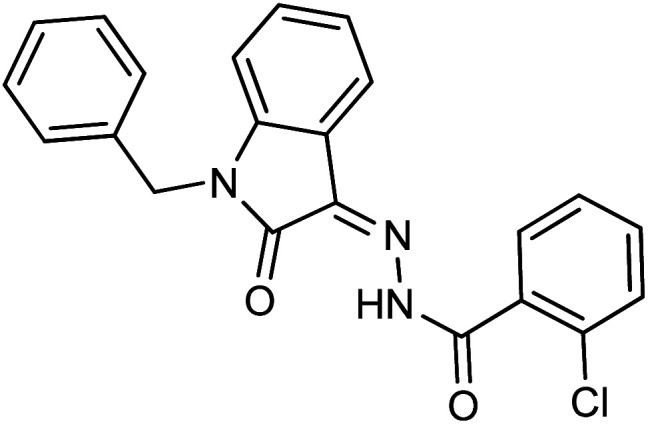	34.56	28.8 ± 0.6	77.56	>50
21	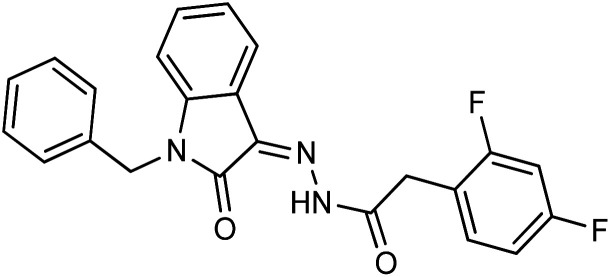	22.46	22.2 ± 0.7	85.71	>50
22	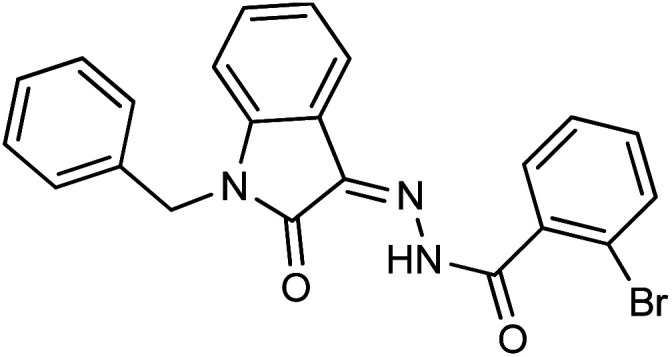	21.28	26.1 ± 0.7	78.41	>50
23	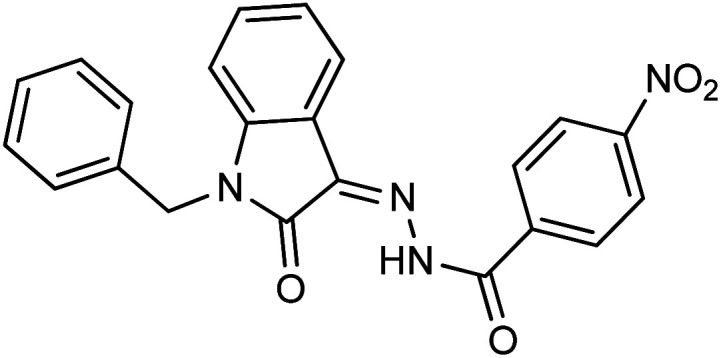	17.37	15.8 ± 0.6	75.23	>50
24	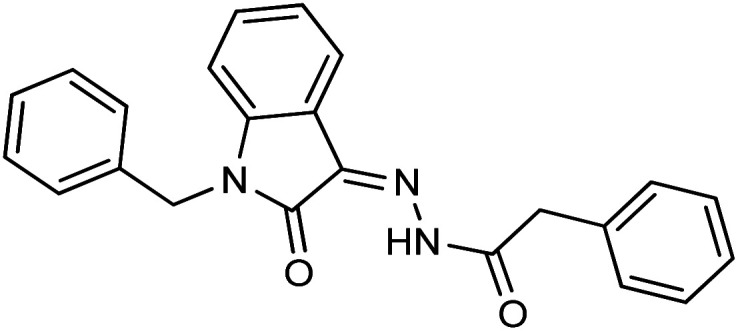	29.54	24.5 ± 0.8	78.47	>50
25	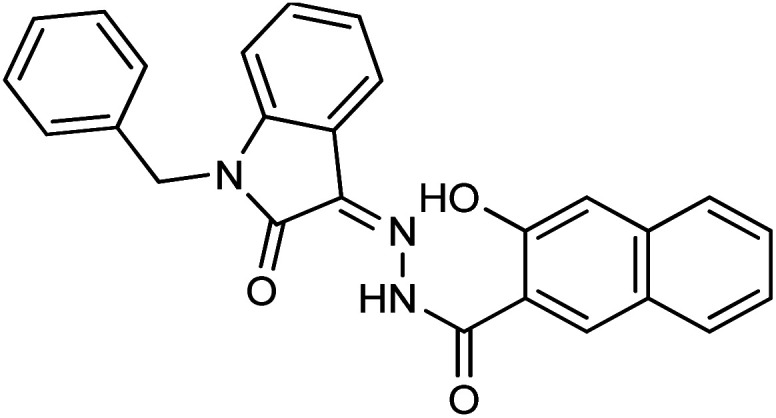	31.67	30.6 ± 0.4	76.29	>50

Data from this study showed that the triple negative MDA-MB-231 cells, which have an aggressive phenotype, reacted more favorably to most of the compounds and given greater cytotoxicity. The decrease in cytotoxicity observed when non-tumorigenic MCF-10A cells were shown exposure to compounds 5–25 suggests that these novel compounds offer great treatment/therapy for patients having non-responsive breast cancer.

#### 2.2.1 Structure–activity relationship

The structure–activity relationship is largely determined by the R group attached to the hydrazide moiety. In the current work, we used various aromatic rings such as phenyl, benzyl, naphthyl, and pyridyl, *etc.* at different positions and the nature of the attached substituents.

Comparing the inhibition potential of various rings attached to the hydrazide moiety, compound 8 with pyridyl ring linked with the *para* position to the hydrazide moiety displayed outstanding inhibition potential with IC_50_ value of 16.8 ± 0.9 μM and is the second most active derivative of the series followed by compound 24 with benzyl ring with an IC_50_ value of 24.5 ± 0.8 μM. Compound 11 containing a phenyl ring displayed moderate inhibition potential with an IC_50_ value of 28.4 ± 0.5 μM, and compound 13 with a naphthyl ring showed almost similar inhibition potential as compound 11. Compound 16 with furan ring displayed moderate potency with an IC_50_ value of 29.2 ± 0.7 μM. The following observation revealed that the size of the ring is not a significant factor in determining the inhibition potency. Compound 18 with a nicotinic ring displayed much lower inhibition potential in comparison with compound 8 with an iso-nicotinic ring.

It is noteworthy that compound 23 with nitro substitution attached to the *para* position of the phenyl ring is the top contender of the entire library of compounds displaying an IC_50_ value of 15.8 ± 0.6 μM. It shows the importance of the electron-withdrawing group in inhibiting cancer cells. While compound 6 having methoxy substitution at the *para* position of the phenyl ring showed moderate inhibition with an IC_50_ value of 25.8 ± 0.4 μM.

On comparing the potency of halogen-containing derivatives, compound 21 with the fluoro group at the 2 and 4 positions of the benzyl ring displayed marvelous inhibition potential with an IC_50_ value of 22.2 ± 0.7 μM and is the third most potent member of the series followed by compound 10 with trifluoro methyl group at the *ortho* position of the phenyl ring with an IC_50_ value of 25.4 ± 0.8 μM. Interestingly, compounds 10 and 17 with trifluoromethyl group at the 2 and 4 positions of the phenyl ring, respectively, displayed a huge difference in the inhibition potential values. Compound 17 showed a steep decline in the inhibition potential with an IC_50_ value of 37.5 ± 0.9 μM and is the least active member of the series. Likewise compounds 22 and 14 with bromo substitution at 2 and 4 positions of the phenyl ring displayed the same phenomenon. Compound 14 with the bromo group at the *para* position of the phenyl ring displayed much less potential than compound 22 with the bromo group at the *ortho* position. It revealed the importance of substitution at the *ortho* position, which is favorable for inhibiting breast cancer cells. Compounds 5 and 20 with chloro substitution at the *meta* and *ortho* positions of the phenyl ring respectively displayed almost similar inhibition potential with IC_50_ values of 26.4 ± 0.8 μM and 28.8 ± 0.6 μM respectively. Compound 19 with chloro substitutions at the 2 and 6 positions of the phenyl ring showed a further decrease in inhibition potential. A steep decline in the inhibition potential can be seen in the comparison of compound 21 with difluoro substitutions with compound 19 with dichloro substitutions which reveals the fact that high electronegativity favors the inhibition of cancer cells.

**Fig. 2:**
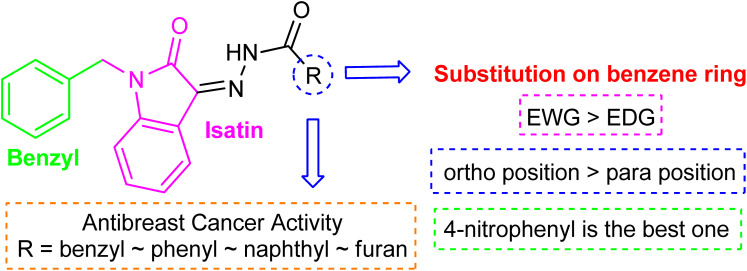
Pictorial representation of structure–activity relationship.

Compound 15 with methyl substitution at the *ortho* position of the phenyl ring showed significant inhibition potential with IC_50_ values of 24.4 ± 0.6 μM. Compound 9 with methyl substation at the *para* and fluoro at the *meta* position of the phenyl ring showed a decrease in the potency with an IC_50_ value of 27.4 ± 0.5 μM. While in compound 7 with 2 fluoro and 5 methyl substitutions at the phenyl ring, inhibition potential is lowered significantly which can be due to the steric hindrance on interaction with the cancer cells (Fig. 2).

### 2.3. Molecular docking and binding free energy calculation

**Fig. 3:**
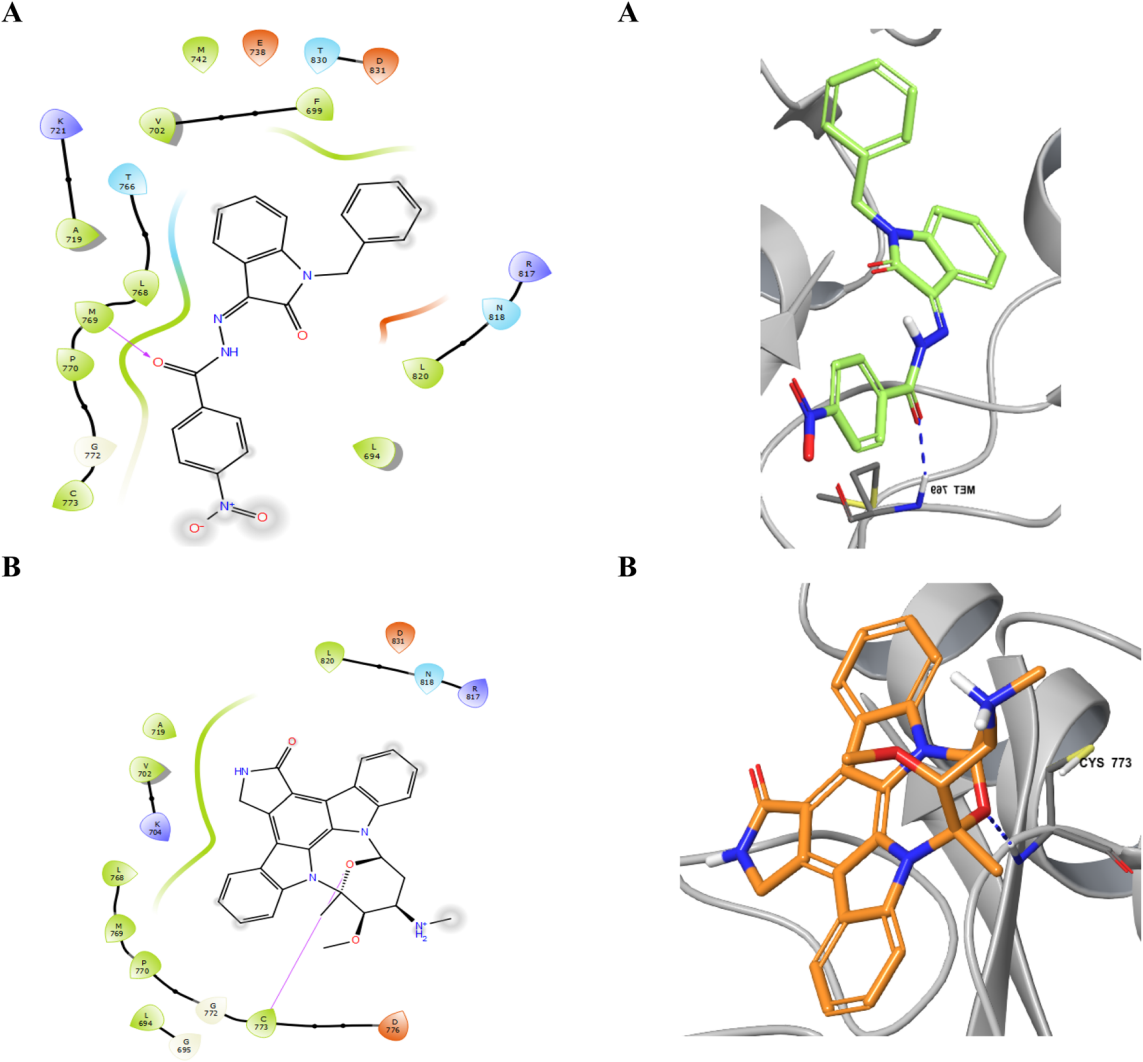
The 2D and 3D interaction of most potent ligand 23 (A) and control drug Staurosporine (B).

**Fig. 4:**
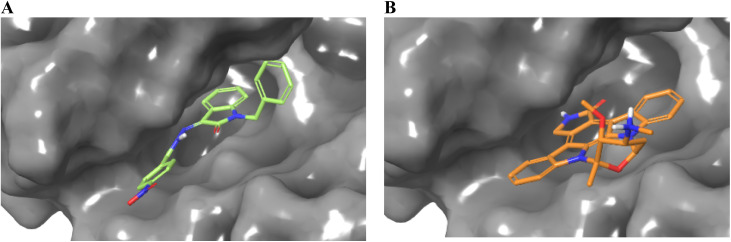
Occupancy of ligands 23 (A) and Staurosporine (B) in the binding cavity of EGFR kinase domain target protein.

Considering the structural features and literature background, we performed the docking studies using a Glide module from Schrodinger, Inc., New York, USA (2024). The grid for the docking study was generated at the site where erlotinib was bound as the co-crystal ligand. The active site of the EGFR kinase domain is composed of 25 residues, including polar residues T766, Q767, and T830, and the charged residues K721 (positive charge) and D776 (negatively charged) while the other residues were hydrophobic. The docking score and interacting residues of our synthesized compounds as shown in Table 3 disclosed that the most biologically potent ligand 23 is having docking score and binding free energy of −7.561 kcal mol^−1^ and −55.35 kcal mol^−1^ respectively than the control ligand Staurosporine has −4.572 and −32.53 kcal mol^−1^ respectively. The 23 ligand displayed a better occupancy in the binding site as depicted in Fig. 3 and the carbonyl oxygen interacts with M769 through hydrogen bonding with a distance of 1.92 Å, while the control drug Staurosporine was able to occupy the cavity partially only and interacts with C773 by hydrogen bonding with a distance of 2.09 Å as shown in Fig. 4. Other compounds were also showing interactions in the active site while compounds 5, 21, 9, and 24 were only showing hydrophobic interactions in the active site. The ligand 23 had better flexibility due to the rotatable bonds present in comparison to Staurosporine which has a very restricted planar geometry due to its complex indolocarbazole structure. Additionally, the co-crystal ligand in the active site AQ4 showed hydrogen bonding with M769 only (2.03 Å distance), which is the key residue involved in the activity, and the same hydrogen bond was maintained by our compound 23 while Staurosporine lacked this. As shown in Table 3 compounds 20 (K721 and F699), 25 (C773), 15 (K721), 17 (K721, F699, and D831), 13 (K721 and D831), and 16 (K721, F699, and D831) did not have the key hydrogen bond interaction with M769 while compound 19 has an additional bond between chlorine atom in the dichlorobenzene ring with K721 residue while maintaining the hydrogen bond with M769. The data clearly shows that designed compounds particularly 23 have better biological activity towards the EGFR kinase domain than the control drug Staurosporine.

**Table 3:** XP docking score of synthesized compounds and binding free energy calculated (MMGBSA)

Compound	Docking score (kcal mol^−1^)	MMGBSA score (kcal mol^−1^)	Key residues interaction and type	
7	−6.085	−55.869	M769	Hydrogen bond
14	−5.799	−53.856	M769	Hydrogen bond
6	−5.668	−56.369	M769	Hydrogen bond
25	−5.631	−45.552	C773	Hydrogen bond
23	−7.561	−55.352	M769	Hydrogen bond
5	−5.471	−49.140		
20	−5.363	−56.829	K721	Halogen bond
			F699	Pi–Pi stacking
9	−5.352	−59.717		
15	−5.276	−46.959	K721	Hydrogen bond
21	−5.229	−47.455		
18	−5.13	−56.676	M769	Hydrogen bond
22	−5.033	−54.494	M769	Hydrogen bond
17	−4.875	−45.872	M721	Halogen bond
			F699	Pi–Pi stacking
			D831	Hydrogen bond
13	−4.87	−43.879	K721	Hydrogen bond
			D831	Hydrogen bond
24	−4.78	−46.526		
16	−4.668	−57.057	K721	Hydrogen bond
			F699	Pi–Pi stacking
			D831	Hydrogen bond
10	−4.651	−47.828	M769	Hydrogen bond
8	−4.19	−55.825	M769	Hydrogen bond
12	−4.131	−47.694	M769	Hydrogen bond
11	−4.028	−56.099	M769	Hydrogen bond
19	−3.796	−43.801	K721	Halogen bond
			M769	Hydrogen bond
Staurosporine	−4.572	−32.533	C773	Hydrogen bond

### 2.4. Molecular dynamics simulation

**Fig. 5:**
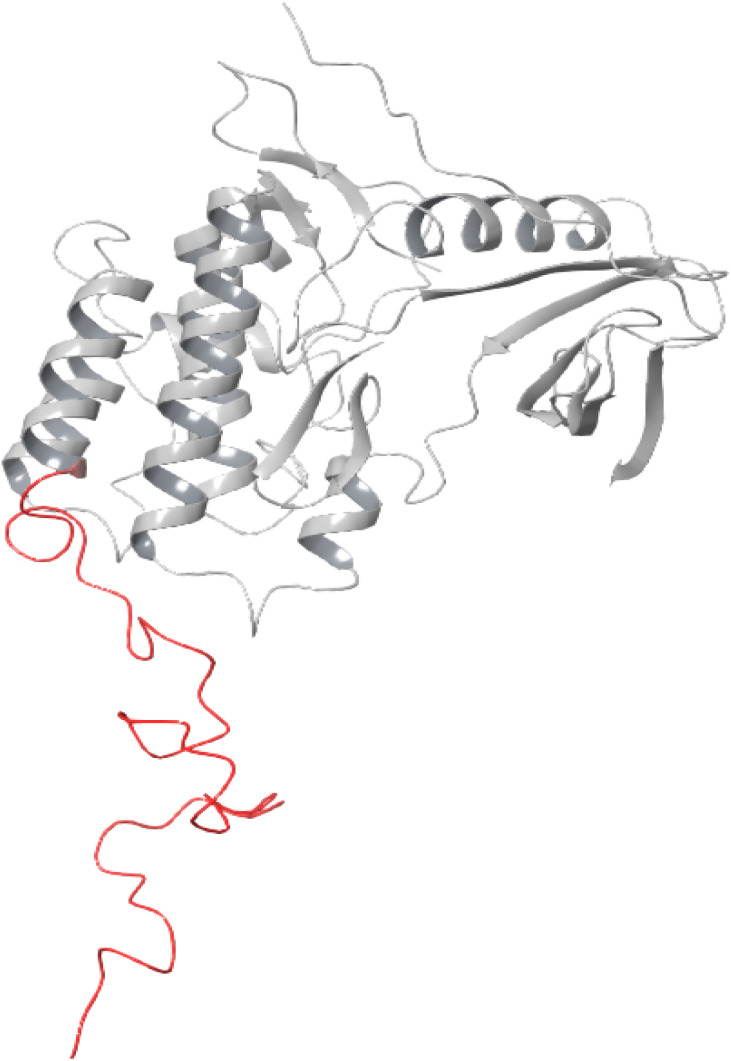
Epidermal growth factor receptor (EGFR) kinase domain (PDB ID 1M17). A random coil with 46 amino acids is represented in red color.

**Fig. 6:**
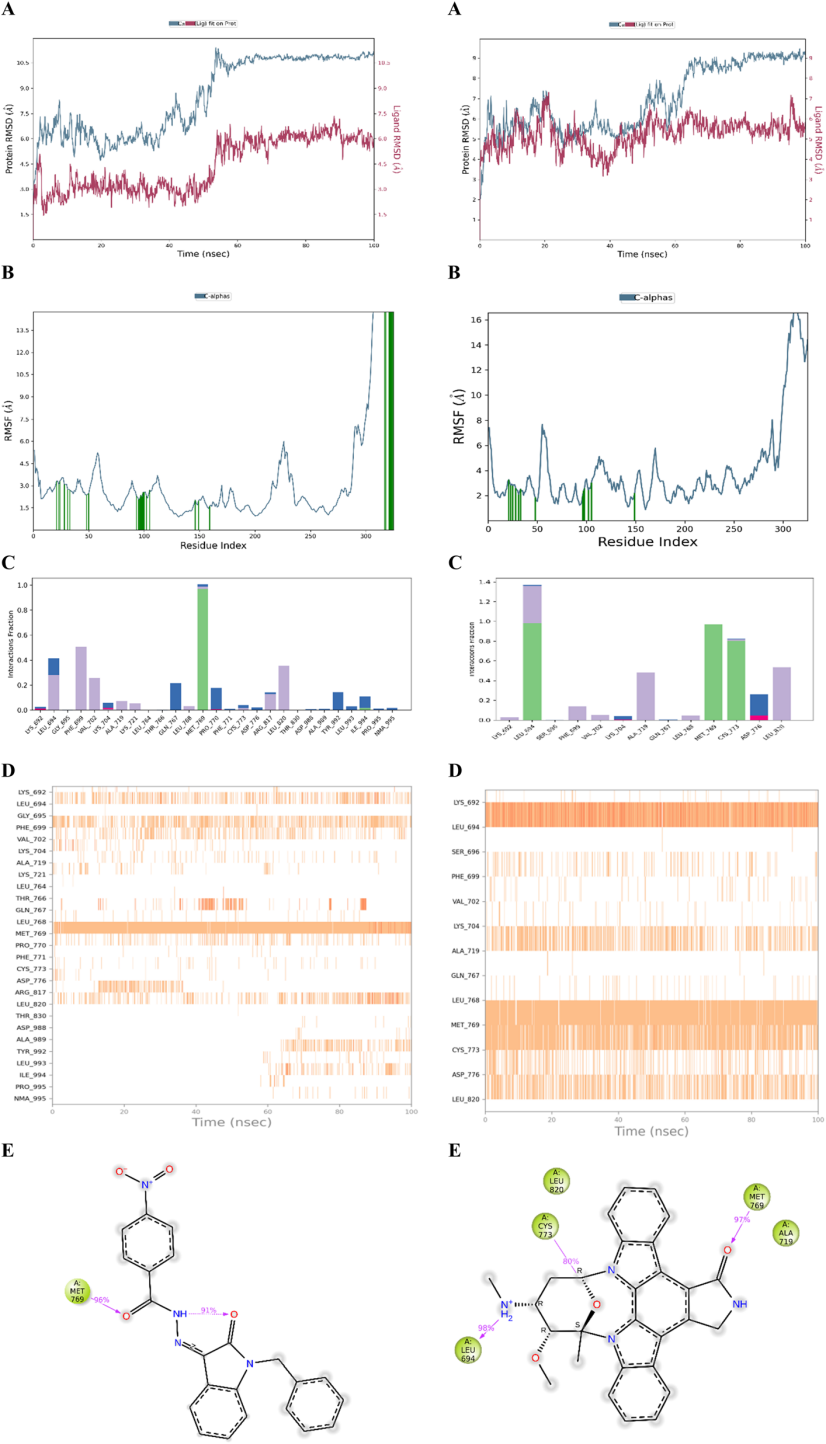
(A) RMSD plot of 23 and Staurosporine complex. (B) RMSF plot of 23 and Staurosporine complex. (C) Interaction histogram of 23 and Staurosporine complex. (D) Heat map profile of interactions 100 ns simulation period of 23 and Staurosporine complex. (E) Ligand protein contacts for more than 30% during 100 ns simulation of 23 and Staurosporine complex.

The stability of the docked complex of the most potent ligand 23 was evaluated using a molecular dynamics simulation study (MDS). The MDS analysis was performed for 100 ns time period comprising 55 414 total atoms including water molecules. In the simulation study, we evaluated the stability of the protein–ligand complex by considering the average displacement of atoms that occurred during the simulation using the RMSD variable (Root Mean Square Deviation).^47–49^ The examination of the MDS study disclosed that the EGFR kinase domain tertiary structure has several loops and random coils. The large portion in the structure with 46 amino acids ranging from R949 to P995 was a random coil as depicted in Fig. 5 which can have huge flexibility during the simulation. Considering these structural features, we evaluated the stability of the docked complex by analyzing the trajectory frame by frame including variables like RMSD and RMSF. The initial analysis of RMSD for 23 and Staurosporine complexes disclosed that the 23 complex had large conformational changes leading to 10.5 Å RMSD at the end of simulation when compared with Staurosporine complex having 9 Å as the RMSD as depicted in Fig. 6A. Due to the structural features of the EGFR kinase domain, the stability of the complex cannot be determined using RMSD alone so next we evaluated the RMSF.^50^ The RMSF data showed that the 23 complex has lower fluctuations at the local regions while large conformational changes are observed at the last 46 amino acid-containing regions while the Staurosporine complex has more local fluctuations but less conformational changes at the last 46 amino acid-containing regions when compared as shown in Fig. 6B. Moreover, it was observed that the protein residues that interact with 23 ligand were in the 46 amino acid containing random coil region but not for the control drug Staurosporine. This observation leads us to an understanding that only 23 ligand influenced the random coil which leads to a higher RMSD than the Staurosporine complex. Further, we evaluated the complex by interaction profile and found that 23 maintained the hydrogen bond interaction with M769 for 96% of the simulation period while Staurosporine had an additional hydrogen bond interaction with M769, and L694 for 97% and 98% respectively while the interaction obtained during docking with C773 (hydrogen bonding) was also maintained for 80% throughout the simulation period as shown in Fig. 6C–E. These observations led us to evaluate the complexes by analyzing the trajectory frame by frame, which disclosed that the random coil region was moving towards ligand 23 and interacting when the ligand was situated in the binding site, but in the case of the Staurosporine bound complex this phenomenon was not observed. We then performed a binding free energy calculation to evaluate the stability of complexes. The binding free energy calculation revealed that the 23rd complex had higher energetically favored stability with an MMGBSA score of −79.12 kcal mol^−1^ while the Staurosporine complex was having −51.31 kcal mol^−1^. This collective information (both statistical and energy) from molecular dynamics study revealed that stable interactions and higher binding free energy for the complex 23 made it the most potent agent to inhibit the EGFR kinase domain target protein.

### 2.5. ADME/Tox

All the synthesized compounds were evaluated based on the ADME/Tox descriptors to evaluate the drug-like properties of the compounds. Nearly 50 significant parameters can be obtained from the QikProp module. The descriptors evaluate general parameters like Lipinski rule of five and other parameters like log *P*, BBB (blood–brain barrier) permeability, human oral absorption, HERG binding, and log *S* as shown in Table 4. These descriptors help us to evaluate the biological activity at the molecular level. The detailed analysis revealed that some molecules were not following the Lipinski rule while compounds 6, 8, 23, 11, 18, and 16 were the only compounds completely following the rule of five. The oral bioavailability has an important role in the biological action of drug molecules. All the molecules synthesized had less than 15 rotatable bonds and PSA (polar surface area) of less than 200 Å which makes them orally bioavailable. Moreover, the oral absorption parameter (Human Oral Absorption) indicates that compounds 20, 6, 8, 22, 23, 11, 24, 24, 15, and 16 are orally bioavailable. The Caco-2 permeability showed better intestinal absorption. The QP log *K*_hsa_ values between −1.5 and 1.5 describe the human serum albumin binding capacity of the compounds. The *Q* log *P* describes the solubility and QP log BB predicts the capability of blood–brain barrier permeation. The high log *P* and polar surface area indicates that compound 23 has better solubility and is difficult to cross BBB. The skin permeability (QP log *K*_p_) was calculated, which has a close correlation with lipophilicity and size of the molecule. The compound 23 had less negative value (−2.671) when compared indicating its low skin permeability. From the collected descriptors, compounds 6, 8, 23, 11, 18, and 16 have a better drug-like character but 23 shows better properties than other candidates and can act by effectively transporting, absorbing, and permeating the cell and producing drug-like activity towards EGFR kinase domain target.

**Table 4:** ADME properties of synthesized compounds

Molecule	MW	RB	DonorHB	AccptHB	QP log *P*_o/w_	QP log *S*	QP log HERG	QPPCaco	QP log BB	QPPMDCK	QP log *K*_p_	QP log *K*_hsa_	Human oral absorption	PSA	Rule of five
5	389.84	5	0	4	5.286	−6.308	−6.943	1543.685	−0.436	1945.282	−0.926	0.76	1	77.18	1
6	385.421	6	0	4.75	4.846	−5.697	−6.931	1542.882	−0.675	790.525	−0.86	0.61	3	85.299	0
7	387.412	5	0	4	5.362	−6.423	−6.824	1760.628	−0.464	1448.321	−0.932	0.852	1	76.549	1
8	356.383	5	0	5.5	3.731	−4.616	−6.798	832.987	−0.866	406.04	−1.405	0.194	3	90.204	0
9	387.412	5	0	4	5.314	−6.439	−6.827	1546.399	−0.524	1283.591	−1.055	0.848	1	77.164	1
10	437.42	6	0	4	6.068	−6.965	−7.038	2015.661	−0.339	3172.614	−0.583	0.998	1	76.116	1
11	355.395	5	0	4	4.779	−5.527	−7.005	1548.078	−0.588	793.403	−0.759	0.631	3	77.144	0
12	437.42	6	0	4	6.238	−7.633	−7.261	1527.625	−0.444	3436.586	−0.896	1.073	1	78.43	1
13	405.455	5	0	4	5.811	−6.655	−7.543	2015.439	−0.489	1055.207	−0.283	1.016	1	74.952	1
14	434.291	5	0	4	5.363	−6.416	−6.955	1546.107	−0.423	2100.027	−0.93	0.785	1	77.168	1
15	369.422	5	0	4	5.217	−5.987	−6.917	2207.435	−0.433	1164.266	−0.574	0.793	3	74.852	1
16	345.357	5	0	4.5	3.966	−4.445	−6.42	1345.597	−0.606	681.855	−1.052	0.29	3	87.33	0
17	423.394	5	0	4	5.801	−7.042	−6.991	1550.683	−0.338	3510.131	−0.988	0.92	1	77.14	1
18	356.383	5	0	5.5	3.732	−4.618	−6.799	831.638	−0.866	405.33	−1.407	0.195	3	90.281	0
19	529.424	8	1	4.5	7.561	−8.622	−7.85	2286.812	−0.42	3945.296	0.075	1.549	1	84.516	2
20	389.84	5	0	4	5.312	−6.073	−6.832	1968.518	−0.329	2199.414	−0.677	0.75	3	75.64	1
21	405.403	6	0	4	5.619	−6.491	−6.85	1785.711	−0.412	2466.866	−0.779	0.846	1	77.315	1
22	434.291	5	0	4	5.378	−6.153	−6.831	1984.779	−0.312	2386.779	−0.677	0.77	3	75.733	1
23	400.393	6	0	5	4.012	−5.531	−6.905	184.881	−1.676	79.79	−2.671	0.528	3	122.167	0
24	369.422	6	0	4	5.204	−5.986	−7.15	1611.924	−0.652	828.829	−0.649	0.775	3	77.023	1
25	421.454	6	0	3.75	5.848	−7.083	−7.69	843.972	−1.001	411.831	−0.976	1.133	1	94.076	1

## 3 Conclusion

In the current research, a series of novel *N*-benzyl isatin-based hydrazones 5–25 are reported and investigated for their potential to be a potent anti cancer agents for triple negative breast cancer. The synthesized compounds 5–25 were used against triple-negative MDA-MB-231 breast cancer cell line and human normal breast epithelial cell lines MCF-10A to check out the prohibition of cancer cell growth. All the compounds displayed potent anti-cancer activity against the MDA-MB-231 cell line. SAR studies showed that the prohibition of cancer cell lines became more effective using electron-withdrawing groups. The results of the current study encourage us to continue our anticancer activity screening with further structural modification in the future, as the newly studied hydrazone derivatives may act as potential drug candidate for breast cancer treatment.

## 4 Experimental

### 4.1. General

For the synthesis of isatin-based hydrazones, all the starting materials such as isatin were bought from Sigma Aldrich. Chemicals and solvents including ethanol, methanol, glacial acetic acid, petroleum ether, and ethyl acetate were bought from Merck and used as original. MDA-MB-231 cell lines were acquired from the American Type Culture Collection (ATCC) (Cat # CRM-HTB-26). Silica gel plates with aluminum backs were utilized to check the reaction progress and completion. A Bruker Ascend 600 MHz NMR spectrometer was used to get ^1^H NMR and ^13^C NMR spectra in deuterated solvents like DMSO-*d*_6_ at 25 °C (600 MHz for ^1^H and 151 MHz for ^13^C). NMR values were presented as chemical shifts (ppm), and coupling constants (*J*) were demonstrated in Hertz (Hz) to detail signal multiplicity. The mass spectrum was recorded on QTOF HRMS 6530 With 1260 HPLC.

### 4.2. Synthetic procedure for the synthesis of 1-benzylindoline-2,3-dione (3)

In a solution of isatin (6 mmol), acetonitrile (15 mL), KI (0.99 g, 1.2 mmol), and K_2_CO_3_ (0.99 g, 7.2 mmol) were added while stirring, then allowed to rest for five minutes, followed by drop by drop addition of benzyl chloride (1.29 g, 9 mmol). After refluxing for 4 h at 80 °C, the reaction mixture was cooled down to room temperature and filtered. The filtrate was dried under a vacuum in a rotary evaporator, and dissolved in ethyl acetate. Using a separatory funnel, the filtrate solution was extracted multiple times with hot water to obtain a clear solution indicative of the clearance of isatin. The product obtained was dried in the oven at 60 °C.

### 4.3. Synthetic procedure for the synthesis of *N*-benzyl isatin-based hydrazones 7(a–t)

For the preparation of hydrazones, equimolar quantities of *N*-benzyl isatin (0.4 mmol) and respective hydrazides (0.4 mmol) were dissolved in ethanol. A catalytic amount (2 to 3 drops) of acetic acid was added and refluxed for 4–6 hours. The progress of the reaction was tracked using TLC. Once the reaction was finished, it was allowed to cool to room temperature, and precipitates of the targeted hydrazones 5–25 were filtered. The precipitates were washed using ethanol and kept dried for further analysis.

#### 4.3.1 (*E*)-*N*′-(1-Benzyl-2-oxoindolin-3-ylidene)-3-chlorobenzohydrazide (5)

% Yield = 89, light yellow solid, m. p.: 179–181 °C, IR (KBr) cm^−1^ 3480, 3048, 1932, 1713, 1805, 1697, 1349, 1201, 893, 725, 531, *δ*_H_ (600 MHz, DMSO-*d*_6_) 13.83 (1H, s), 7.92 (1H, t, *J* = 1.9 Hz), 7.90–7.83 (1H, m), 7.77 (1H, dd, *J* = 7.9, 2.2 Hz), 7.66 (2H, t, *J* = 7.9 Hz), 7.42 (3H, d, *J* = 7.7 Hz), 7.35 (2H, t, *J* = 7.5 Hz), 7.29 (1H, t, *J* = 7.3 Hz), 7.16 (1H, t, *J* = 7.5 Hz), 7.08 (1H, d, *J* = 7.9 Hz), 5.03 (2H, s); ^13^C NMR (151 MHz, DMSO) *δ* 161.63, 143.29, 136.04, 134.55, 134.38, 133.17, 132.31, 131.67, 129.20, 128.15, 127.91, 127.82, 126.53, 123.96, 121.41, 119.67, 111.08, 43.08. TOF HRMS (*m*/*z*): [M + H]^+^, calcd: 390.1009, found: 390.1009.

#### 4.3.2 (*E*)-*N*′-(1-Benzyl-2-oxoindolin-3-ylidene)-4-methoxybenzohydrazide (6)

% Yield = 93, yellow solid, m. p.: 173–175 °C, IR (KBr) cm^−1^ 3436, 3043, 2356, 1923, 1658, 1343, 1156, 863, 764, *δ*_H_ (600 MHz, DMSO-*d*_6_) 13.84 (1H, s), 7.96–7.85 (2H, m), 7.70–7.64 (1H, m), 7.44–7.38 (3H, m), 7.35 (2H, t, *J* = 7.6 Hz), 7.32–7.26 (1H, m), 7.16 (3H, dd, *J* = 8.5, 6.4 Hz), 7.08 (1H, d, *J* = 7.9 Hz), 5.03 (2H, s), 3.86 (3H, s); ^13^C NMR (151 MHz, DMSO) *δ* 163.39, 161.75, 143.01, 136.13, 131.90, 129.21, 128.13, 127.93, 124.43, 123.87, 121.18, 119.89, 114.98, 110.98, 56.07, 43.07. TOF HRMS (*m*/*z*): [M + H]^+^, calcd: 386.1504, found: 386.1504.

#### 4.3.3 (*E*)-*N*′-(1-Benzyl-2-oxoindolin-3-ylidene)-2-fluoro-5-methylbenzohydrazide (7)

% Yield = 87, light brown solid, m. p.: 176–178 °C, IR (KBr) cm^−1^ 3493, 3048, 2917, 1952, 1819, 1691, 1604, 1497, 1182, 847, 652, *δ*_H_ (600 MHz, DMSO-*d*_6_) 13.78 (1H, s), 7.85 (1H, s), 7.75–7.58 (1H, m), 7.51 (1H, d, *J* = 6.7 Hz), 7.43–7.37 (3H, m), 7.34 (3H, q, *J* = 7.4 Hz), 7.32–7.25 (1H, m), 7.15 (1H, t, *J* = 7.5 Hz), 7.06 (1H, d, *J* = 8.0 Hz), 5.00 (2H, s), 2.37 (3H, s); ^13^C NMR (151 MHz, DMSO) *δ* 162.77, 161.13, 159.46, 157.80, 145.80, 144.91, 143.36, 136.33, 136.12, 135.27, 134.83, 132.22, 131.98, 129.23, 129.20, 128.83, 128.11, 128.06, 127.90, 127.80, 123.83, 123.72, 121.41, 119.82, 115.87, 110.95, 110.87, 43.07, 20.51. TOF HRMS (*m*/*z*): [M + H]^+^, calcd: 388.1416, found: 388.1416.

#### 4.3.4 (*E*)-*N*′-(1-Benzyl-2-oxoindolin-3-ylidene)isonicotinohydrazide (8)

% Yield = 88, yellow solid, m. p.: 189–191 °C, IR (KBr) cm^−1^ 3487, 3131, 3004, 2347, 1945, 1677, 1395, 1268, 712, 672, *δ*_H_ (600 MHz, DMSO-*d*_6_) 13.93 (1H, s), 8.88 (2H, d, *J* = 5.2 Hz), 7.82 (2H, d, *J* = 5.5 Hz), 7.67 (1H, s), 7.42 (3H, d, *J* = 7.3 Hz), 7.36 (2H, t, *J* = 7.5 Hz), 7.29 (1H, t, *J* = 7.3 Hz), 7.17 (1H, t, *J* = 7.6 Hz), 7.10 (1H, d, *J* = 7.9 Hz), 5.03 (2H, s); ^13^C NMR (151 MHz, DMSO) *δ* 161.61, 151.43, 143.45, 139.58, 136.02, 132.52, 129.24, 129.21, 128.17, 127.97, 127.94, 124.01, 121.56, 119.56, 111.12, 43.11. TOF HRMS (*m*/*z*): [M + H]^+^, calcd: 357.1315, found: 357.1315.

#### 4.3.5 (*E*)-*N*′-(1-Benzyl-2-oxoindolin-3-ylidene)-3-fluoro-4-methylbenzohydrazide (9)

% Yield = 92, light yellow solid, m. p.: 228–230 °C, IR (KBr) cm^−1^ 3492, 2914, 2339, 1951, 1693, 1369, 1182, 843, 652, *δ*_H_ (600 MHz, DMSO-*d*_6_) 13.84 (1H, s), 7.72–7.61 (3H, m), 7.57 (1H, t, *J* = 7.8 Hz), 7.46–7.39 (3H, m), 7.36 (2H, t, *J* = 7.6 Hz), 7.34–7.27 (1H, m), 7.18 (1H, t, *J* = 7.5 Hz), 7.09 (1H, d, *J* = 7.9 Hz), 5.04 (2H, s), 2.35 (3H, s); ^13^C NMR (151 MHz, DMSO) *δ* 161.69, 143.26, 136.07, 133.03, 132.24, 129.21, 128.14, 127.91, 123.97, 121.38, 119.74, 111.09, 43.07, 14.84. TOF HRMS (*m*/*z*): [M + H]^+^, calcd: 388.1416, found: 388.1416.

#### 4.3.6 (*E*)-*N*′-(1-Benzyl-2-oxoindolin-3-ylidene)-2-(2-(trifluoromethyl)phenyl)acetohydrazide (10)

% Yield = 92, dark brown solid, m. p.: 236–238 °C, IR (KBr) cm^−1^ 3455, 3021, 2342, 1936, 1750, 1600, 1494, 1354, 1186, 850, 731, 514, *δ*_H_ (600 MHz, DMSO-*d*_6_) 13.94 (1H, s), 11.03 (3H, s), 8.43 (6H, dd, *J* = 35.2, 8.3 Hz), 8.17 (6H, d, *J* = 8.3 Hz), 7.78–7.58 (1H, m), 7.51–7.23 (6H, m), 7.11 (2H, td, *J* = 46.8, 44.7, 7.8 Hz), 5.03 (2H, d, *J* = 12.8 Hz); ^13^C NMR (151 MHz, DMSO) *δ* 164.75, 149.98, 143.44, 138.38, 138.07, 136.03, 134.83, 132.50, 129.52, 129.23, 129.21, 128.17, 128.06, 127.92, 127.79, 124.75, 124.32, 124.01, 121.55, 119.61, 111.13, 43.11. TOF HRMS (*m*/*z*): [M + H]^+^, calcd: 438.1429, found: 438.1429.

#### 4.3.7 (*E*)-*N*′-(1-Benzyl-2-oxoindolin-3-ylidene)benzohydrazide (11)

% Yield = 88, yellow solid, m. p.: 195–197 °C, IR (KBr) cm^−1^ 3483, 3196, 2839, 1949, 1683, 1375, 1263, 1116, 788, 690, *δ*_H_ (600 MHz, DMSO-*d*_6_) 13.89 (1H, s), 7.99–7.90 (2H, m), 7.74–7.59 (4H, m), 7.45–7.38 (3H, m), 7.38–7.32 (2H, m), 7.32–7.27 (1H, m), 7.17 (1H, td, *J* = 7.5, 0.9 Hz), 7.09 (1H, d, *J* = 8.0 Hz), 5.03 (2H, s); ^13^C NMR (151 MHz, DMSO) *δ* 161.72, 143.18, 136.09, 133.44, 132.44, 132.12, 129.69, 129.21, 128.14, 127.96, 127.93, 127.90, 123.92, 121.32, 119.80, 111.03, 43.08. TOF HRMS (*m*/*z*): [M + H]^+^, calcd: 356.1399, found: 356.1396.

#### 4.3.8 (*E*)-*N*′-(1-Benzyl-2-oxoindolin-3-ylidene)-2-(4-(trifluoromethyl)phenyl)acetohydrazide (12)

% Yield = 88, light yellow solid, m. p.: 173–175 °C, IR (KBr) cm^−1^ 3456, 3022, 2342, 1956, 1754, 1610, 1344, 1176, 854, 723, 510, *δ*_H_ (600 MHz, DMSO-*d*_6_) 13.93 (1H, s), 8.13 (2H, d, *J* = 8.1 Hz), 8.02 (2H, d, *J* = 8.0 Hz), 7.68 (1H, s), 7.43 (3H, dd, *J* = 8.9, 7.4 Hz), 7.36 (2H, t, *J* = 7.7 Hz), 7.32–7.26 (1H, m), 7.18 (1H, t, *J* = 7.5 Hz), 7.10 (1H, d, *J* = 7.9 Hz), 5.03 (2H, s); ^13^C NMR (151 MHz, DMSO) *δ* 161.66, 143.38, 136.31, 136.05, 132.40, 129.21, 128.16, 127.92, 126.66, 125.11, 123.99, 121.49, 119.65, 111.10, 43.10. TOF HRMS (*m*/*z*): [M + H]^+^, calcd: 438.1429, found: 438.1429.

#### 4.3.9 (*E*)-*N*′-(1-Benzyl-2-oxoindolin-3-ylidene)-1-naphthohydrazide (13)

% Yield = 93, light yellow solid, m. p.: 146–148 °C, IR (KBr) cm^−1^ 3482, 3164, 2764, 2372, 1665, 1464, 1210, 783, 664, *δ*_H_ (600 MHz, DMSO-*d*_6_) 13.54 (1H, s), 8.41 (1H, s), 8.20 (1H, d, *J* = 8.3 Hz), 8.12–8.04 (1H, m), 7.95 (1H, s), 7.73–7.59 (4H, m), 7.45–7.36 (3H, m), 7.33 (2H, t, *J* = 7.5 Hz), 7.30–7.23 (1H, m), 7.17 (1H, s), 7.07 (1H, d, *J* = 7.9 Hz), 4.98 (2H, s); ^13^C NMR (151 MHz, DMSO) *δ* 161.46, 143.21, 136.06, 133.83, 132.17, 131.24, 130.35, 129.21, 129.18, 129.13, 129.07, 128.12, 127.94, 127.93, 127.91, 127.25, 126.88, 125.56, 125.39, 123.89, 119.76, 111.00, 43.02. TOF HRMS (*m*/*z*): [M + H]^+^, calcd: 406.1555, found: 406.1555.

#### 4.3.10 (*E*)-*N*′-(1-Benzyl-2-oxoindolin-3-ylidene)-4-bromobenzohydrazide (14)

% Yield = 83, light yellow solid, m. p.: 218–220 °C, IR (KBr) cm^−1^ 3418, 3210, 3068, 2341, 1654, 1468, 1313, 1145, 743, 567, *δ*_H_ (600 MHz, DMSO-*d*_6_) 13.87 (1H, s), 7.87 (4H, d, *J* = 1.6 Hz), 7.68 (1H, d, *J* = 7.5 Hz), 7.42 (3H, td, *J* = 7.8, 1.2 Hz), 7.39–7.33 (2H, m), 7.33–7.26 (1H, m), 7.21–7.14 (1H, m), 7.10 (1H, d, *J* = 7.9 Hz), 5.03 (2H, s); ^13^C NMR (151 MHz, DMSO) *δ* 161.69, 143.28, 136.07, 132.75, 132.26, 131.59, 129.21, 128.15, 127.92, 123.97, 121.40, 119.73, 111.08, 43.08. TOF HRMS (*m*/*z*): [M + H]^+^, calcd: 434.0504, found: 434.0504.

#### 4.3.11 (*E*)-*N*′-(1-Benzyl-2-oxoindolin-3-ylidene)-2-methylbenzohydrazide (15)

% Yield = 95, yellow solid, m. p.: 148–150 °C, IR (KBr) cm^−1^ 3435, 3120, 2336, 1810, 1691, 1503, 1376, 1257, 900, 697, 586, *δ*_H_ (600 MHz, DMSO-*d*_6_) 13.33 (1H, s), 7.62 (2H, s), 7.50 (1H, t, *J* = 7.5 Hz), 7.43–7.32 (7H, m), 7.33–7.26 (1H, m), 7.20–7.11 (1H, m), 7.07 (1H, d, *J* = 7.9 Hz), 4.99 (2H, s), 2.47 (3H, s); ^13^C NMR (151 MHz, DMSO) *δ* 161.44, 143.15, 136.08, 133.56, 132.07, 129.18, 128.12, 127.91, 127.85, 127.74, 123.87, 121.18, 119.79, 110.99, 43.02, 20.14. TOF HRMS (*m*/*z*): [M + H]^+^, calcd: 370.1555, found: 370.1555.

#### 4.3.12 (*E*)-*N*′-(1-Benzyl-2-oxoindolin-3-ylidene)furan-2-carbohydrazide (16)

% Yield = 93, light yellow solid, m. p.: 188–190 °C, IR (KBr) cm^−1^ 2463, 3022, 2343, 1797, 1595, 1392, 1259, 1147, 903, 720, *δ*_H_ (600 MHz, DMSO-*d*_6_) 13.81 (1H, s), 8.08 (1H, dd, *J* = 1.7, 0.8 Hz), 7.72–7.65 (1H, m), 7.47 (1H, s), 7.45–7.39 (3H, m), 7.38–7.33 (2H, m), 7.32–7.27 (1H, m), 7.17 (1H, td, *J* = 7.6, 0.9 Hz), 7.09 (1H, dt, *J* = 8.0, 0.8 Hz), 6.81 (1H, dd, *J* = 3.6, 1.7 Hz), 5.02 (2H, s); ^13^C NMR (151 MHz, DMSO) *δ* 161.58, 147.55, 143.25, 136.11, 132.15, 129.21, 128.14, 127.97, 123.87, 121.38, 119.77, 113.49, 111.00, 43.08. TOF HRMS (*m*/*z*): [M + H]^+^, calcd: 346.1191, found: 346.1191.

#### 4.3.13 (*E*)-*N*′-(1-Benzyl-2-oxoindolin-3-ylidene)-4-(trifluoromethyl)benzohydrazide (17)

% Yield = 90, yellow solid, m. p.: 172–174 °C, IR (KBr) cm^−1^ 3456, 3042, 2346, 1948, 1750, 1356, 1185, 997, 864, 732, *δ*_H_ (600 MHz, DMSO-*d*_6_) 13.93 (1H, s), 8.13 (2H, d, *J* = 8.1 Hz), 8.02 (2H, d, *J* = 8.1 Hz), 7.68 (1H, s), 7.45–7.39 (3H, m), 7.38–7.33 (2H, m), 7.32–7.27 (1H, m), 7.17 (1H, t, *J* = 7.6 Hz), 7.10 (1H, d, *J* = 7.9 Hz), 5.03 (2H, s); ^13^C NMR (151 MHz, DMSO) *δ* 161.65, 143.37, 136.30, 136.05, 132.40, 129.24, 129.21, 129.19, 128.16, 127.92, 126.66, 125.10, 123.99, 123.30, 121.48, 119.64, 111.10, 43.10. TOF HRMS (*m*/*z*): [M + H]^+^, calcd: 424.1272, found: 424.1272.

#### 4.3.14 (*Z*)-*N*′-(1-Benzyl-2-oxoindolin-3-ylidene)nicotinohydrazide (18)

% Yield = 85, yellow solid, m. p.: 158–160 °C, IR (KBr) cm^−1^ 3490, 3054, 2346, 1946, 1820, 1654, 1265, 754, 676, *δ*_H_ (600 MHz, DMSO-*d*_6_) 13.86 (1H, s), 9.10 (1H, d, *J* = 2.3 Hz), 8.88–8.83 (1H, m), 8.29 (1H, dt, *J* = 8.0, 2.0 Hz), 7.66 (2H, dd, *J* = 8.0, 4.8 Hz), 7.43–7.39 (3H, m), 7.35 (2H, t, *J* = 7.6 Hz), 7.32–7.27 (1H, m), 7.16 (1H, t, *J* = 7.6 Hz), 7.09 (1H, d, *J* = 7.9 Hz), 5.03 (2H, s); ^13^C NMR (151 MHz, DMSO) *δ* 161.58, 153.68, 148.89, 148.56, 143.31, 136.05, 135.66, 135.15, 132.34, 129.21, 128.45, 128.16, 127.94, 123.97, 121.42, 119.63, 111.08, 43.09. TOF HRMS (*m*/*z*): [M + H]^+^, calcd 357.1351, found: 357.1351.

#### 4.3.15 (*E*)-*N*′-(1-Benzyl-2-oxoindolin-3-ylidene)-2-(2-((2,6-dichlorophenyl)amino)phenyl) acetohydrazide (19)

% Yield = 90, light yellow solid, m. p.: 202–204 °C, IR (KBr) cm^−1^ 3483, 3063, 1948, 1688, 1356, 1156, 734, 423, *δ*_H_ (600 MHz, DMSO-*d*_6_) 11.58 (1H, s), 8.26 (1H, s), 7.65 (1H, d, *J* = 38.7 Hz), 7.53 (2H, dd, *J* = 0.1, 3.8 Hz), 7.44–7.31 (6H, m), 7.31–7.17 (2H, m), 7.08 (2H, q, *J* = 9.5, 8.4 Hz), 7.03 (1H, d, *J* = 8.0 Hz), 6.87 (1H, d, *J* = 14.8 Hz), 6.36–6.16 (1H, m), 4.96 (2H, s), 4.18 (2H, d, *J* = 99.7 Hz); ^13^C NMR (151 MHz, DMSO) *δ* 163.94, 144.30, 143.61, 143.15, 137.42, 136.59, 136.12, 131.97, 131.80, 131.04, 129.65, 129.62, 129.20, 129.18, 128.21, 128.11, 127.99, 127.84, 127.72, 126.50, 126.33, 123.75, 122.84, 121.16, 120.79, 116.28, 115.20, 110.93, 110.37, 43.15, 42.98. TOF HRMS (*m*/*z*): [M + H]^+^, calcd: 529.1198, found: 529.1198.

#### 4.3.16 (*E*)-*N*′-(1-Benzyl-2-oxoindolin-3-ylidene)-2-chlorobenzohydrazide (20)

% Yield = 90, light yellow solid, m. p.: 193–195 °C, IR (KBr) cm^−1^ 3493, 3212, 3051, 2335, 1933, 1691, 1336, 1148, 747, 573, 432, *δ*_H_ (600 MHz, DMSO-*d*_6_) 13.36 (1H, s), 7.67 (4H, dd, *J* = 89.7, 56.2 Hz), 7.48–7.31 (5H, m), 7.32–7.25 (1H, m), 7.17 (1H, s), 7.06 (1H, s), 4.98 (2H, s); ^13^C NMR (151 MHz, DMSO) *δ* 169.58, 163.05, 161.37, 143.36, 136.03, 133.35, 132.44, 130.97, 130.21, 129.23, 129.19, 128.33, 128.13, 127.91, 123.94, 121.49, 119.61, 111.05, 43.07. TOF HRMS (*m*/*z*): [M + H]^+^, calcd: 390.1009, found: 390.1009.

#### 4.3.17 (*E*)-*N*′-(1-Benzyl-2-oxoindolin-3-ylidene)-2-(2,4-difluorophenyl)acetohydrazide (21)

% Yield = 90, light yellow solid, m. p.: 179–181 °C, IR (KBr) cm^−1^ 3480, 3212, 2937, 2348, 1698, 1463, 1363, 1175, 974, 760, 512, *δ*_H_ (600 MHz, DMSO-*d*_6_) 12.53 (1H, s), 7.68–7.56 (1H, m), 7.49 (1H, q, *J* = 7.9 Hz), 7.42–7.32 (5H, m), 7.31–7.22 (2H, m), 7.14 (1H, d, *J* = 7.6 Hz), 7.12–7.07 (1H, m), 7.06 (1H, d, *J* = 8.0 Hz), 4.99 (2H, s), 4.22 (1H, s), 3.92 (1H, s); ^13^C NMR (151 MHz, DMSO) *δ* 172.31, 162.82, 162.06, 161.98, 161.14, 160.42, 160.34, 143.16, 136.12, 133.85, 133.73, 133.69, 133.66, 133.62, 131.88, 129.23, 129.19, 128.11, 127.86, 123.74, 120.93, 119.74, 111.83, 110.94, 104.13, 43.00, 31.86. TOF HRMS (*m*/*z*): [M + H]^+^, calcd: 406.1367, found: 406.1367.

#### 4.3.18 (*E*)-*N*′-(1-Benzyl-2-oxoindolin-3-ylidene)-2-bromobenzohydrazide (22)

% Yield = 85, light yellow solid, m. p.: 90–92 °C, IR (KBr) cm^−1^ 3427, 3206, 3079, 2314, 1686, 1471, 1324, 1143, 728, 580, *δ*_H_ (600 MHz, DMSO-*d*_6_) 13.28 (1H, s), 7.88–7.64 (2H, m), 7.55 (2H, d, *J* = 25.8 Hz), 7.34 (6H, ddd, *J* = 35.2, 25.7, 7.3 Hz), 7.24–7.11 (1H, m), 7.07 (1H, t, *J* = 8.4 Hz), 4.98 (2H, s); ^13^C NMR (151 MHz, DMSO) *δ* 163.92, 161.38, 143.33, 138.24, 136.02, 135.76, 134.12, 133.31, 132.45, 132.11, 130.00, 129.20, 129.18, 128.75, 128.14, 127.92, 123.96, 121.50, 119.75, 119.57, 111.06, 43.08. TOF HRMS (*m*/*z*): [M + H]^+^, calcd: 434.0504, found: 434.0504.

#### 4.3.19 (*E*)-1-Benzyl-3-((2-(4-nitrophenyl)-2-oxoethyl)imino)indolin-2-one (23)

% Yield = 85, light yellow solid, m. p.: 174–176 °C, IR (KBr) cm^−1^ 3463, 3113, 2366, 1950, 1614, 1384, 1160, 957, 733, *δ*_H_ (600 MHz, DMSO-*d*_6_) 12.57 (1H, s), 7.82–7.48 (5H, m), 7.35 (6H, ddd, *J* = 36.7, 25.8, 7.7 Hz), 7.21–6.96 (2H, m), 4.99 (2H, s), 4.41 (1H, s), 4.08 (1H, s); ^13^C NMR (151 MHz, DMSO) *δ* 172.60, 161.17, 143.17, 136.63, 136.11, 133.92, 133.41, 132.89, 131.90, 129.19, 128.52, 128.33, 128.11, 127.87, 126.32, 125.84, 124.02, 123.74, 121.18, 120.86, 119.71, 110.96, 43.01, 35.77. TOF HRMS (*m*/*z*): [M + H]^+^, calcd: 401.1249, found: 401.1249.

#### 4.3.20 (*E*)-*N*′-(1-Benzyl-2-oxoindolin-3-ylidene)-2-phenylacetohydrazide (24)

% Yield = 85, light yellow solid, m. p.: 157–159 °C, IR (KBr) cm^−1^ 3443, 3164, 2842, 2355, 1943, 1664, 1372, 1263, 1175, 762, 664, *δ*_H_ (600 MHz, DMSO-*d*_6_) 12.48 (1H, s), 7.64 (1H, d, *J* = 53.6 Hz), 7.45–7.21 (11H, m), 7.15 (1H, s), 7.05 (1H, d, *J* = 7.9 Hz), 4.98 (2H, s), 4.16 (1H, s), 3.86 (1H, s); ^13^C NMR (151 MHz, DMSO) *δ* 161.10, 143.10, 136.13, 131.81, 130.06, 129.48, 129.22, 129.19, 128.87, 128.68, 128.10, 127.85, 123.73, 119.81, 110.91, 42.99, 38.36. TOF HRMS (*m*/*z*): [M + H]^+^, calcd: 370.1555, found: 370.1555.

#### 4.3.21 (*E*)-*N*′-(1-Benzyl-2-oxoindolin-3-ylidene)-3-hydroxy-2-naphthohydrazide (25)

% Yield = 93, m. p.: light yellow solid, 146–148 °C, IR (KBr) cm^−1^ 3645, 3386, 2784, 2448, 1692, 1496, 1201, 900, 753, *δ*_H_ (600 MHz, DMSO-*d*_6_) 13.54 (1H, s), 8.41 (1H, s), 8.20 (1H, d, *J* = 8.3 Hz), 8.12–8.04 (1H, m), 7.95 (1H, s), 7.73–7.59 (4H, m), 7.45–7.36 (3H, m), 7.33 (2H, t, *J* = 7.5 Hz), 7.30–7.23 (1H, m), 7.17 (1H, s), 7.07 (1H, d, *J* = 7.9 Hz), 4.98 (2H, s); ^13^C NMR (151 MHz, DMSO) *δ* 161.46, 143.21, 136.06, 133.83, 132.17, 131.24, 130.35, 129.21, 129.18, 129.13, 129.07, 128.12, 127.94, 127.93, 127.91, 127.25, 126.88, 125.56, 125.39, 123.89, 119.76, 111.00, 43.02. TOF HRMS (*m*/*z*): [M + H]^+^, calcd: 422.1506, found: 422.1506.

### 4.4. *In vitro* cytotoxicity assay of synthetic derivatives

*In vitro*, cytotoxicity assay for the synthetic derivatives was determined by performing an MTT (yellow tetrazolium salt, 3-(4,5-dimethylthizol-2-yl)-2,5-diphenyl tetrazolium bromide) assay by using an aggressive breast cancer cell line MDA-MB-231.^51^ Human breast normal cell line MCF-10A was kept as a control in the study.^52,53^ Cells were cultured in DMEM augmented with 10% FBS and 1% antibiotics (100 U per mL penicillin). The cells were sowed in a 96-well plate at a 1.0 × 10^4^ cells per well density and incubated for 24 h at 37 °C in 5% CO_2_. The medium was discarded, and both the cell lines were treated with different concentrations (6.5 μM, 12.5 μM, 25 μM, and 50 μM) of synthetic hydrazone derivatives.^54^ After 48 h of incubation 20 μL of MTT solution (5 mg mL^−1^) was pipetted into each well and incubated for another 4 h.^55^ Later the medium was discarded, and the precipitate of formazan was dissolved in DMSO. The absorbance of the mixtures was calculated using a microplate reader at 570 nm. Triplicates were used for performing all experiments and the cytotoxicity was expressed as a percentage of cell viability compared to untreated control cells.^54^1
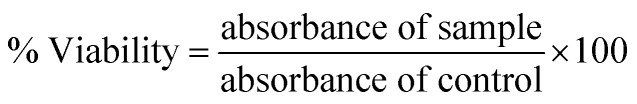


### 4.5. Softwares used

In this work we have utilized different Softwares for computational calculations to analyze protein–ligand binding, binding stability of protein–ligand complex, ADMET properties of ligands and for preparing both protein and ligand to assign the 3D coordinates. We have utilized LigPrep, Schrodinger, LLC, NY, 2023 and ‘Protein Preparation Workflow’ Schrodinger, LLC, NY, 2023 for preparing ligand and protein for assigning 3D coordinates and protonation state. The OPLS-2005 force field was used throughout the calculations. The conformation generation was set to 32 for ligands and only least energy conformers were used for docking analyses.^56^ Glide module, V. 2023 for defining binding site and molecular docking study, ‘Desmond’ module to perform molecular dynamics for simulating and analyzing stability and interactions of protein–ligand complex were used. QikProp, Schrodinger, LLC, NY, 2023 module was used for analyzing the ADMET properties of ligands synthesized. All the mentioned modules used are available from Drug Discovery Package, Schrödinger, Inc., New York, USA (2024) software.

### 4.6. Molecular docking and MMGBSA

Epidermal growth factor receptor (EGFR) is a membrane protein often targeted by anticancer drugs due to its function in cell proliferation.^57^ It has several domains including the tyrosine kinase domain which activates the cancer signaling mechanism by phosphorylation. Targeting the EGFR kinase domain is important to address the cancer. The X-ray crystal structure of EGFR kinase domain (PDB ID 1M17) was taken by using Protein Data Bank with a resolution of 2.60 Å (https://www.rcsb.org/structure/1m17) and prepared using protein preparation workflow in a molecular dynamics software known as Schrodinger developed by the company with the same name Schrodinger Inc., New York, USA (2024) using physiological pH of 7.4 to add missing hydrogens, adjusting the protonation of amino acids and to optimize the structure.^58,59^ The newly synthesized molecules were drawn using MarvinSketch 24.1.2 to obtain the 2D structure of ligands. The ligands were prepared at the same pH for protein to add hydrogens, assign proper ionization state, and obtain the 3D coordinates of the ligands using the LigPrep module. We then performed the docking studies using the XP-docking protocol (extra precision) with all other default parameters.^60^

### 4.7. Molecular dynamics simulation

Molecular dynamics simulation (MDS)^51–55,57–60^ was executed using Desmond's module from Schrödinger, Inc., New York, USA (2024).^56^ The docked complex was embedded in an explicit solvent through SPC water model by the OPLS4 force field. The simulation was performed in an orthorhombic periodic condition by neutralizing the charge using a NaCl salt solution. The simulation was carried out using the particle mesh Ewald (PME) method for electrostatics at 9 Å cut-off using an isobaric–isothermal ensemble (NPT) with 300 K temperature and 1 bar pressure for 100 ns. Finally, the trajectory was analyzed using RMSD, RMSF, and interaction profile throughout the simulation to evaluate the protein–ligand complex stability.

### 4.8. ADMET

For all the synthesized compounds, properties for pharmacologically relevant ADMET (absorption, distribution, elimination, and toxicity) properties were calculated using QikProp module from Schrodinger, Inc., New York, USA (2024). QikProp predicts the significant descriptors by comparing them with the top most 5 similar drug molecules.^61^ The prepared ligands were subjected to analysis and evaluate the ted according to the descriptor values.

## Abbreviations

ADME: Absorption, distribution, metabolism, and excretion
TNBC: Triple negative breast cancer
BC: Breast cancer
FDA: The United States Food and Drug Administration
FTIR: Fourier Transform Infrared Spectroscopy
NMR: Nuclear Magnetic Resonance
HRMS: High-resolution mass spectrometry
HER2: Human epidermal growth factor receptor 2
IC_50_: Half-maximal inhibitory concentration
RMSD: Root Mean Square Deviation
RMSF: Root Mean Square Fluctuation
MDs: Molecular Dynamics Simulation
MMGBSA: Molecular mechanics with generalized born and surface area solvation

## Data availability

The data used for the manuscript entitled “Design, synthesis, *in vitro*, and *in silico* studies of novel isatin-hybrid hydrazones as potential triple-negative breast cancer agents” will be included in an ESI file,† available online on *RSC Advances* web site.

## Author contributions

Conceptualization: Z. S. and A. A-H. Investigation: I. M, Z. B. Formal analysis: F. K., J. H., A. K methodology, software: V. V. R, B. M., S. N. M. Funding acquisition, resources: T. M. A. Writing original draft: Z. B., Z. S. Writing-review & editing: M. S. A.

## Conflicts of interest

The authors have declared no conflict of interest.

## Supplementary Material

RA-015-D4RA07650H-s001
