# Supplementary material for: Design, synthesis, in vitro, and in silico studies of novel isatin-hybrid hydrazones as potential triple-negative breast cancer agents
Source: RSC Adv. 2025 Jan 13;15(2):948–65. doi: 10.1039/d4ra07650h (PMC11726183; doi:10.1039/d4ra07650h)

Supplementary Information (SI) for RSC Advances.
This journal is © The Royal Society of Chemistry 2025

1HNMR, 13CNMR and mass spectra of compound 5

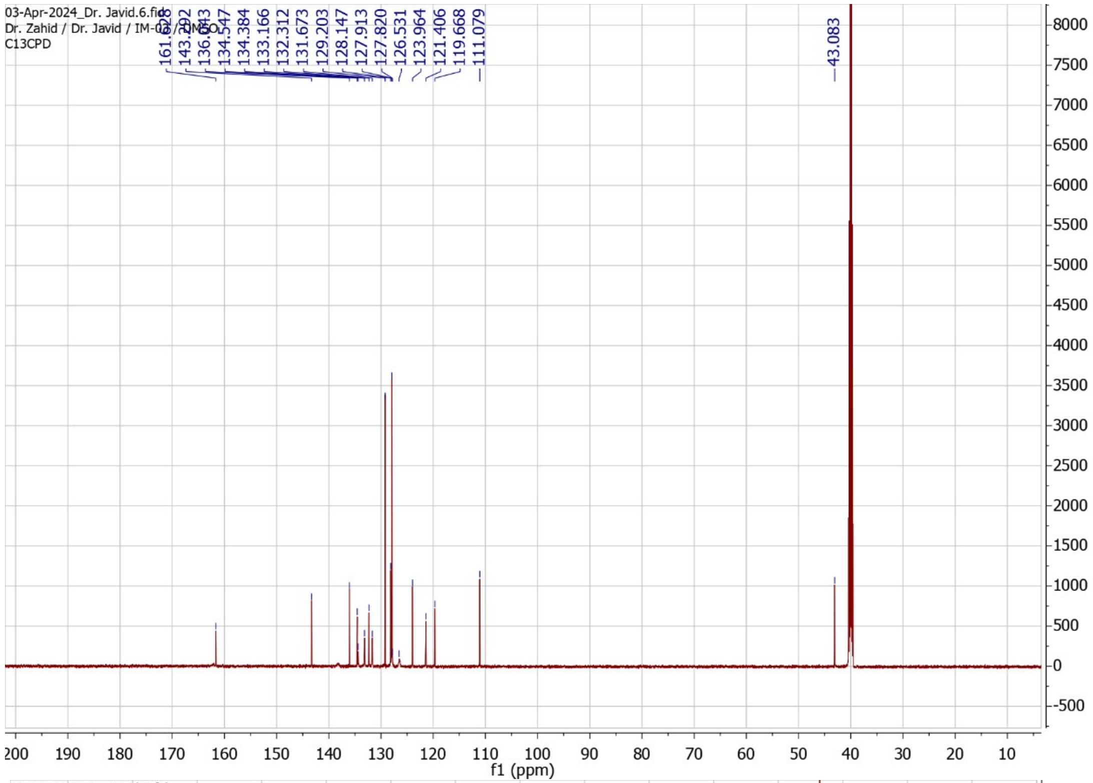

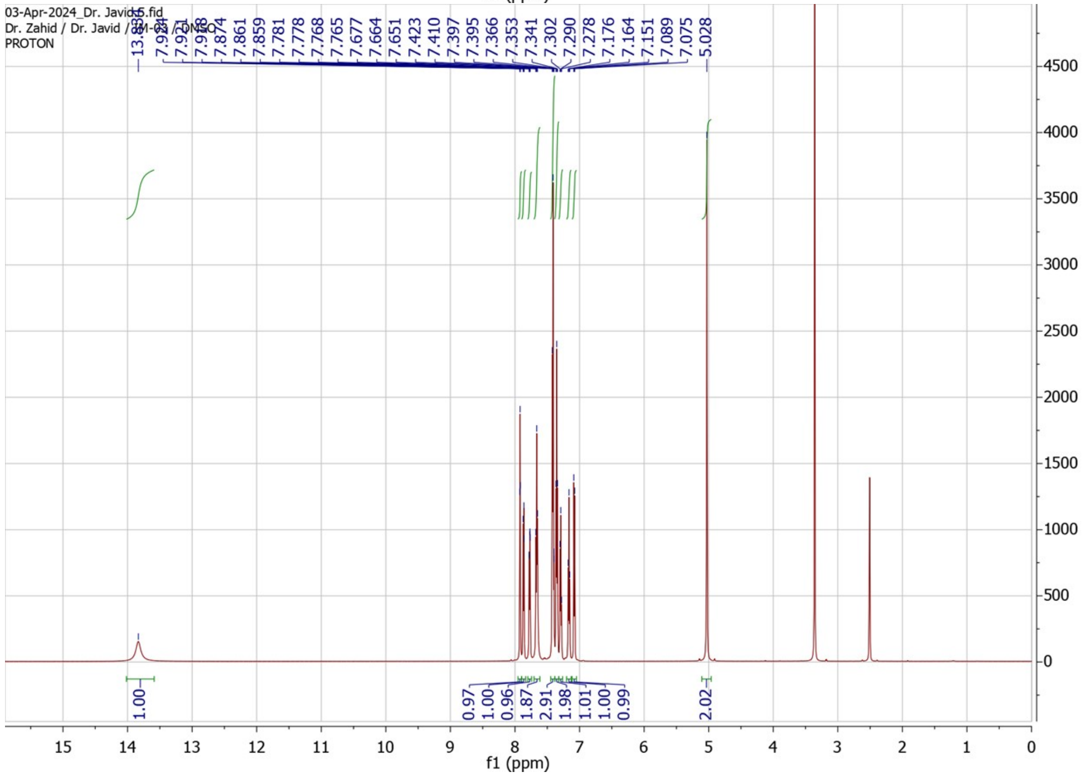

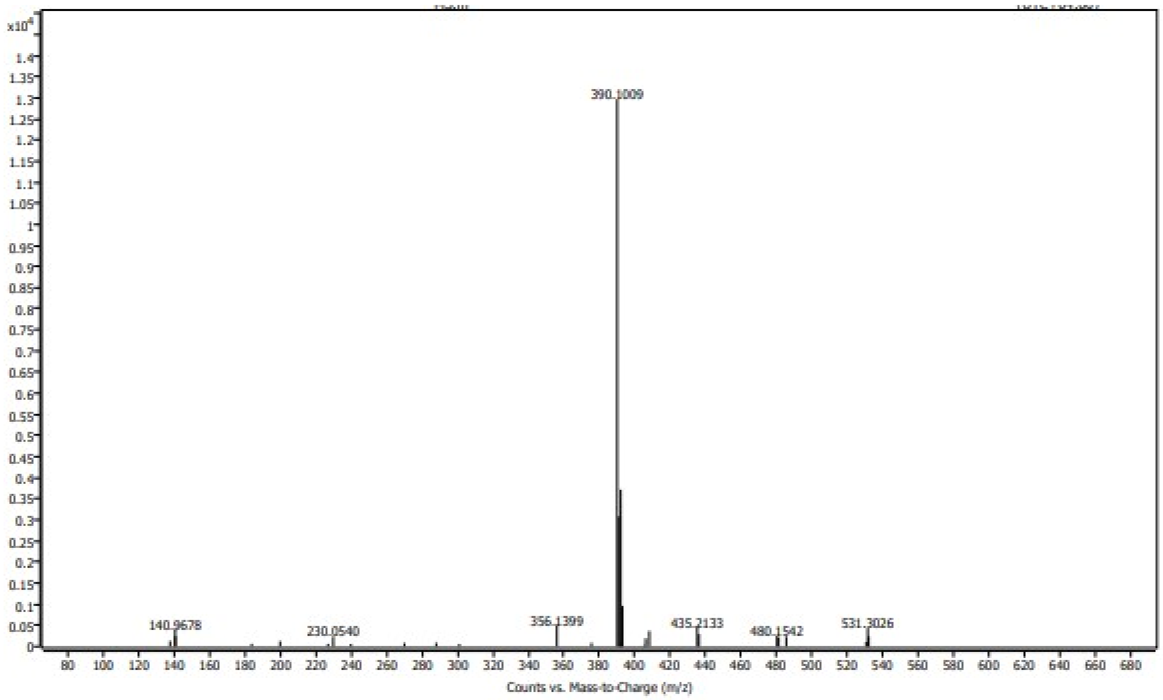

x10^4
390.1009
140.9678
230.0540
356.1399
435.2133
480.1542
531.3026
Counts vs. Mass-to-Charge (m/z)

^{1}HNMR, ^{13}CNMR, and mass spectra of compound 6

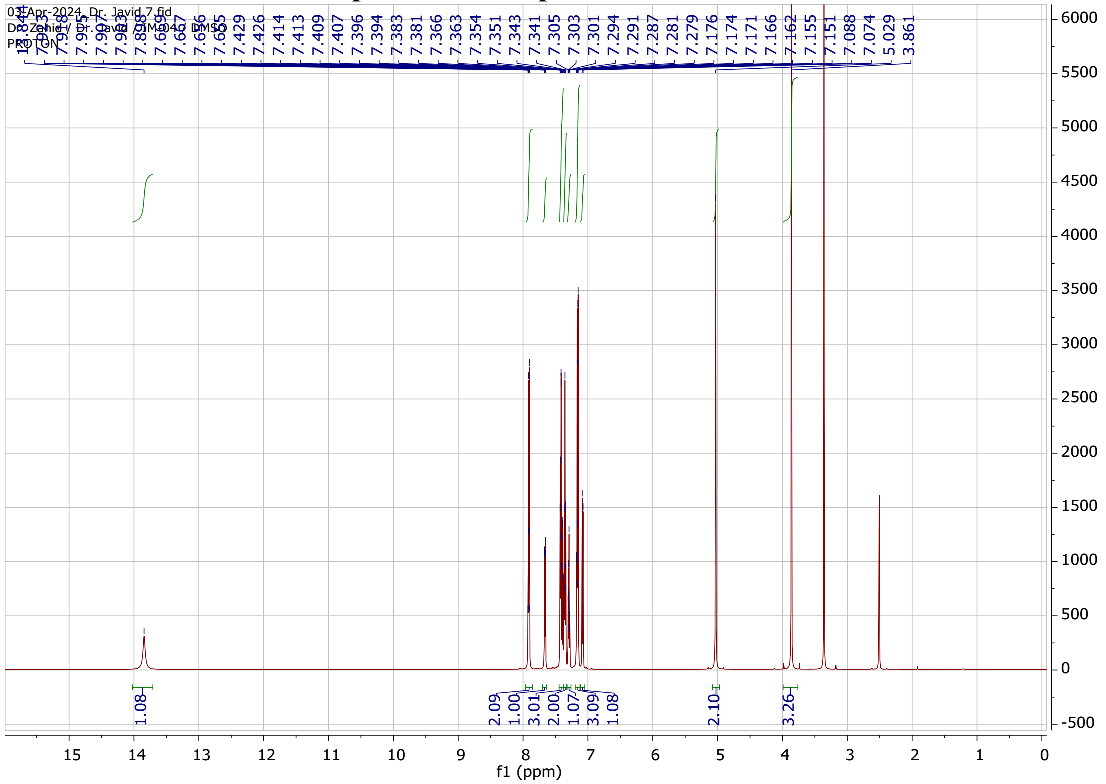

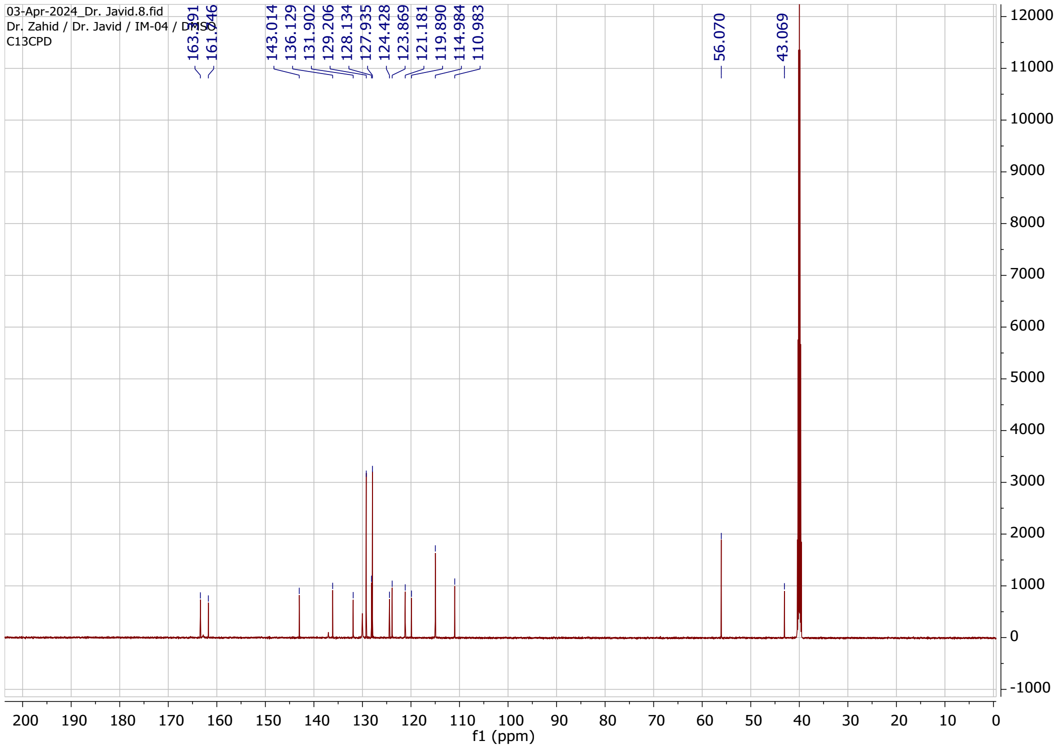

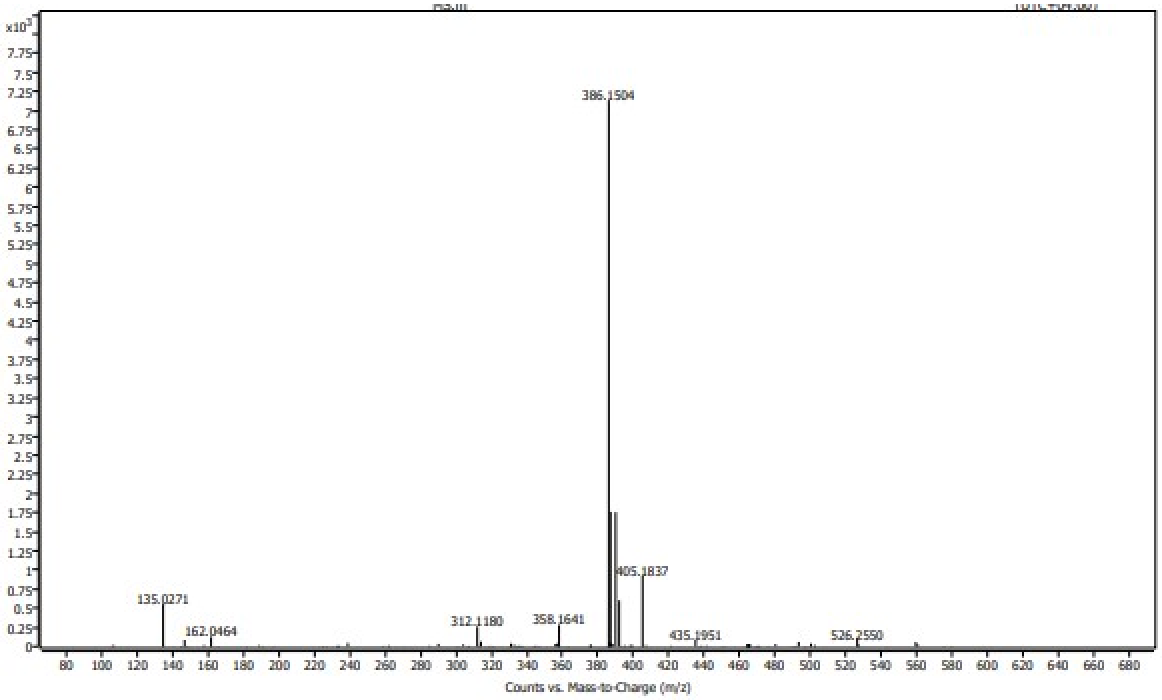

x10^3
386.1504
405.1837
135.0271
162.0464
312.1180
358.1641
435.1951
526.2550
Counts vs. Mass-to-Charge (m/z)

^{1}HNMR, ^{13}CNMR and mass spectra of compound 7

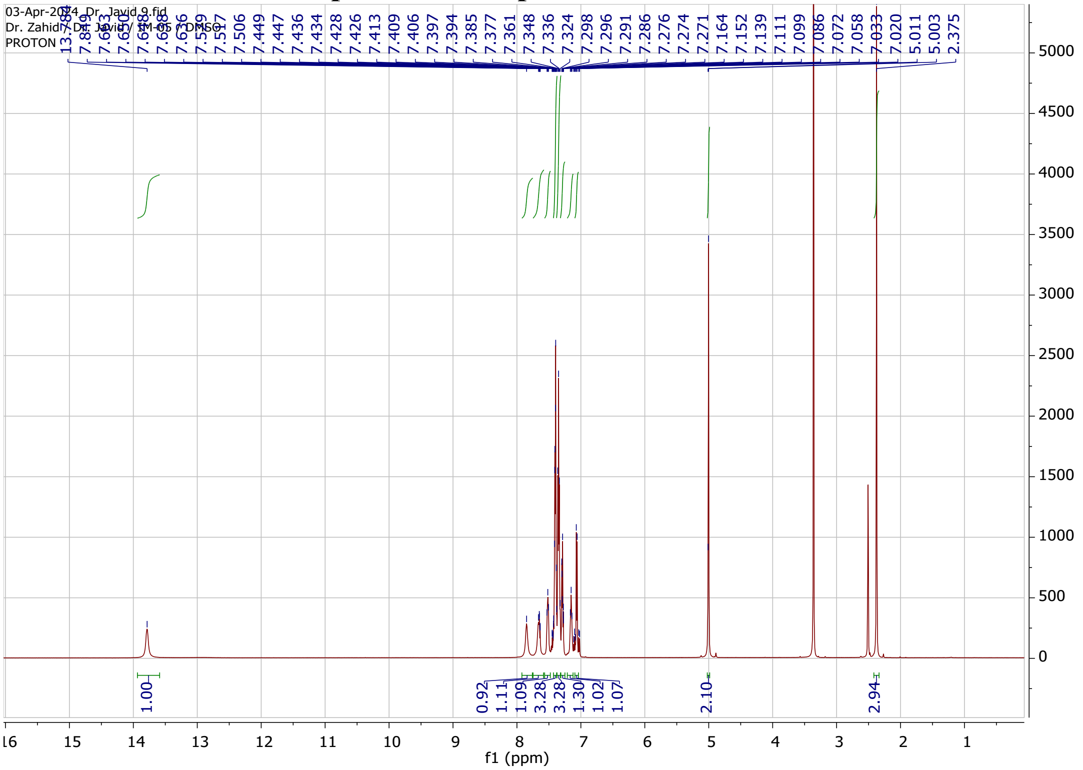

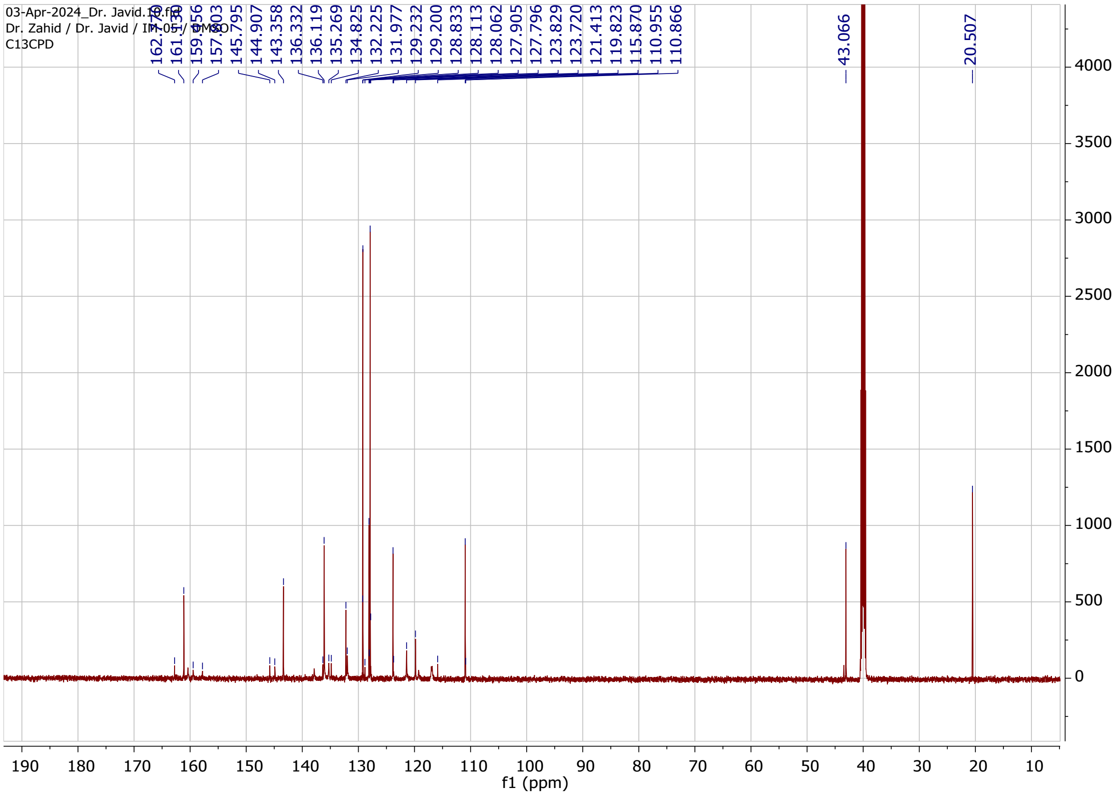

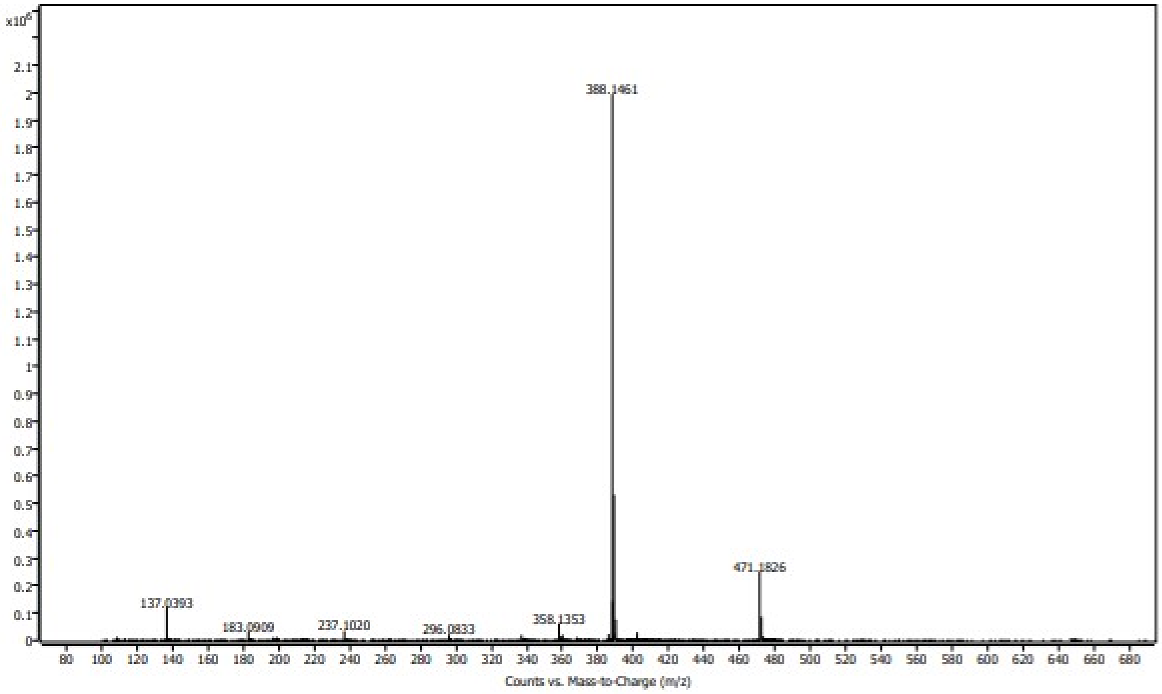

x10^6
388.1461
471.1826
137.0393
183.0909
237.1020
296.0833
358.1353
Counts vs. Mass-to-Charge (m/z)

^{1}HNMR, ^{13}CNMR and mass spectra of compound 8

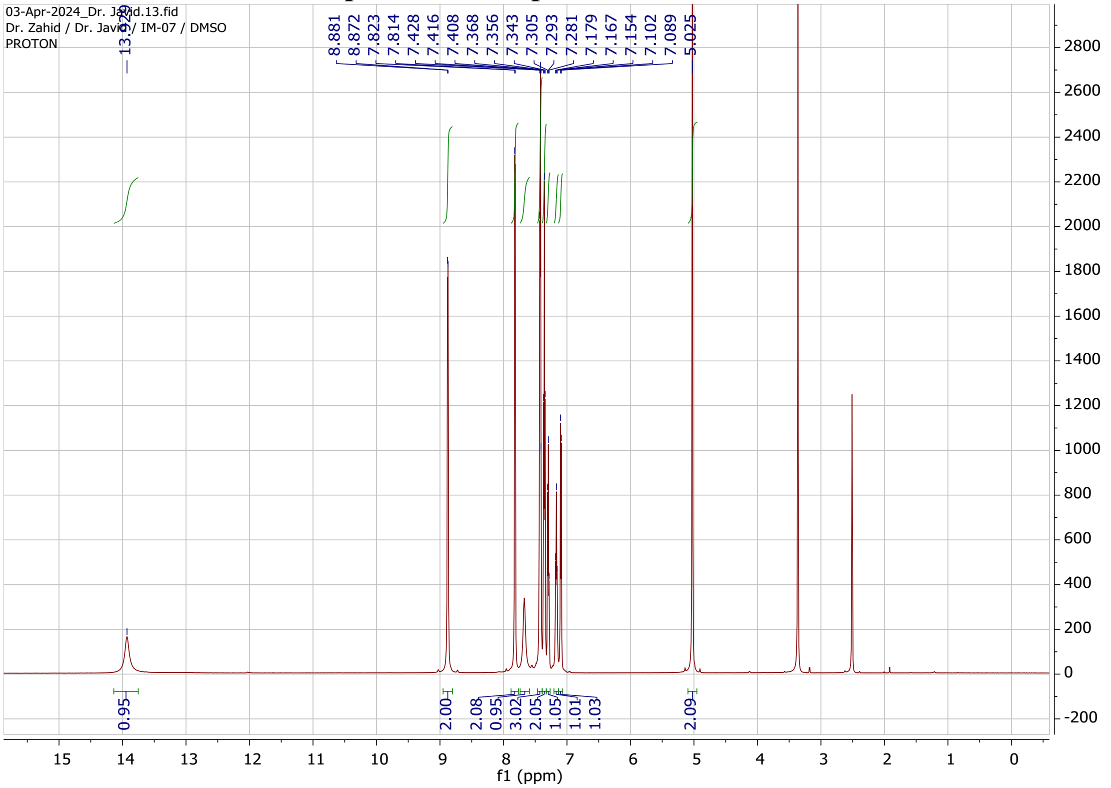

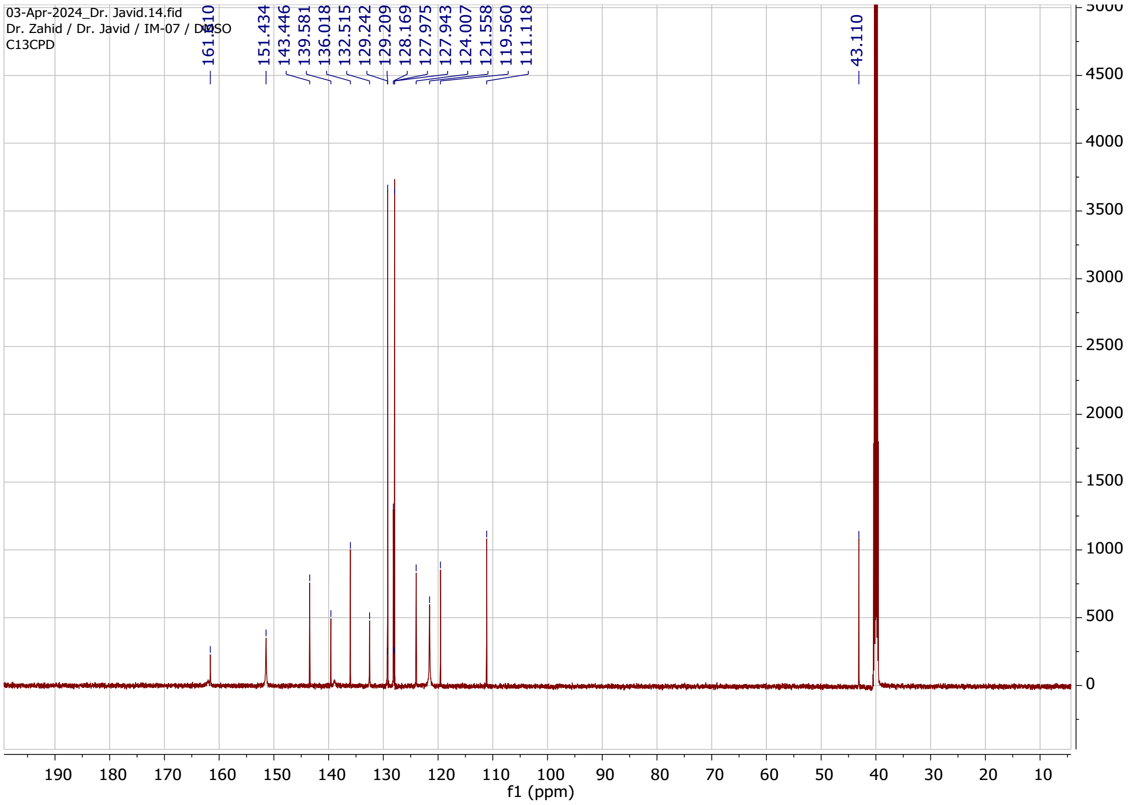

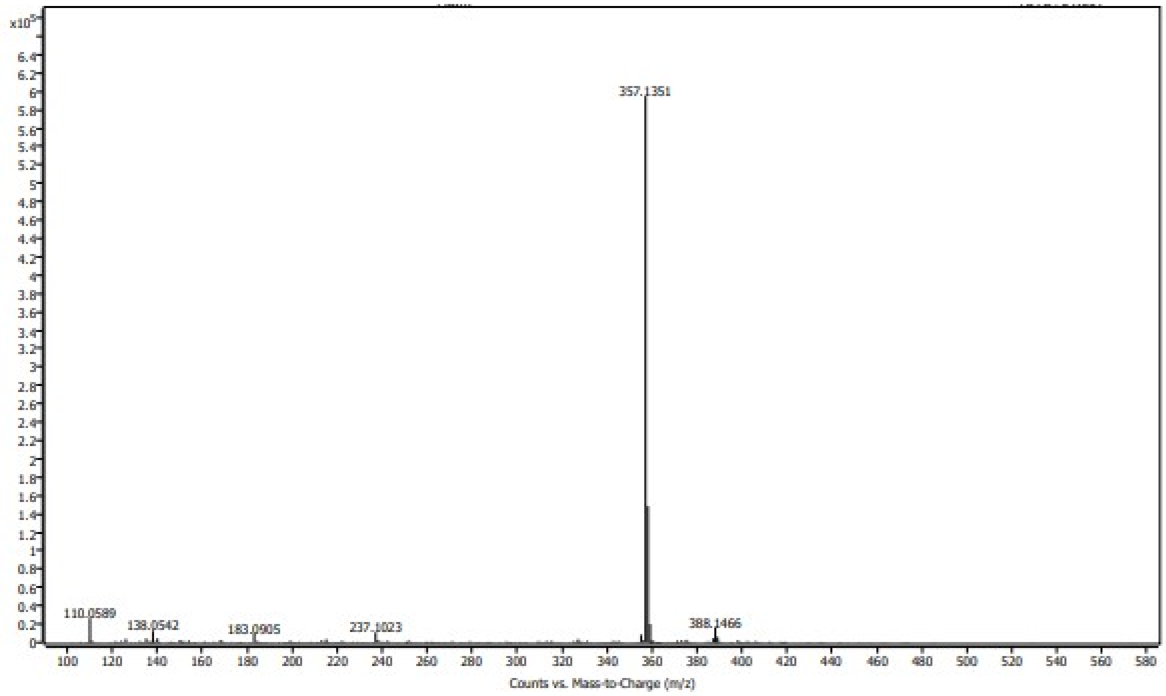

x10^5
357.1351
110.0589
138.0542
183.0905
237.1023
388.1466
Counts vs. Mass-to-Charge (m/z)

^{1}HNMR, ^{13}CNMR, and mass spectra of compound 9

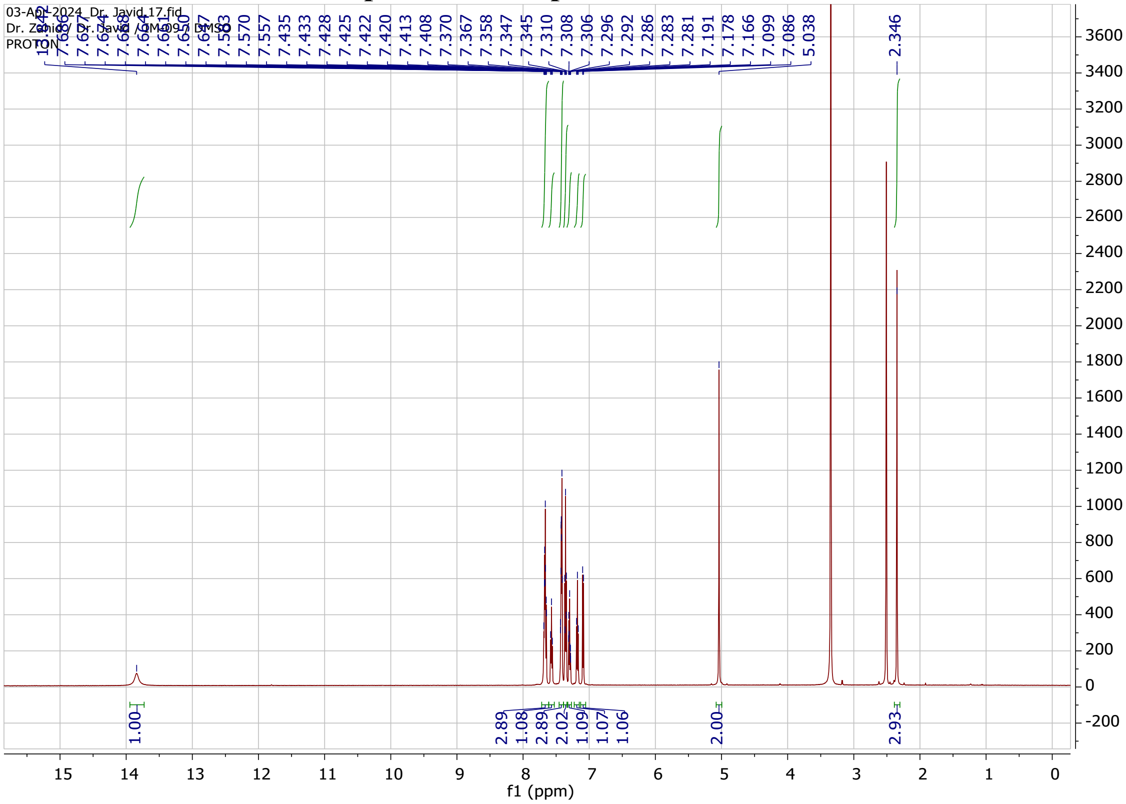

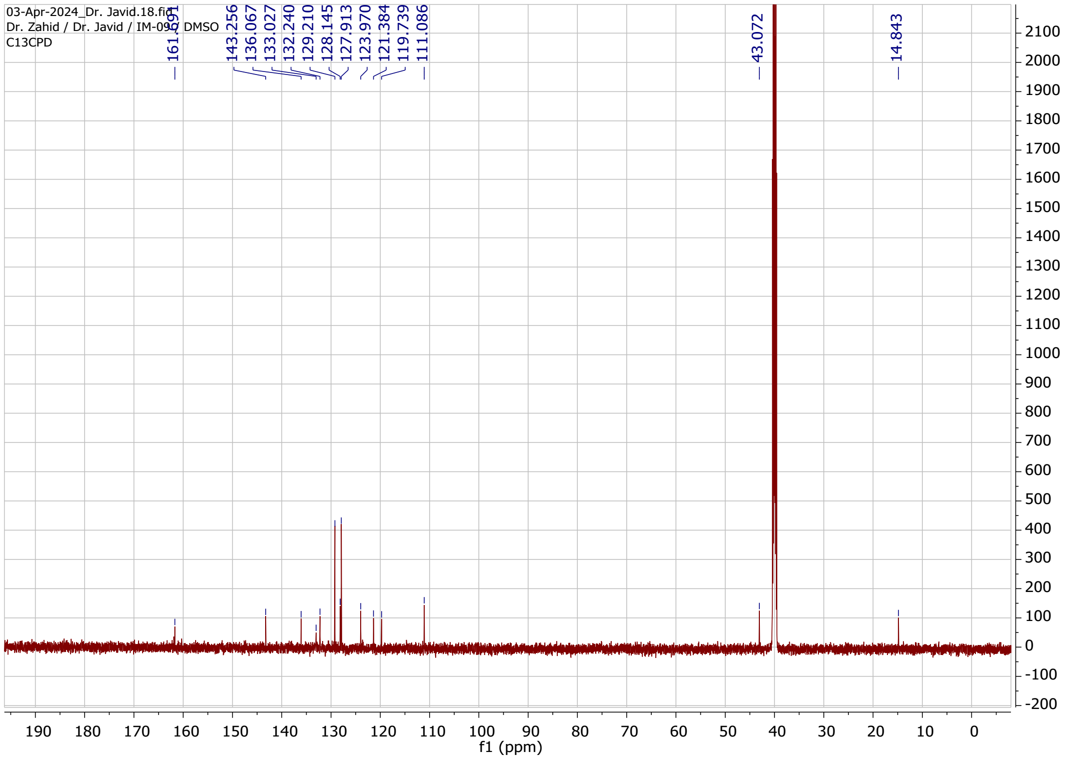

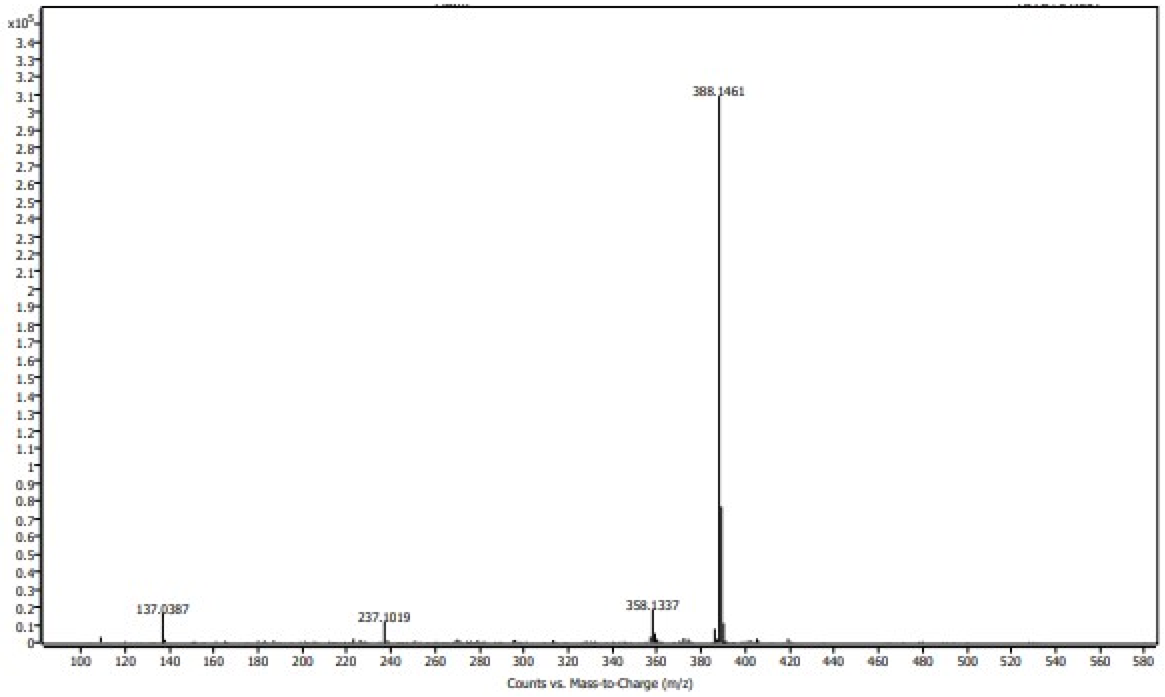

x10^5
388.1461
137.0387
237.1019
358.1337
Counts vs. Mass-to-Charge (m/z)

^{1}HNMR, ^{13}CNMR, and mass spectra of compound 10

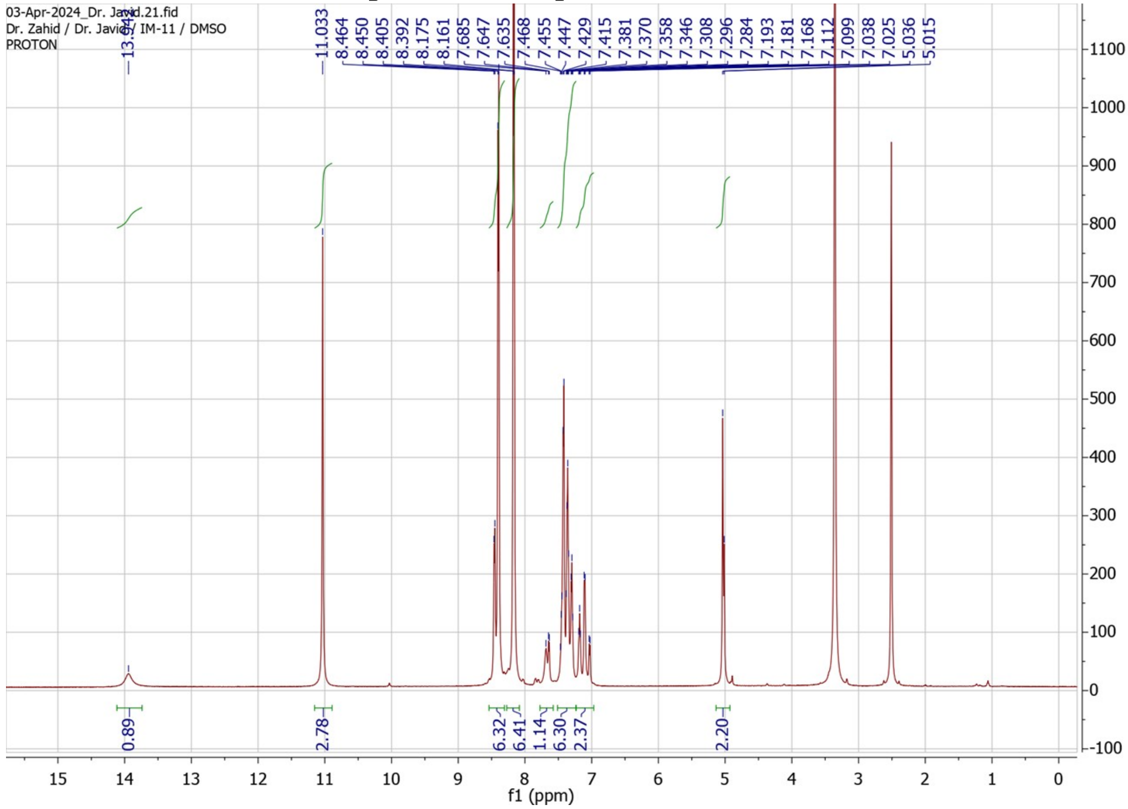

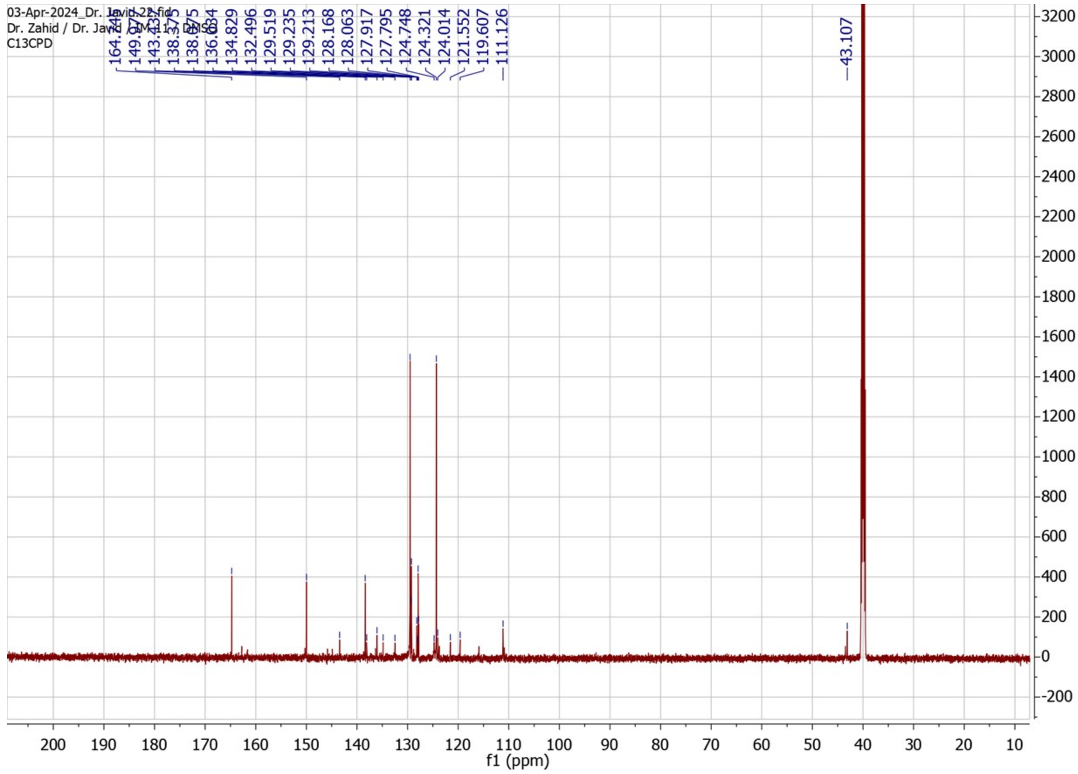

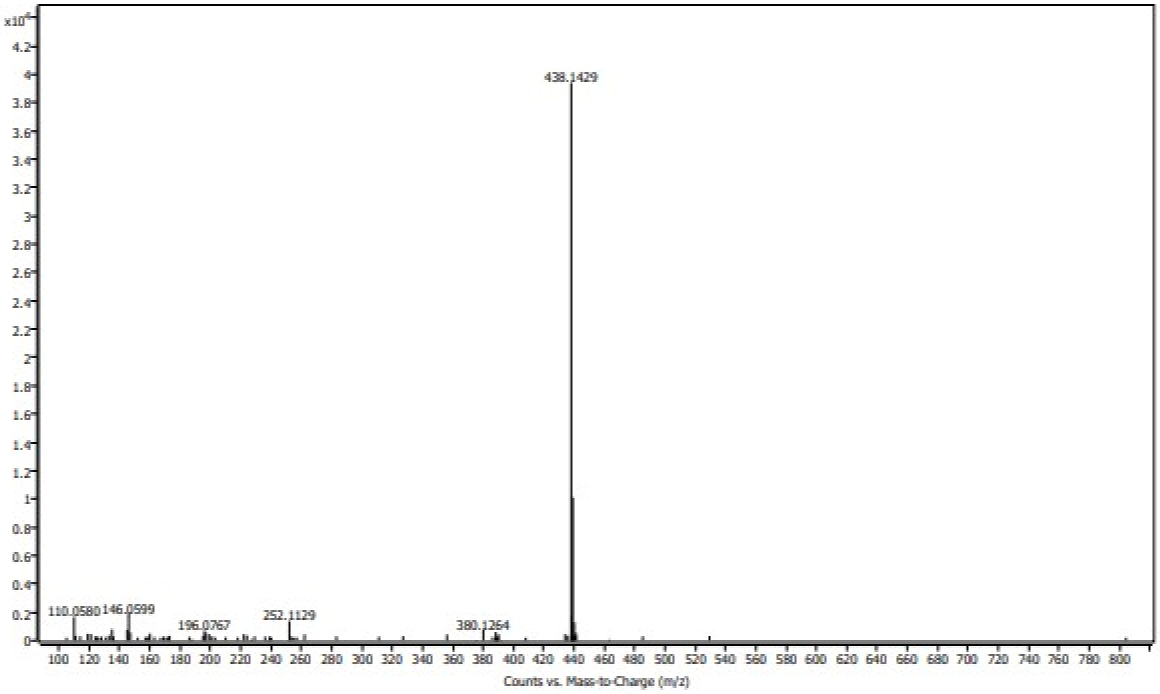
x10^4
438.1429
110.0580
146.0599
196.0767
252.1129
380.1264
Counts vs. Mass-to-Charge (m/z)

^{1}HNMR, ^{13}CNMR, and mass spectra of compound 11

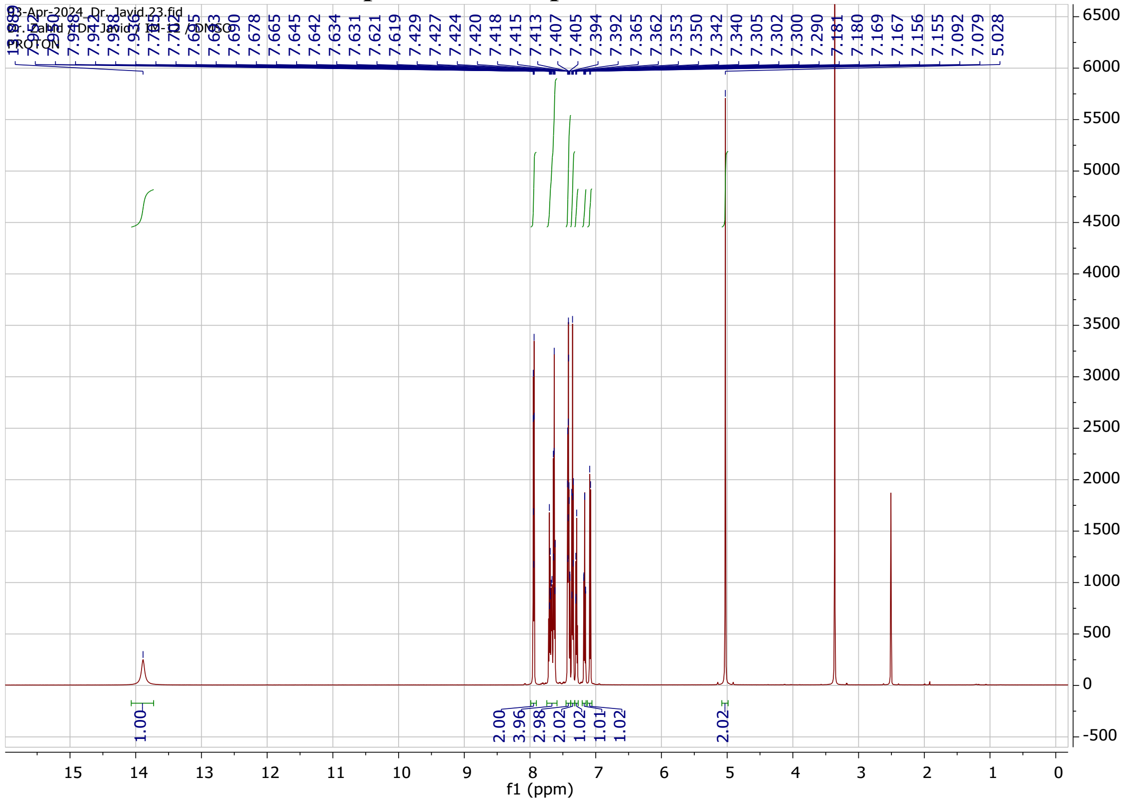

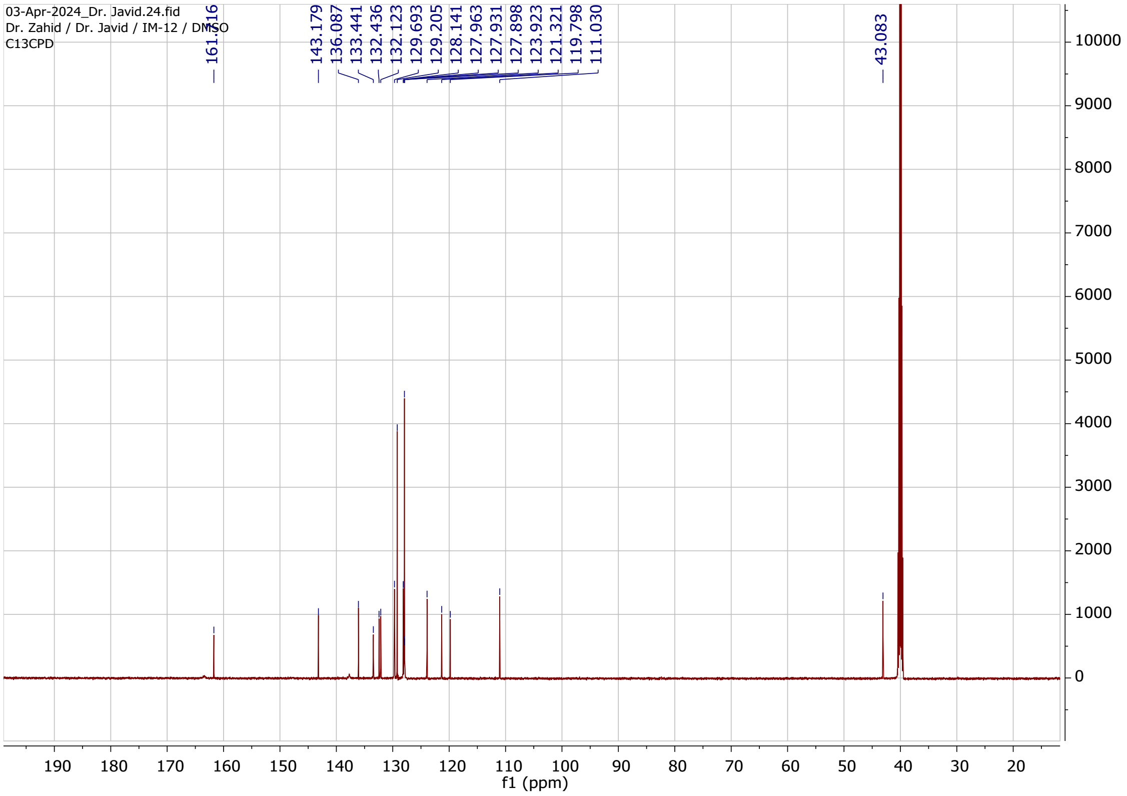

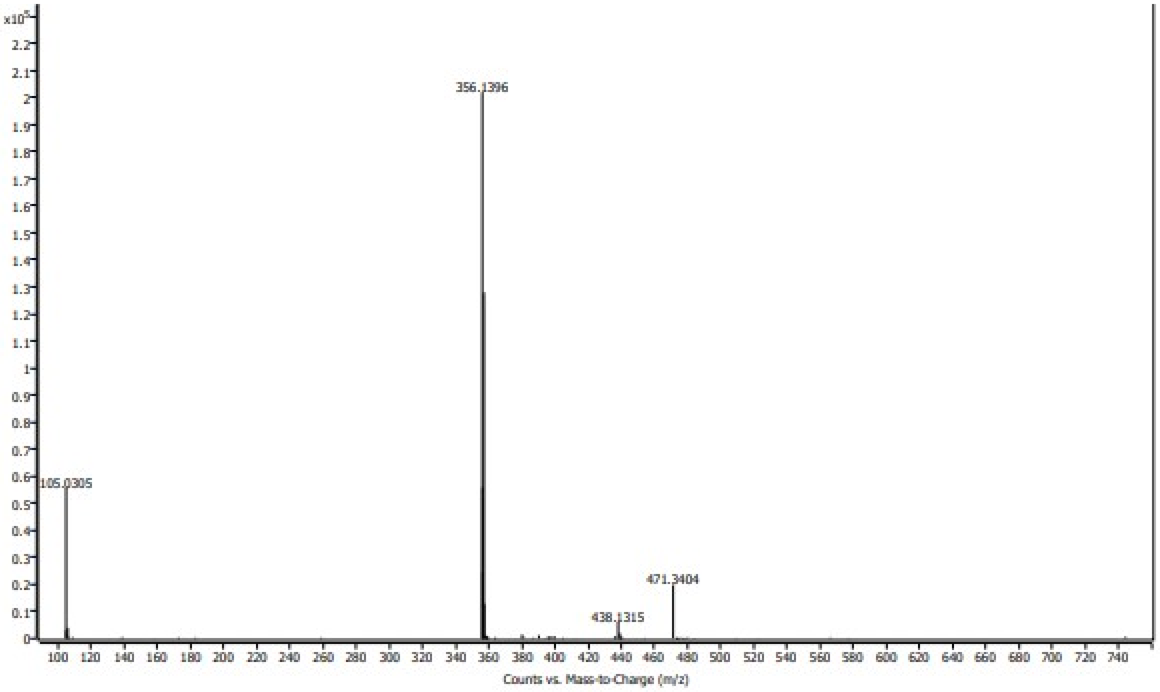

x10^5
356.1396
105.0305
471.3404
438.1315
Counts vs. Mass-to-Charge (m/z)

^{1}HNMR, ^{13}CNMR, and mass spectra of compound 12

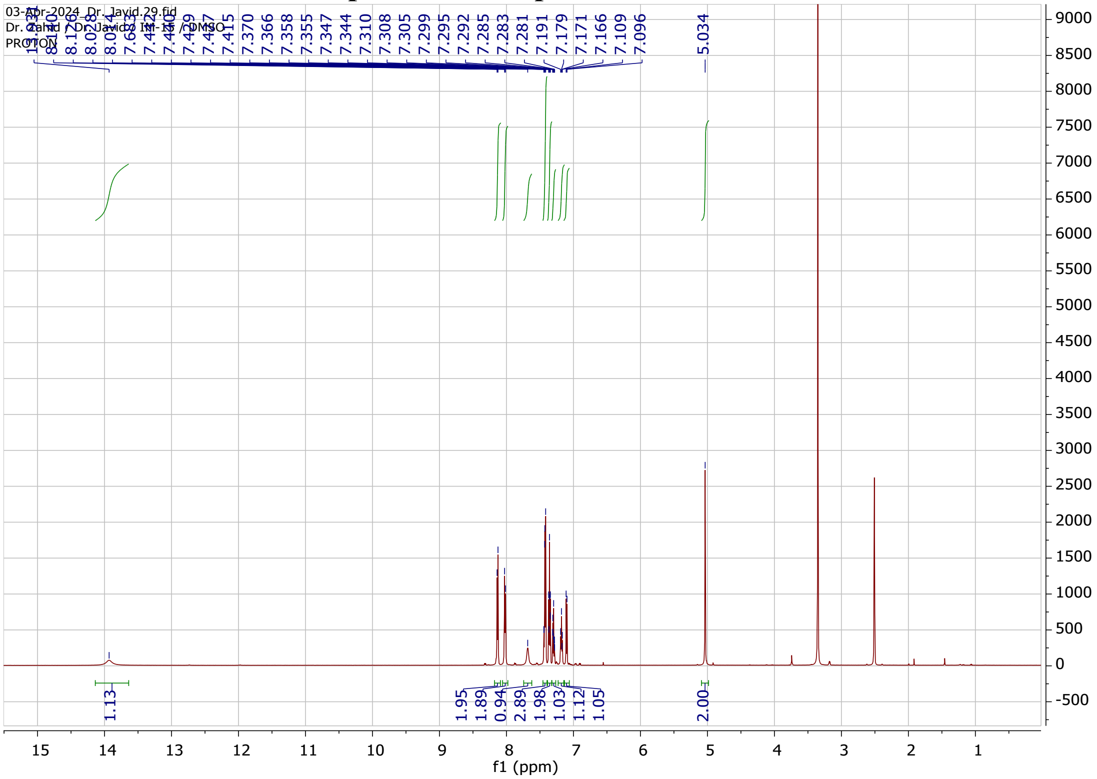

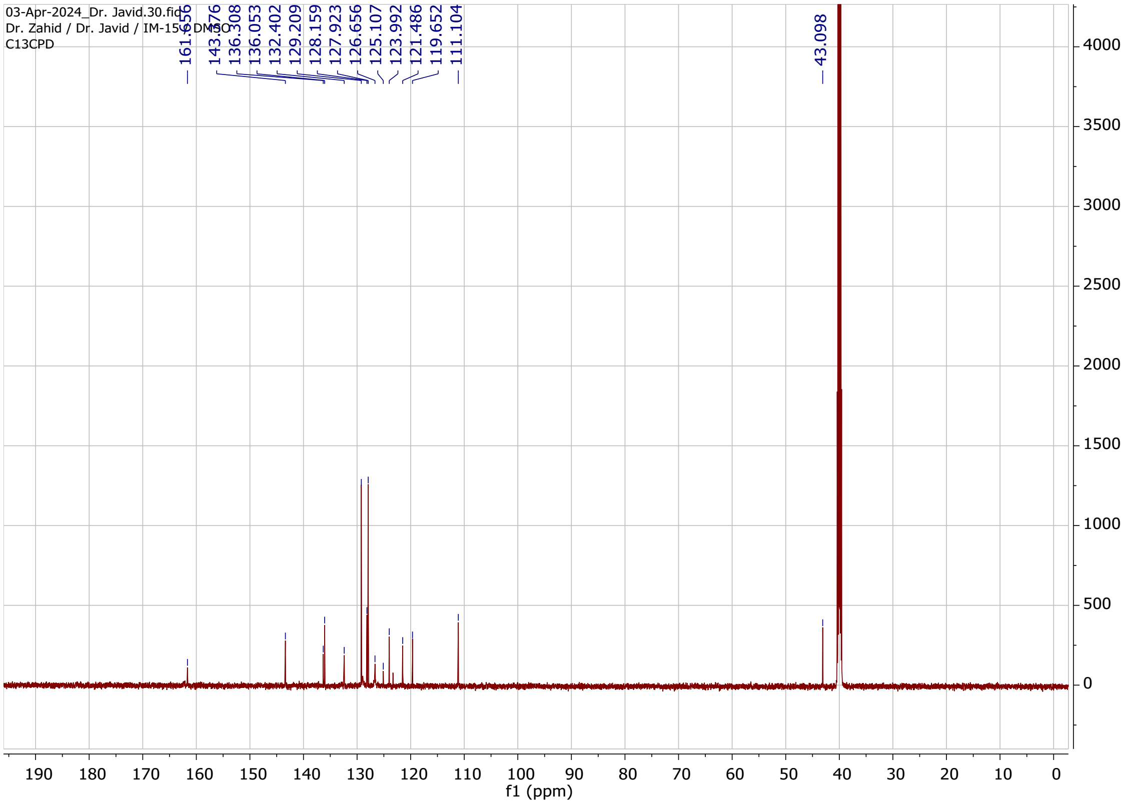

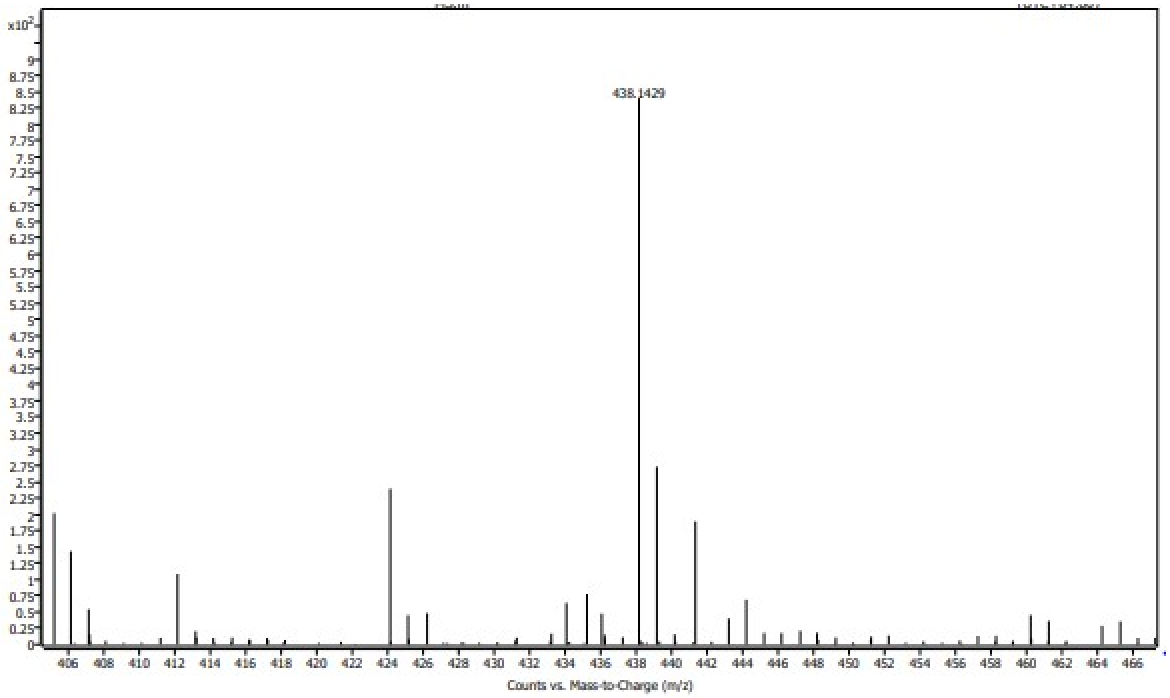

x10^2
438.1429
Counts vs. Mass-to-Charge (m/z)

^{1}HNMR, ^{13}CNMR, and mass spectra of compound 13

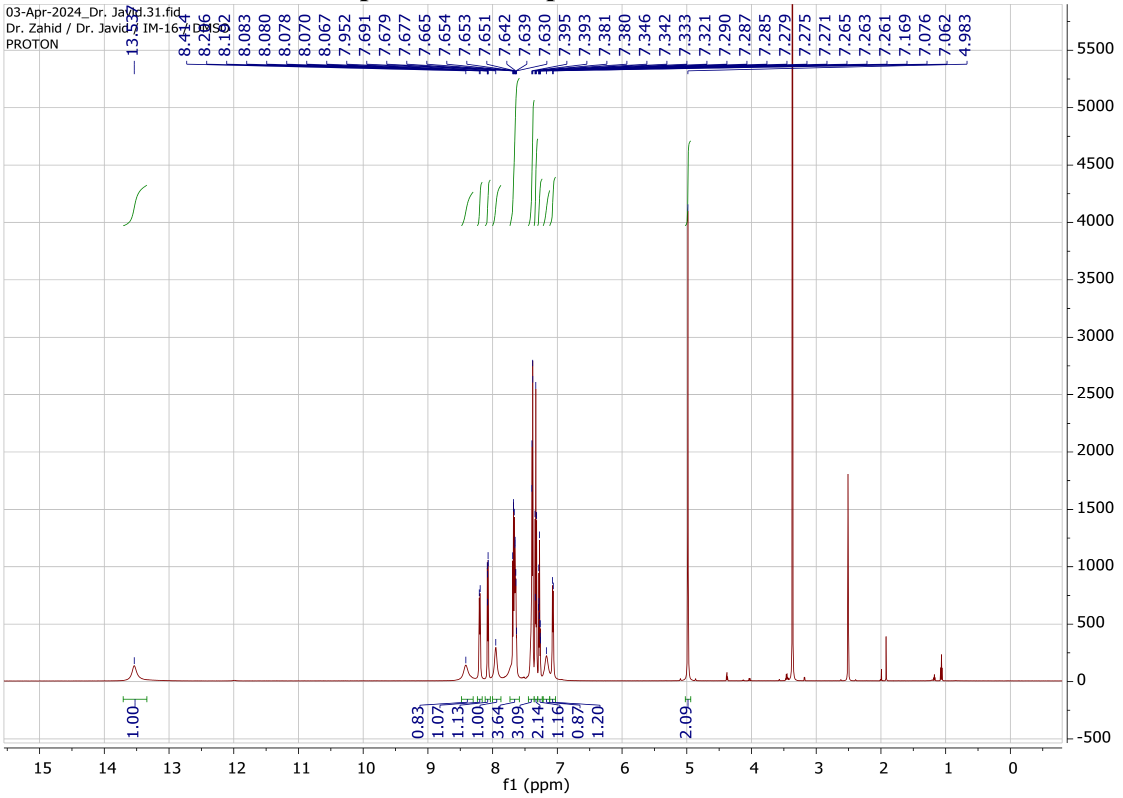

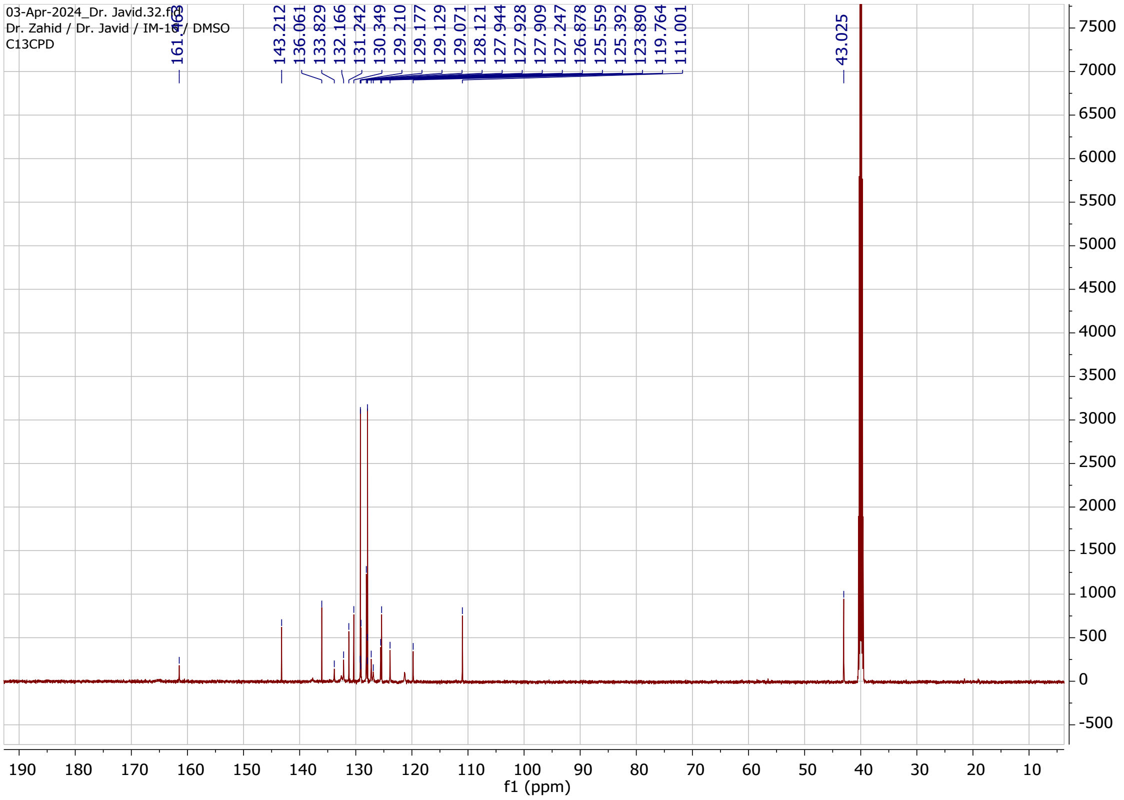

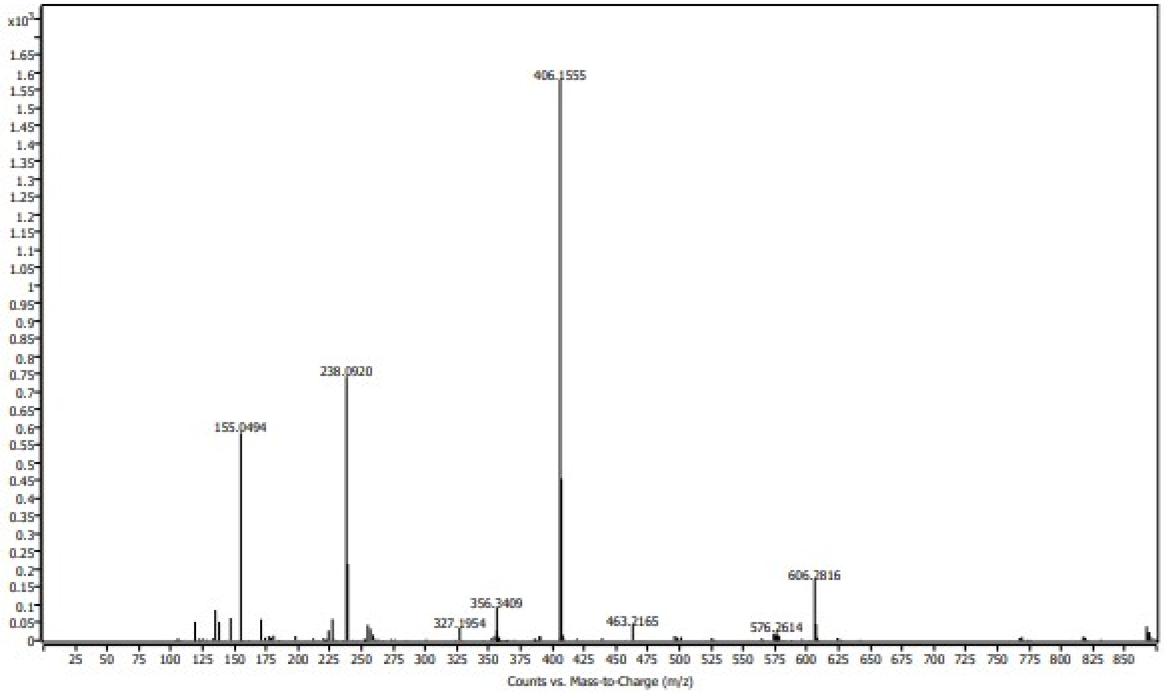

x10^7
406.1555
238.0920
155.0494
606.2816
356.3409
327.1954
463.2165
576.2614
Counts vs. Mass-to-Charge (m/z)

^{1}HNMR, ^{13}CNMR, and mass spectra of compound 14

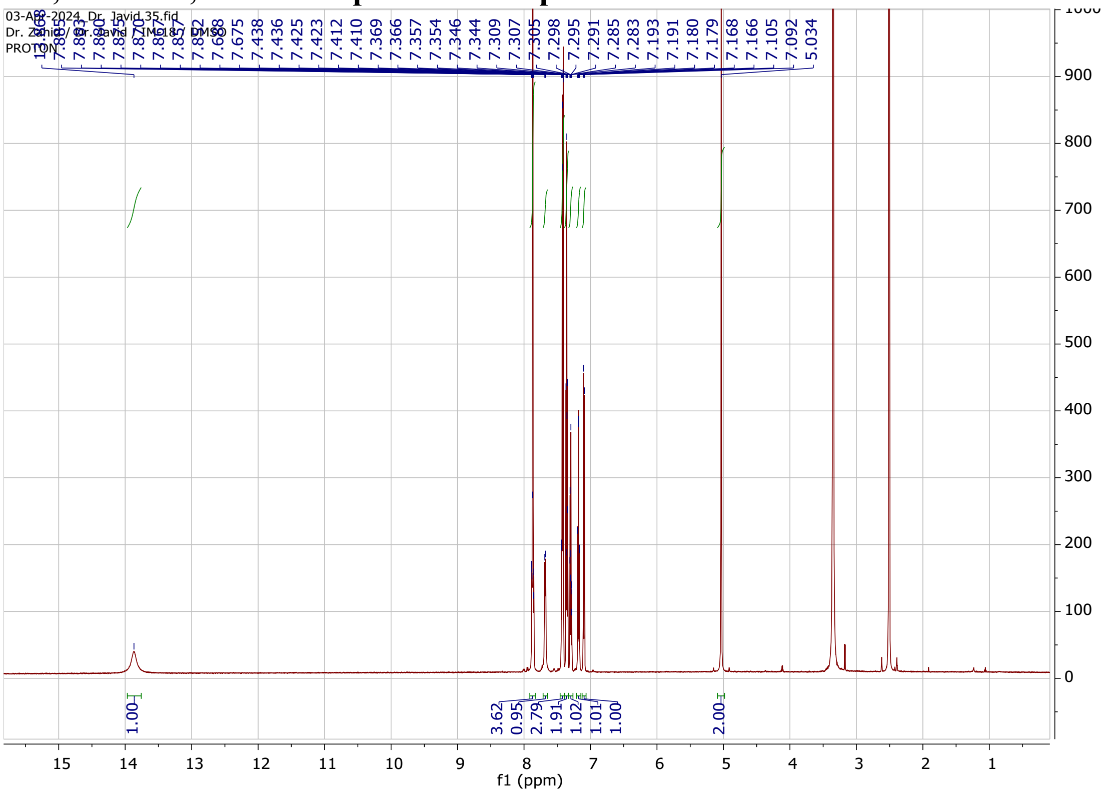

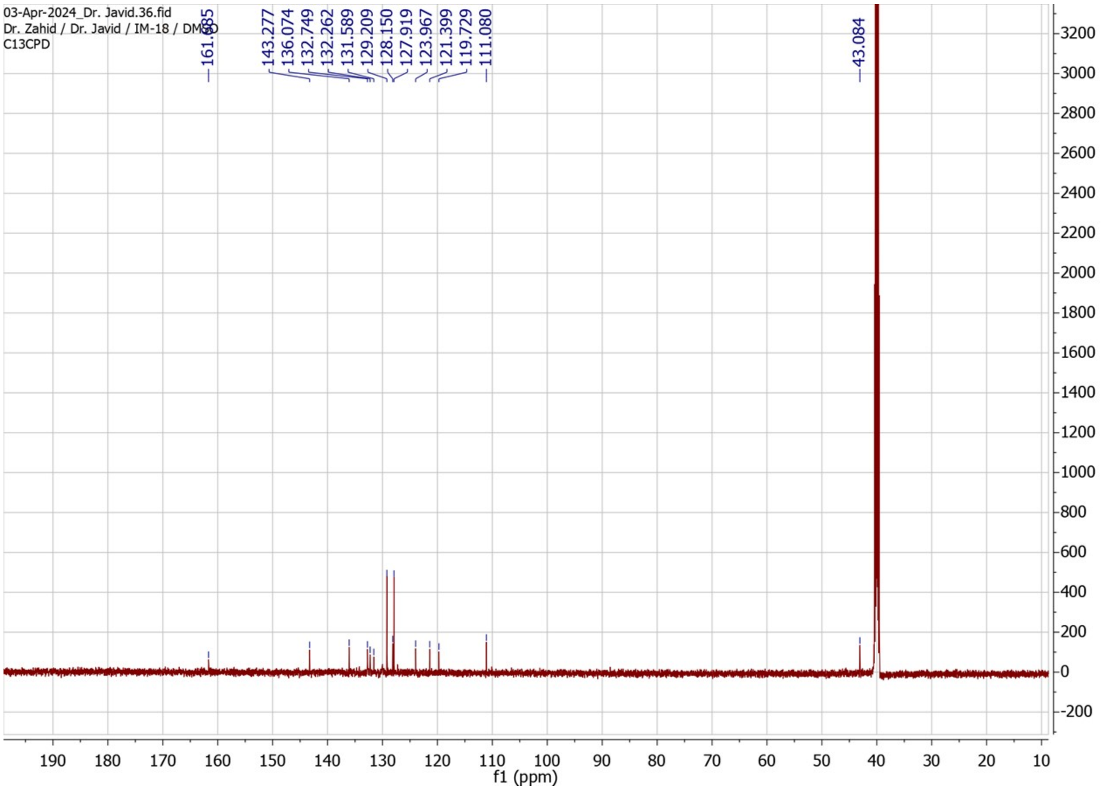

Sample Name	IM-18	Rack Position		Instrument	Instrument 1	Acq Operator	
Inj Vol (ul)	3	Plate Position		IRM Status	All ions missed		
Data File	IM-18.d	Acq Method	APCI POS ION DMSO MS.m	Comment		Acq Time (Local)	23-Sep-24 2:19:46 PM (UTC+04:00)

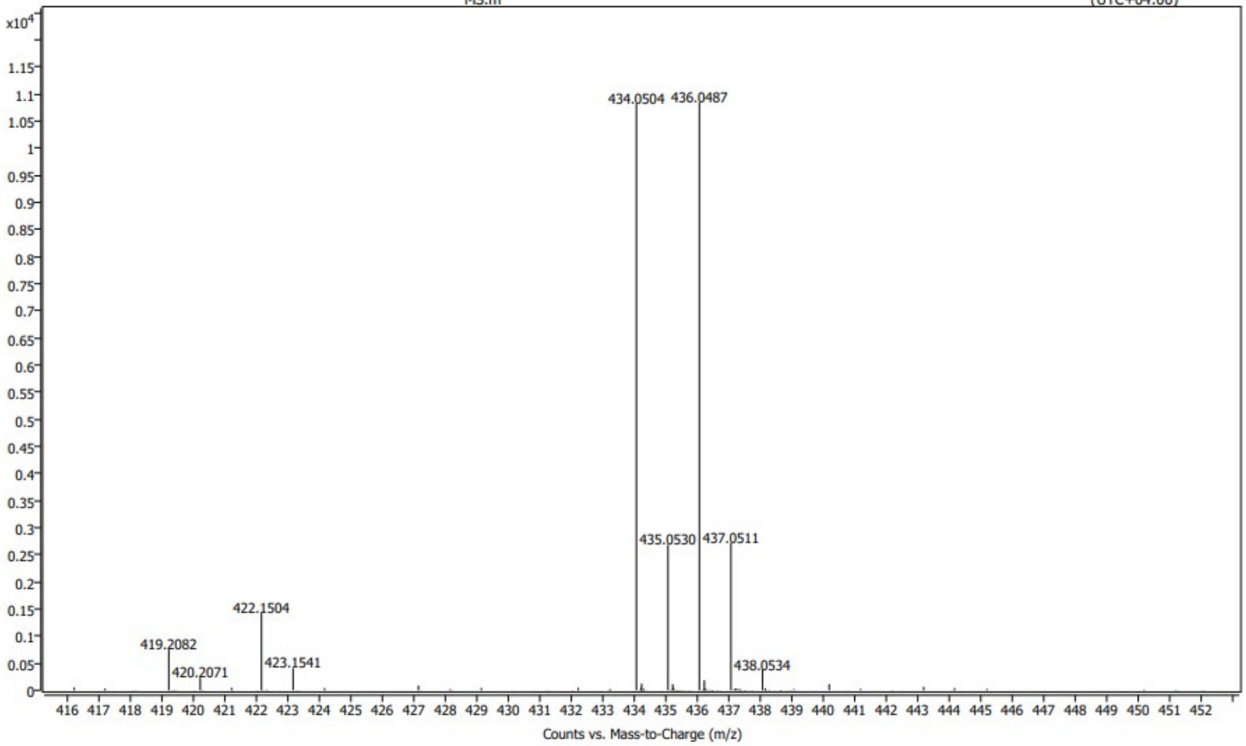

^{1}HNMR, ^{13}CNMR, and mass spectra of compound 15

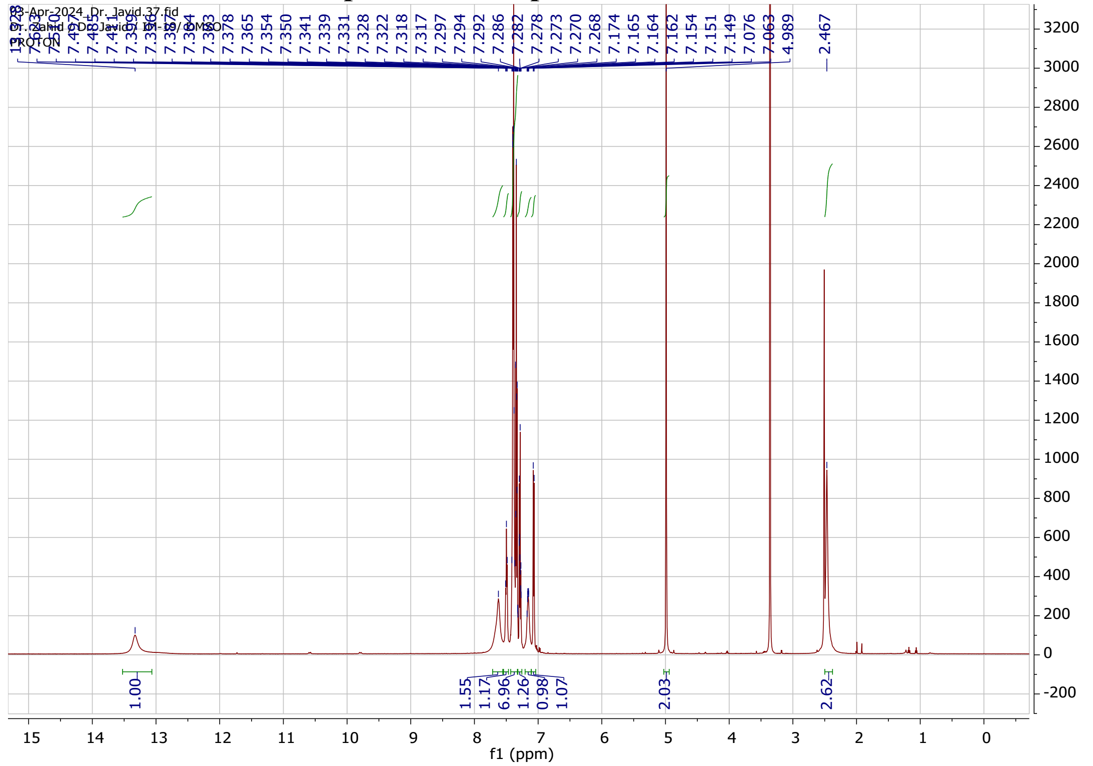

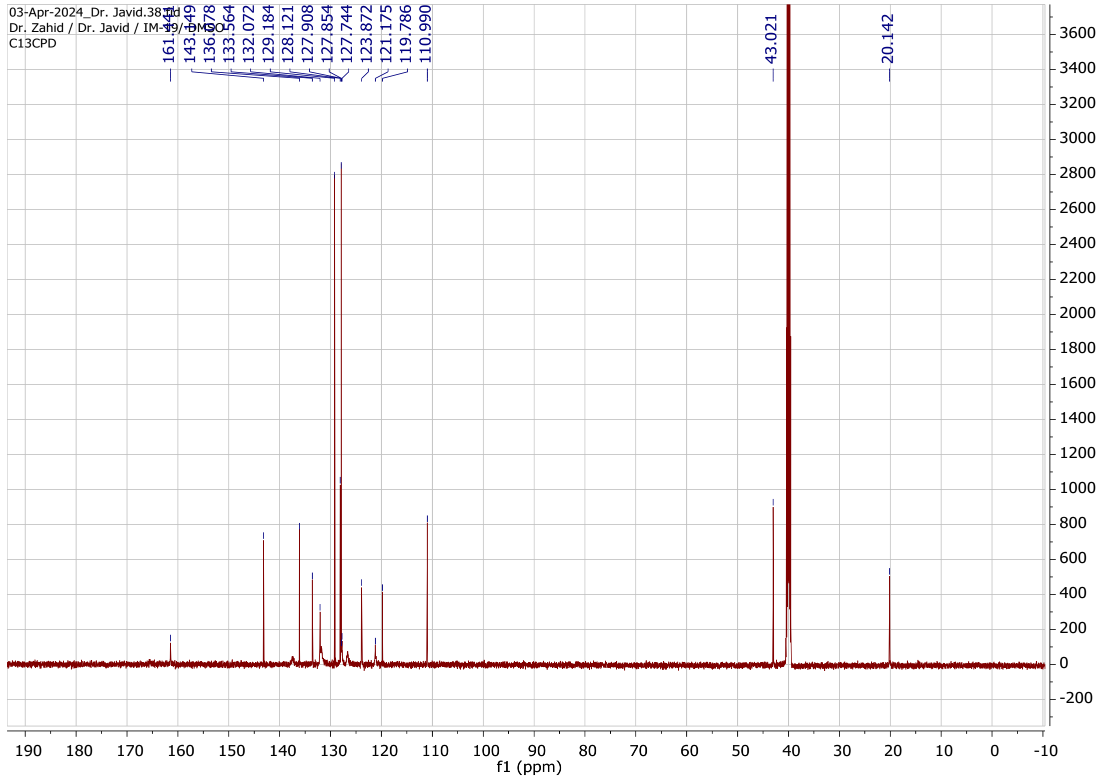

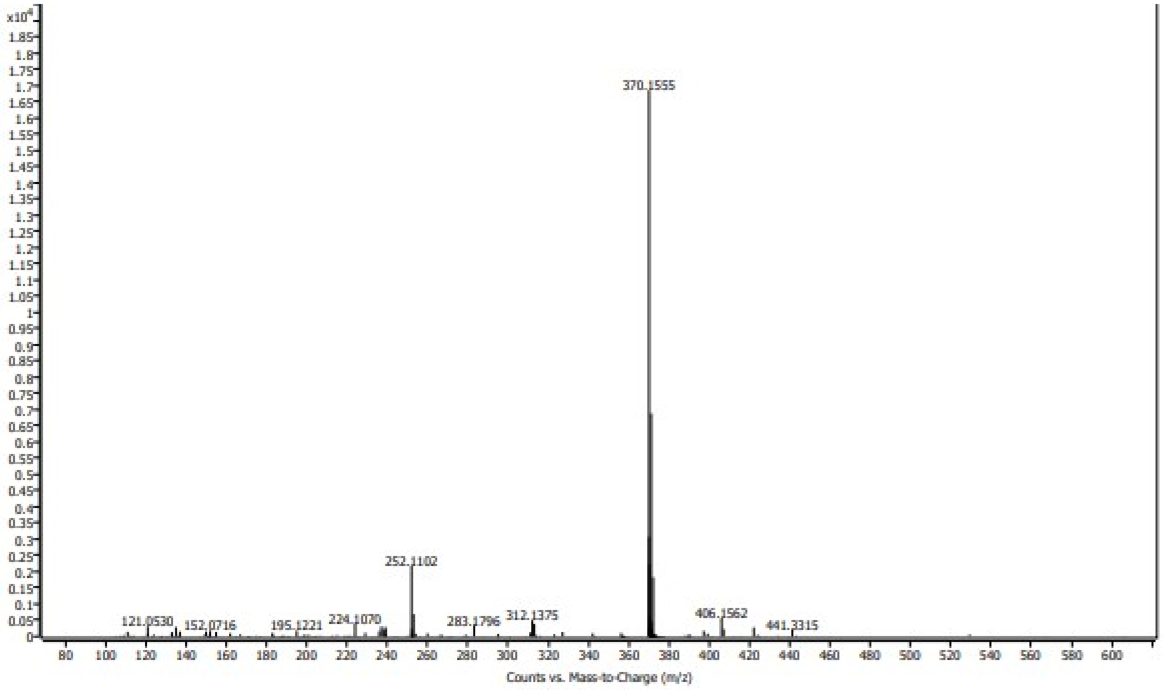

x10^4
370.1555
252.1102
121.0530
152.0716
195.1221
224.1070
283.1796
312.1375
406.1562
441.3315
Counts vs. Mass-to-Charge (m/z)

^{1}HNMR, ^{13}CNMR, and mass spectra of compound 16

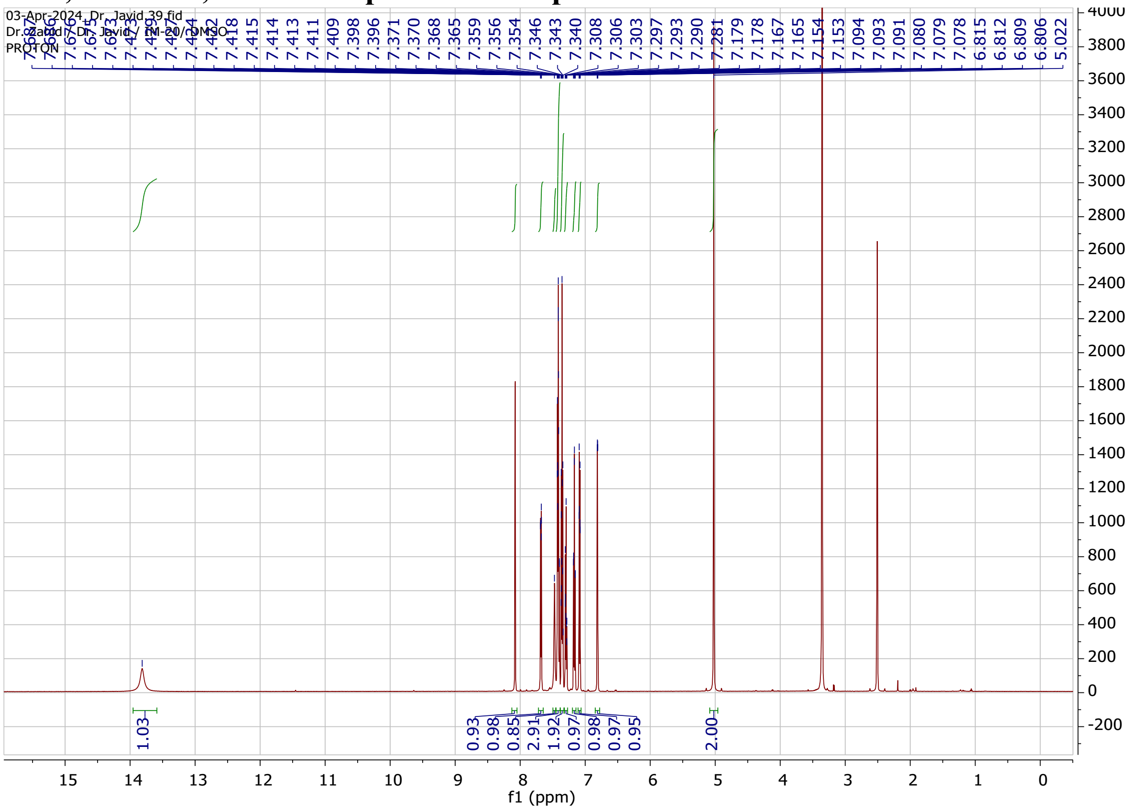

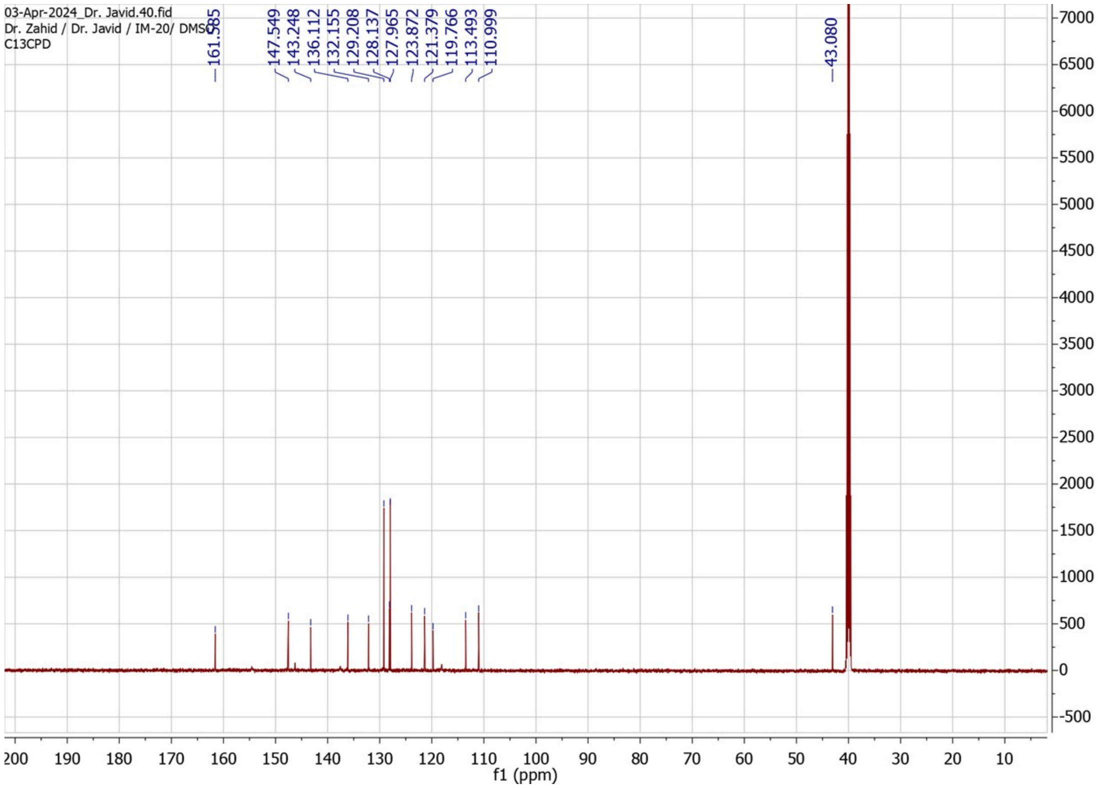

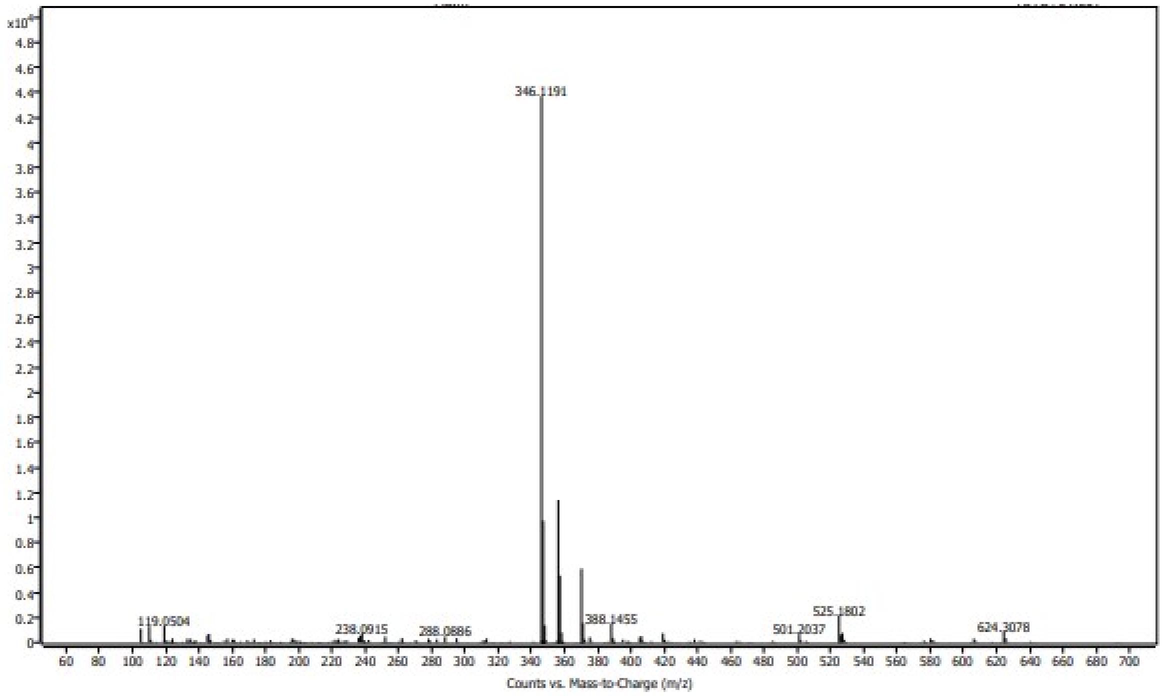

x10^4
346.1191
119.0504
238.0915
288.0886
388.1455
501.2037
525.1802
624.3078
Counts vs. Mass-to-Charge (m/z)

^{1}HNMR, ^{13}CNMR, and mass spectra of compound 17

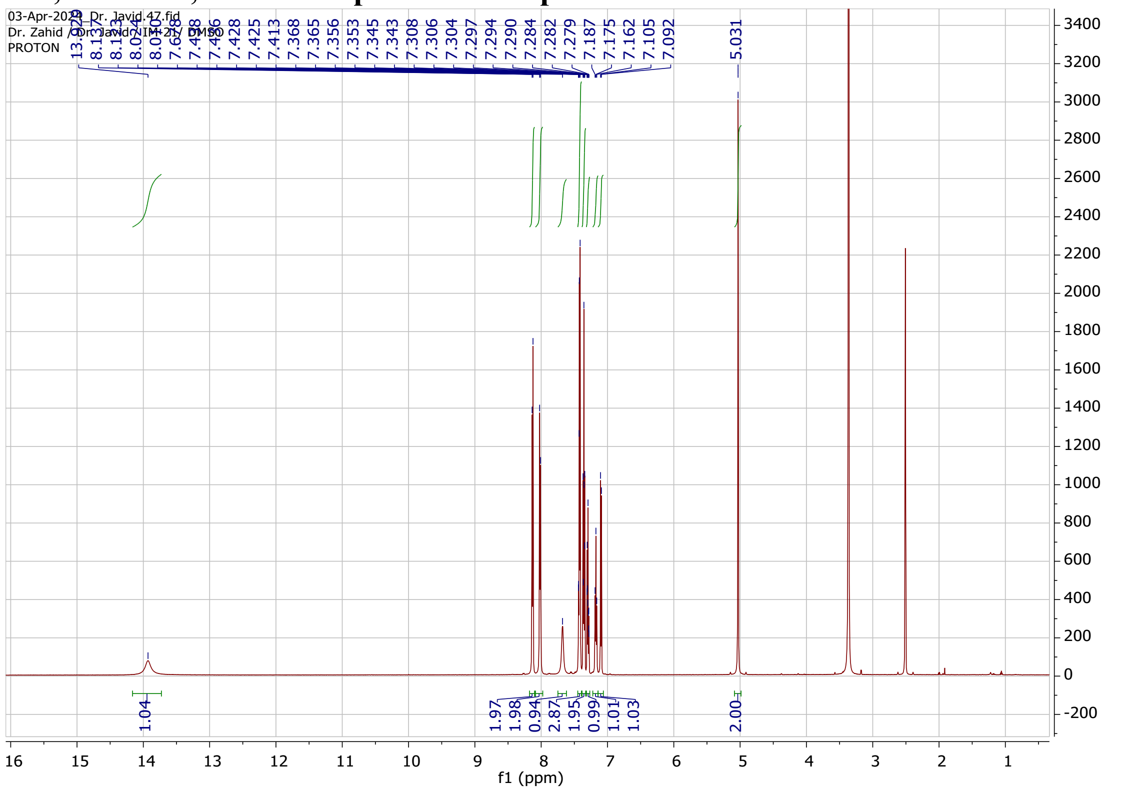

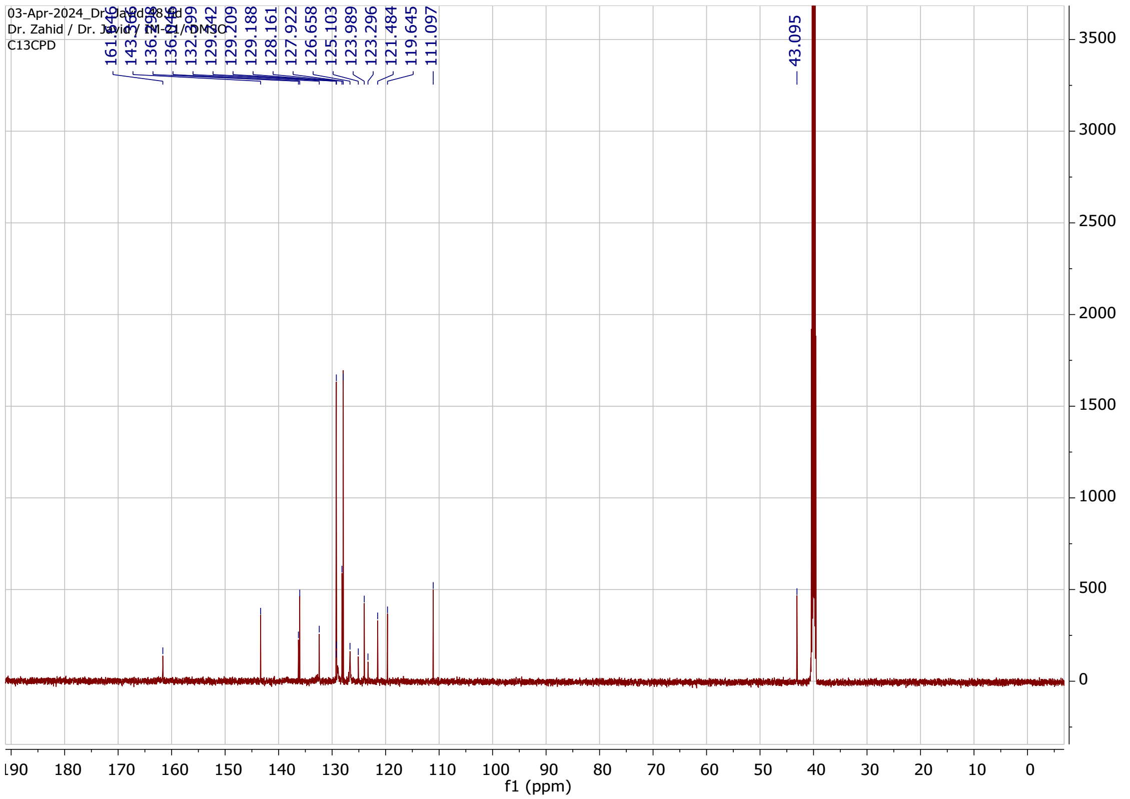

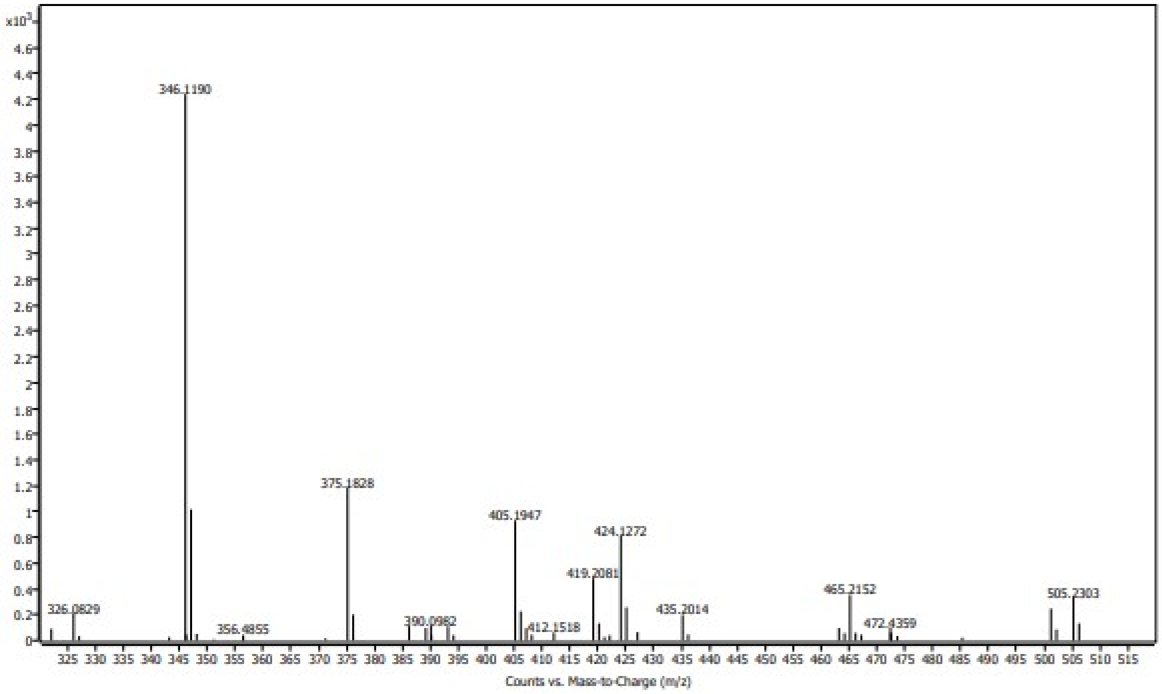

x10^2
346.1190
375.1828
405.1947
424.1272
419.2081
326.0829
356.4855
390.0982
412.1518
435.2014
465.2152
472.4359
505.2303
Counts vs. Mass-to-Charge (m/z)

^{1}HNMR, ^{13}CNMR, and mass spectra of compound 18

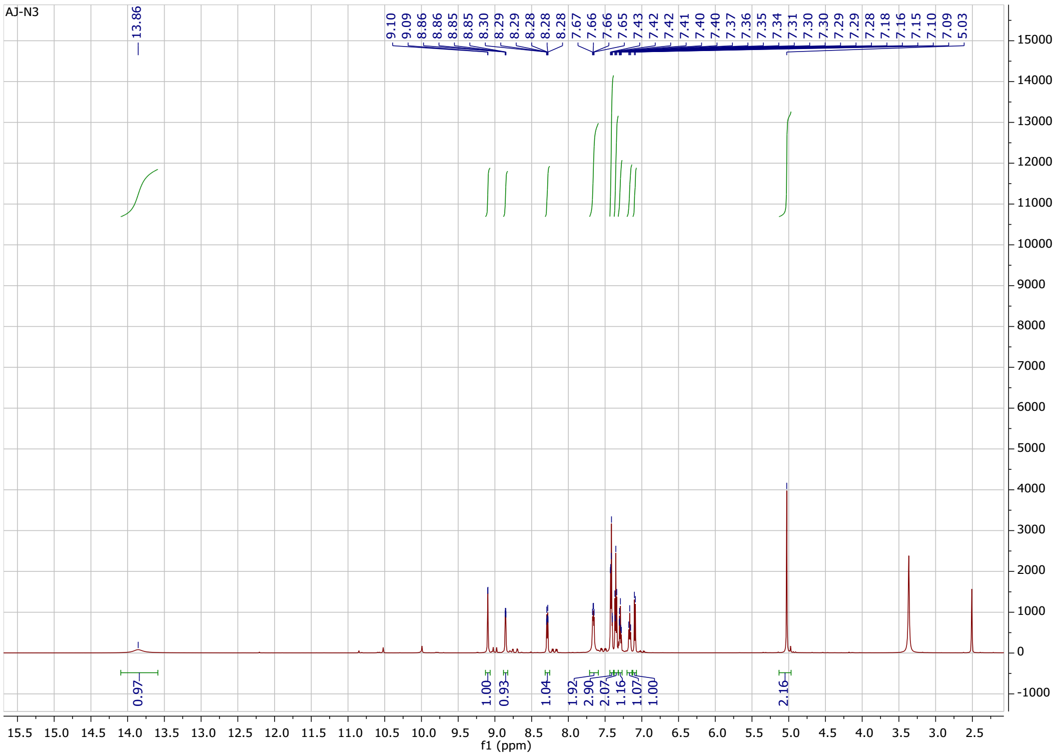

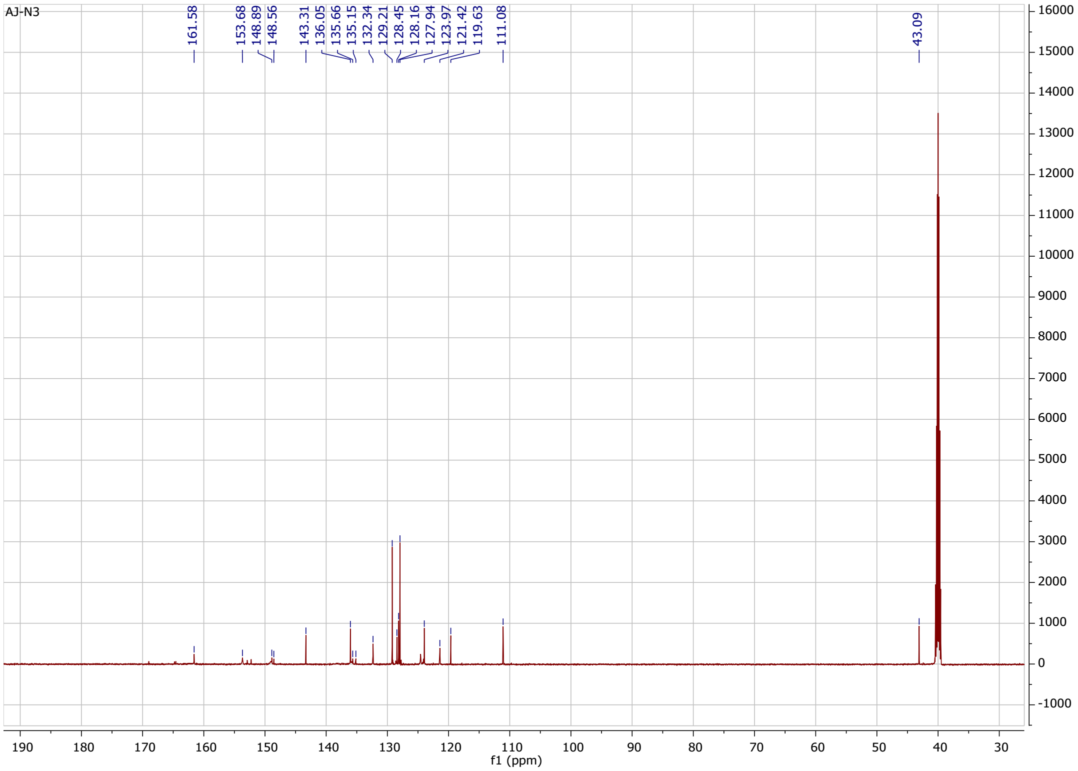

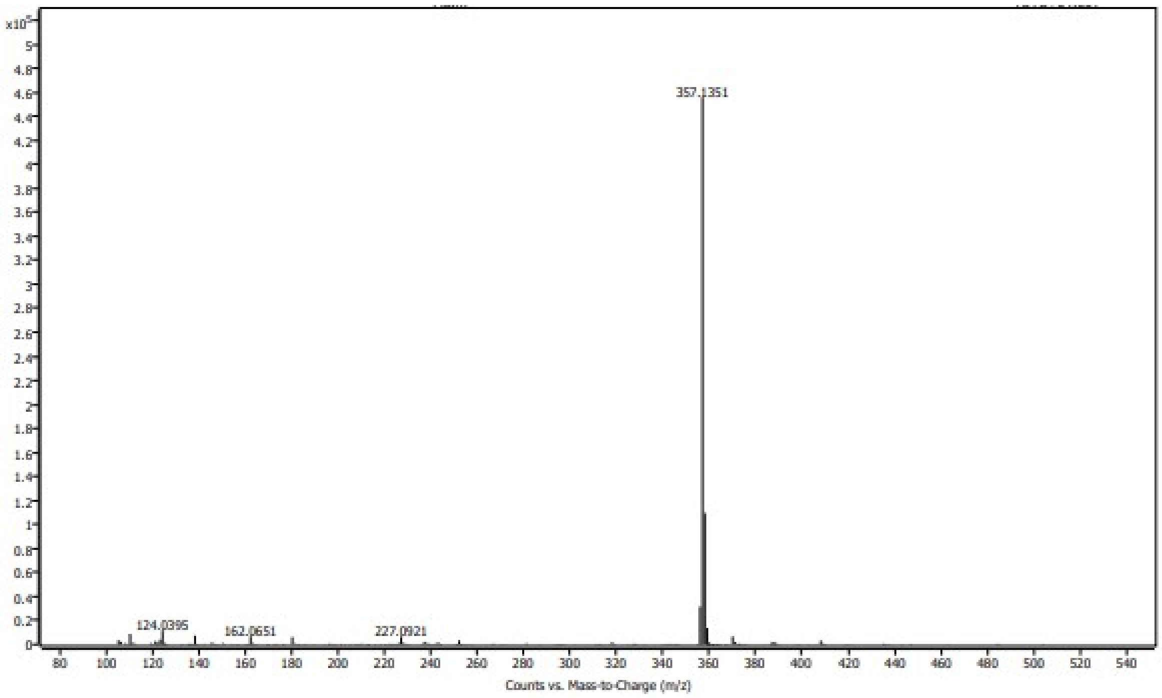
x10^5
357.1351
124.0395
162.0651
227.0921
Counts vs. Mass-to-Charge (m/z)

^{1}HNMR, ^{13}CNMR, and mass spectra of compound 19

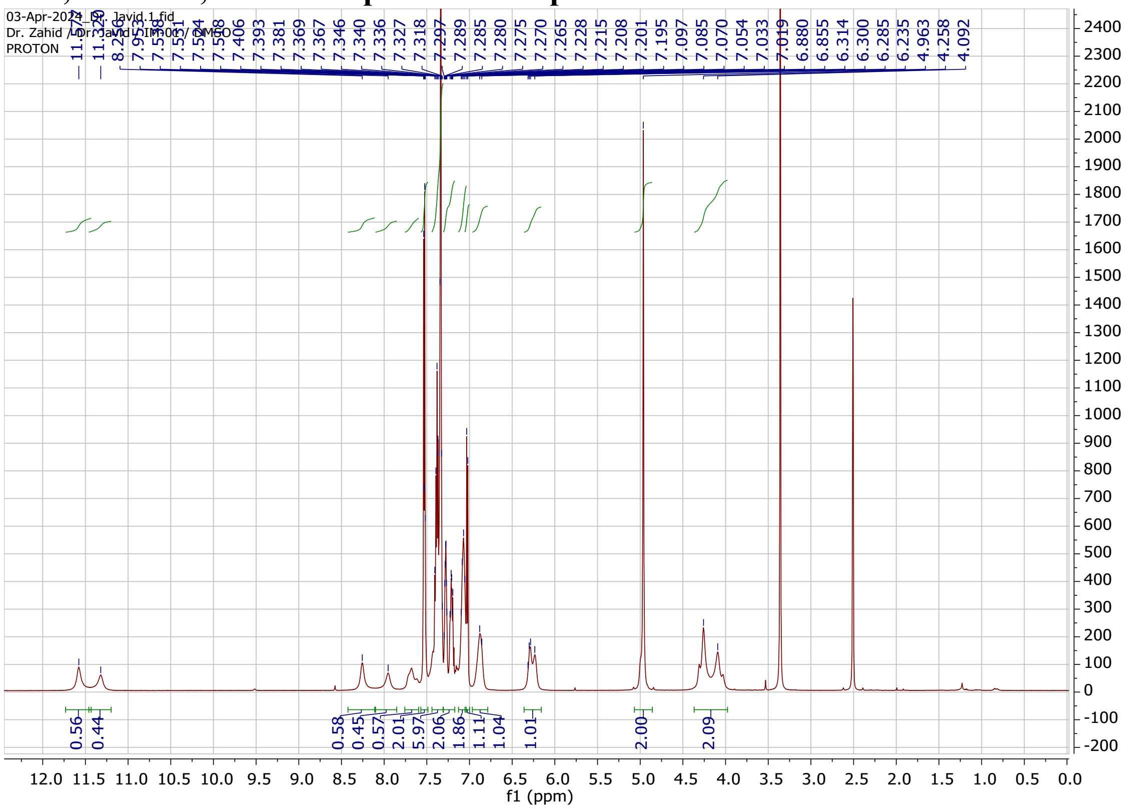

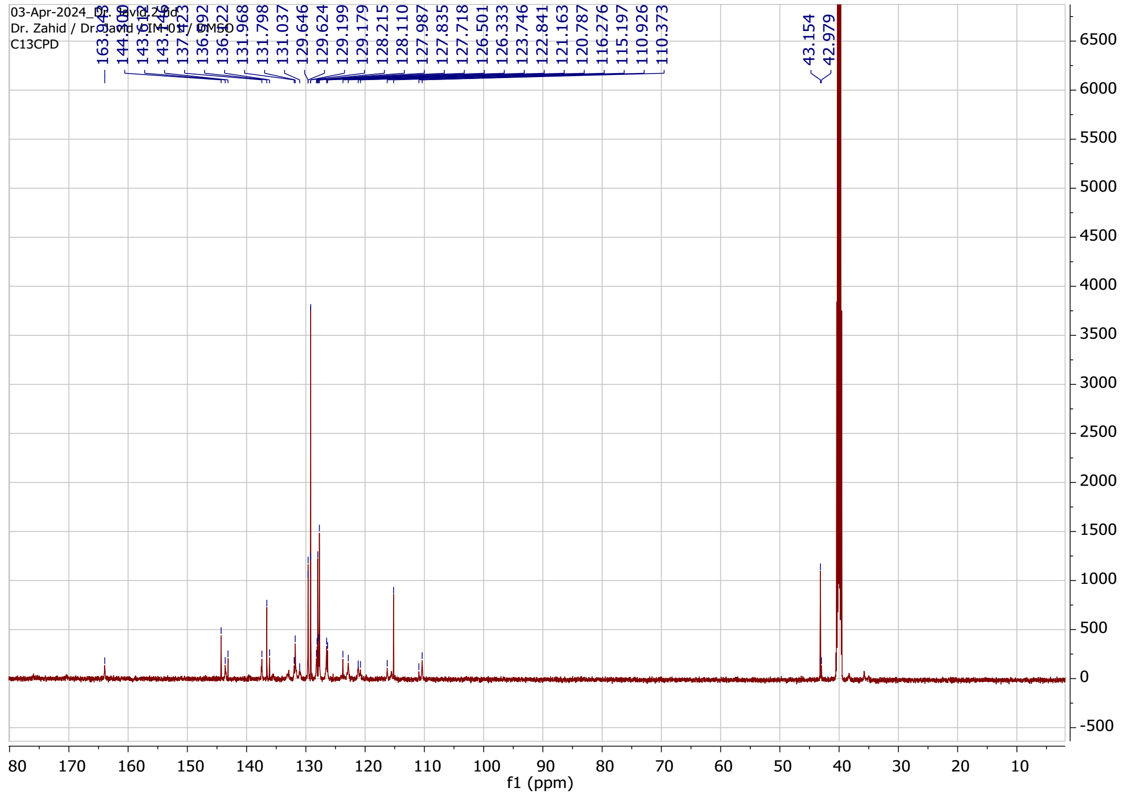

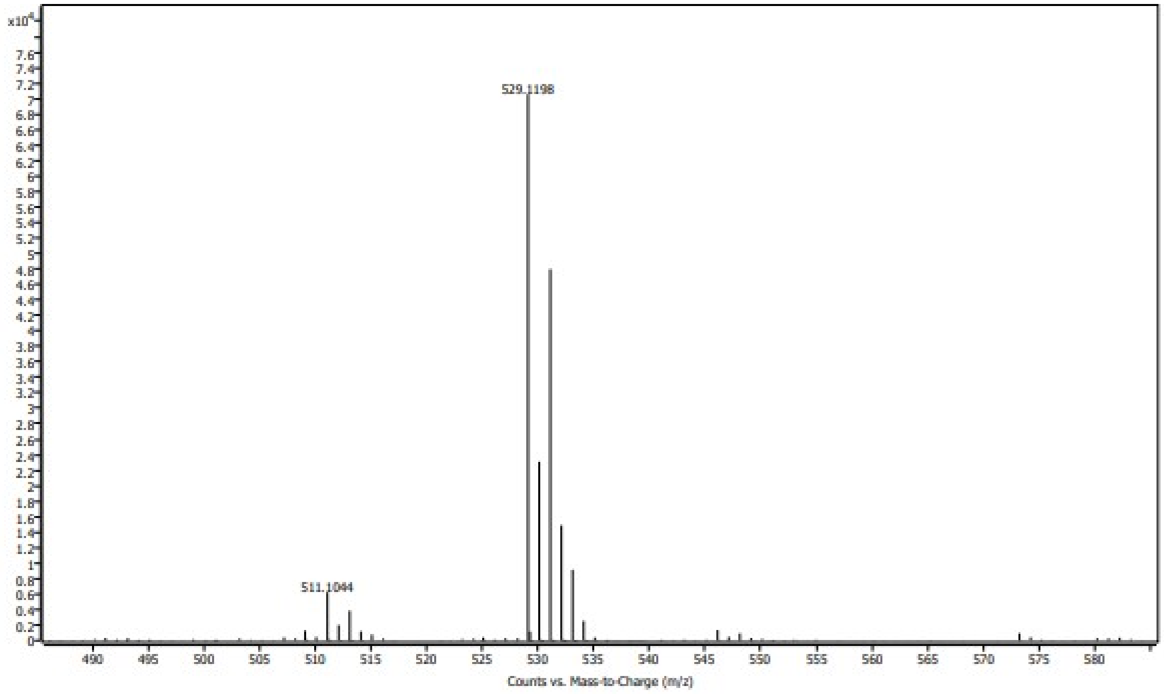

x10^4
529.1198
511.1044
Counts vs. Mass-to-Charge (m/z)

^{1}HNMR, ^{13}CNMR, and mass spectra of compound 20

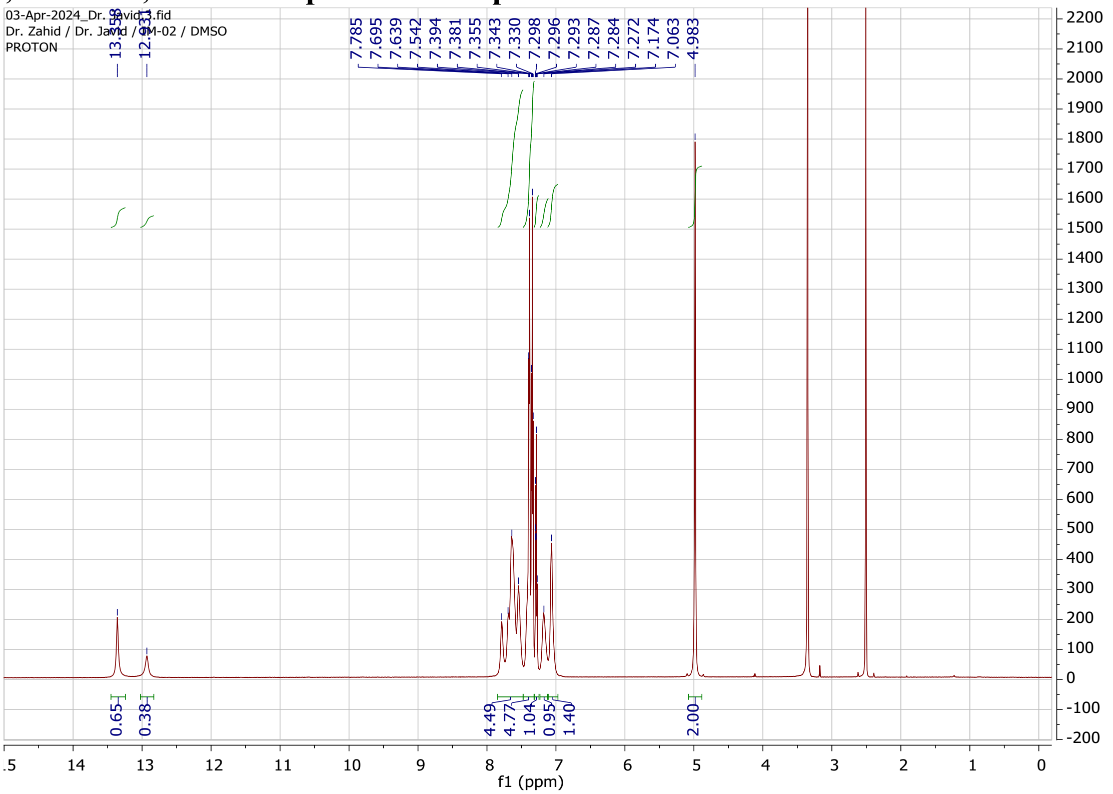

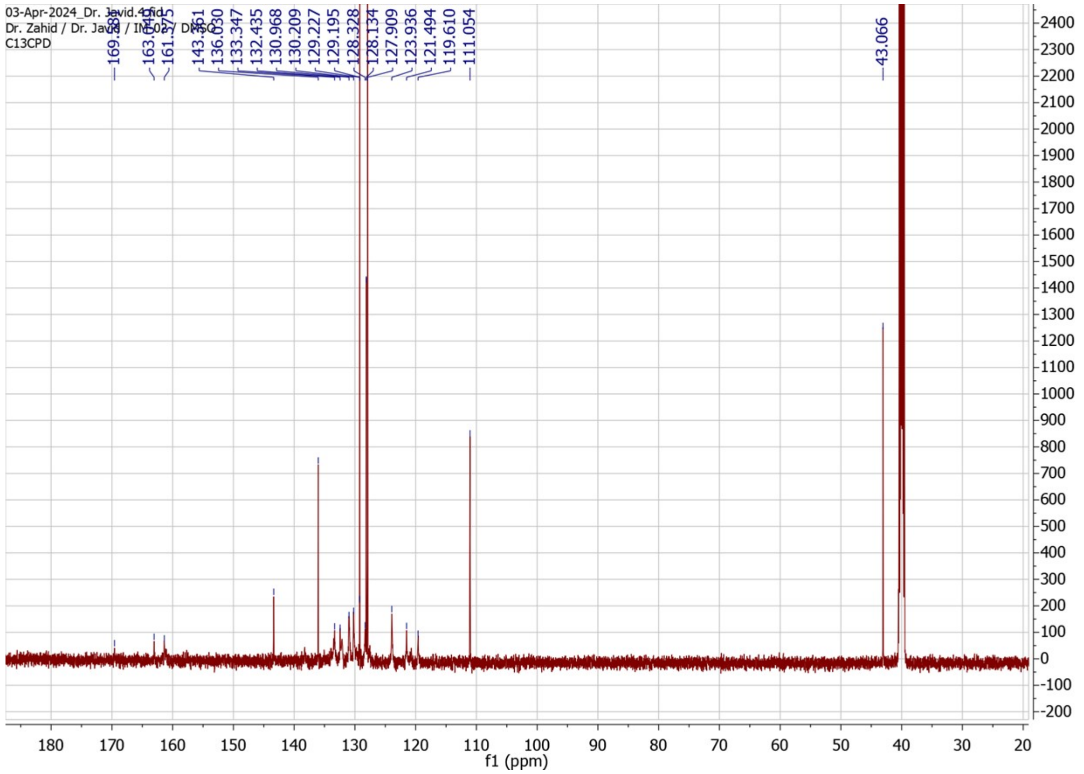

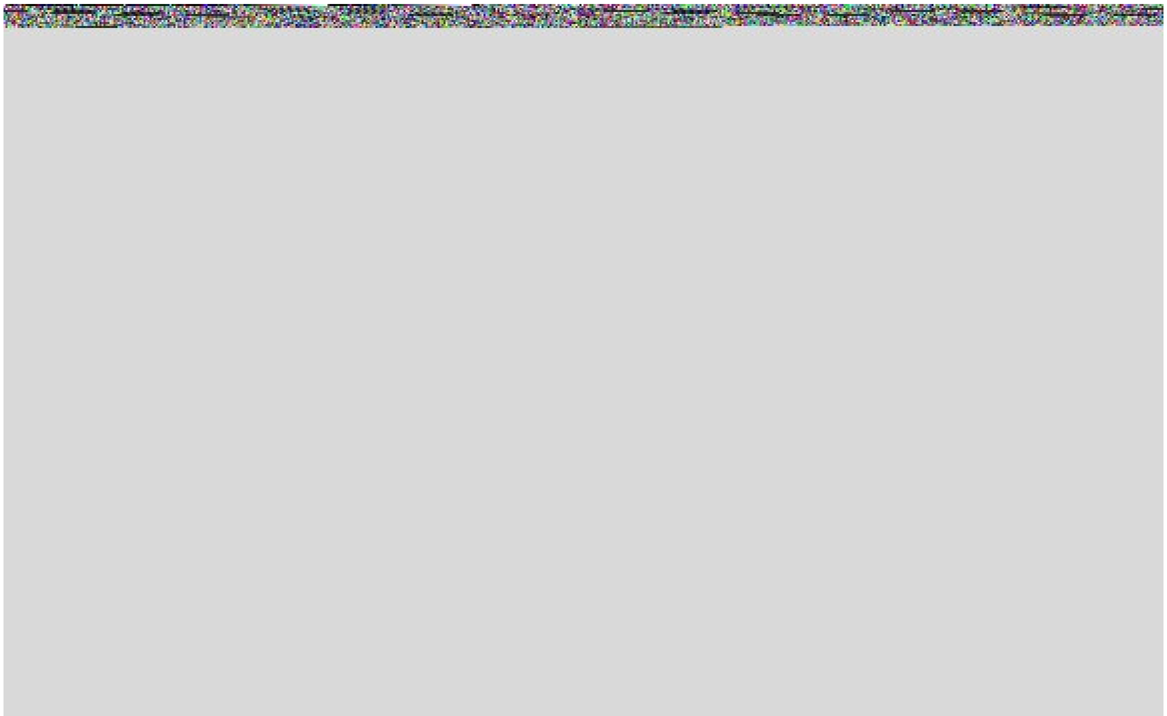

^{1}HNMR, ^{13}CNMR, and mass spectra of compound 21

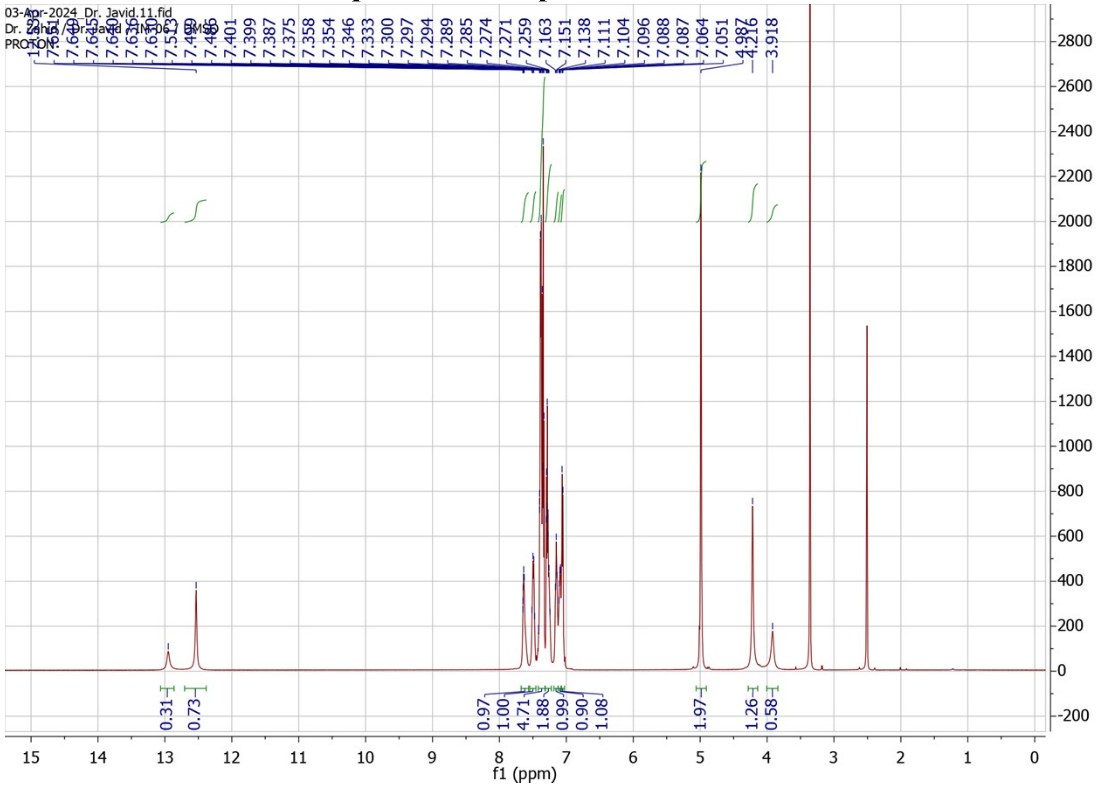

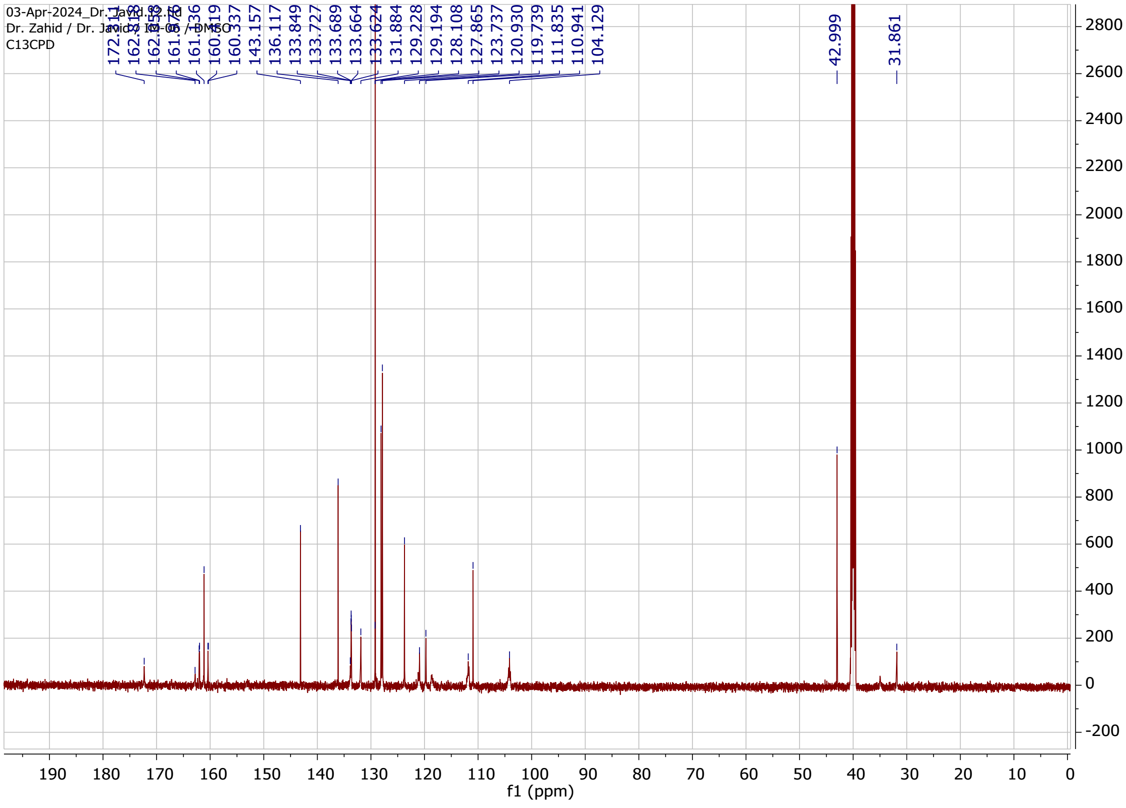

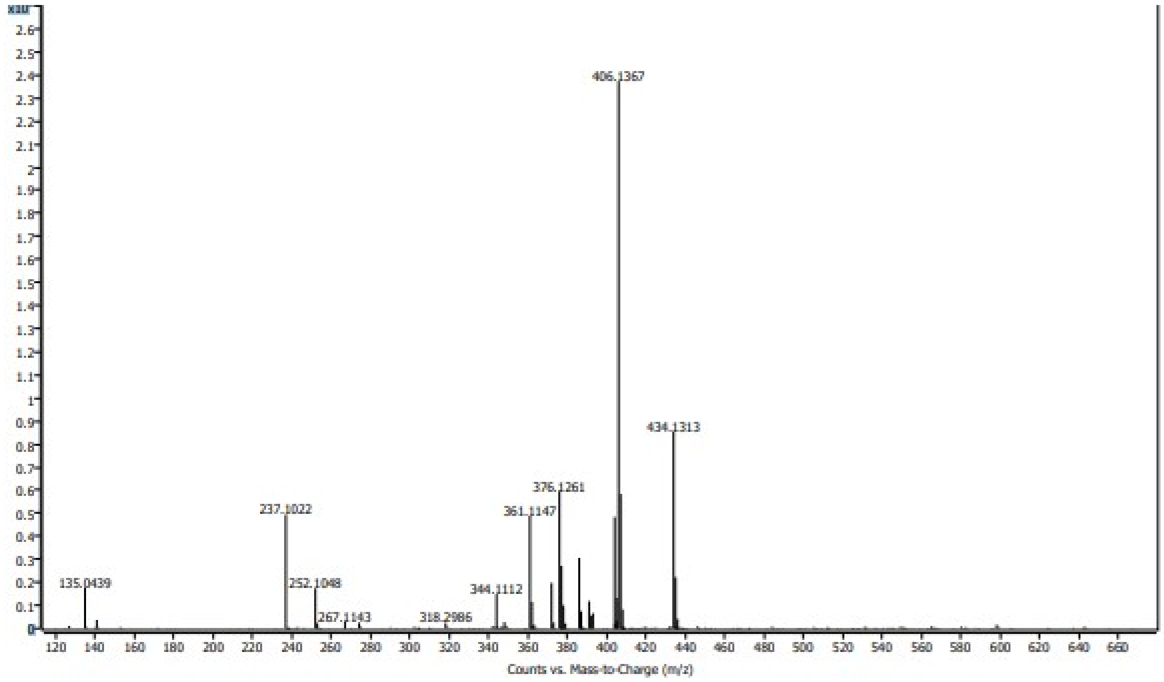

406.1367
434.1313
376.1261
361.1147
237.1022
344.1112
252.1048
135.0439
267.1143
318.2986
Counts vs. Mass-to-Charge (m/z)

^{1}HNMR, ^{13}CNMR, and mass spectra of compound 22

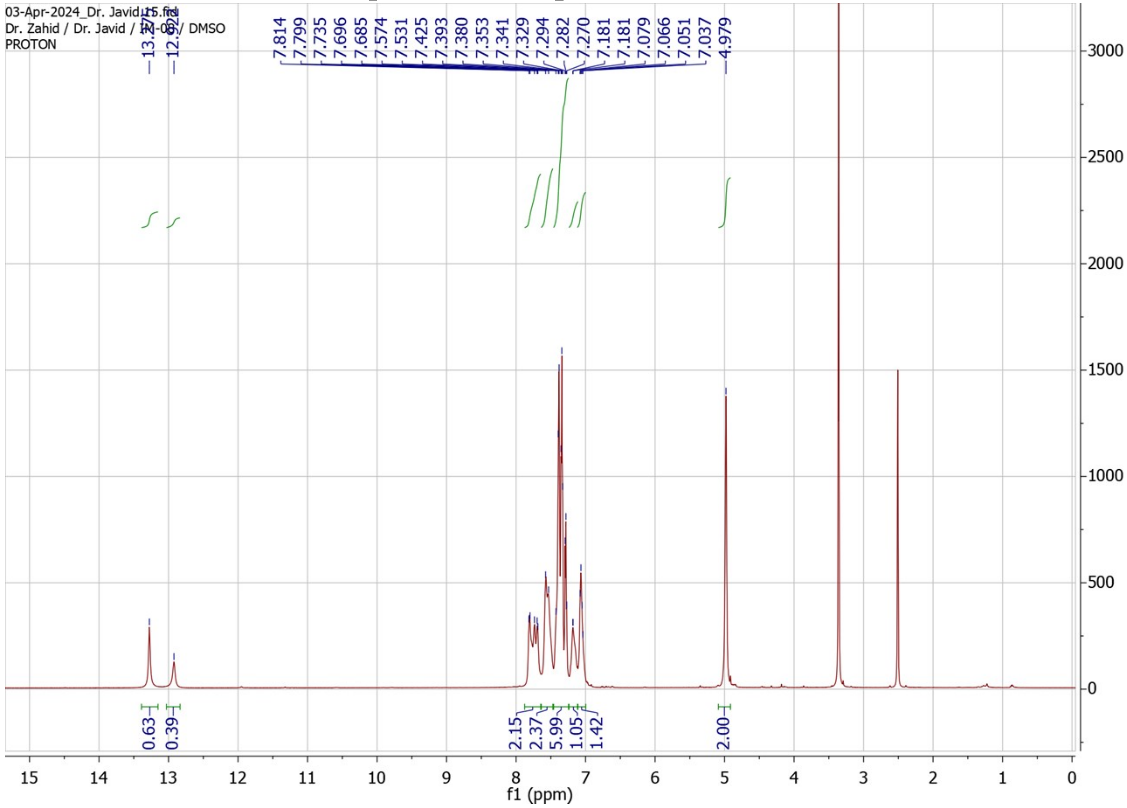

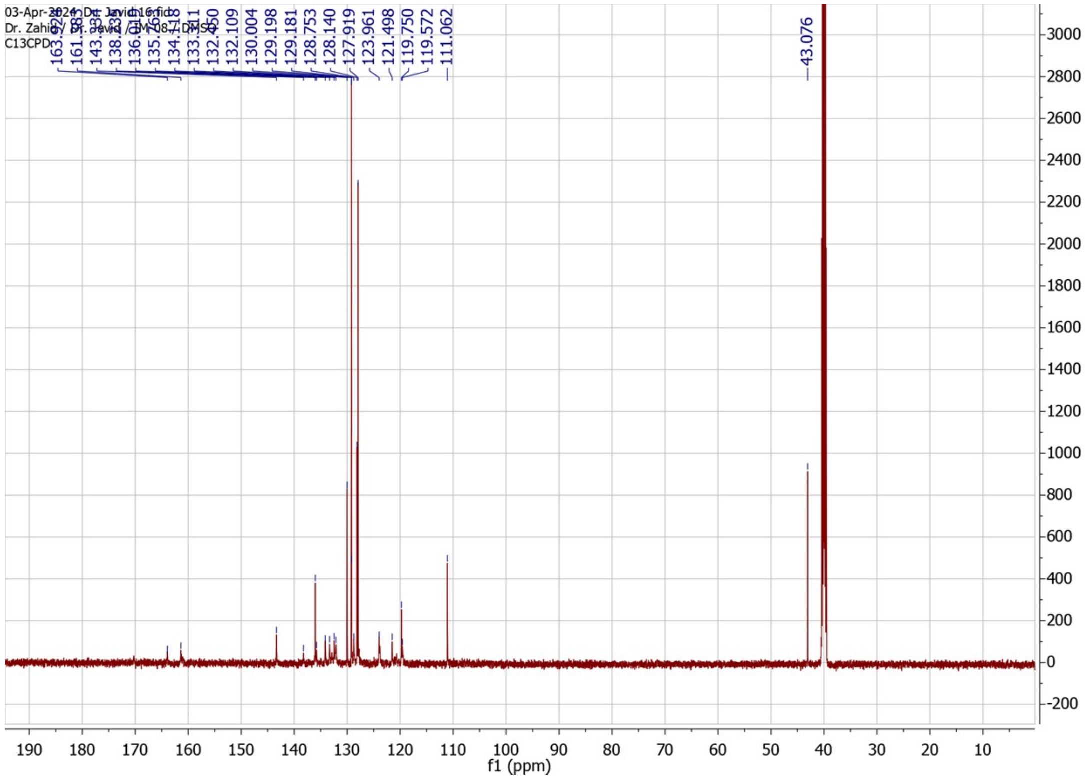

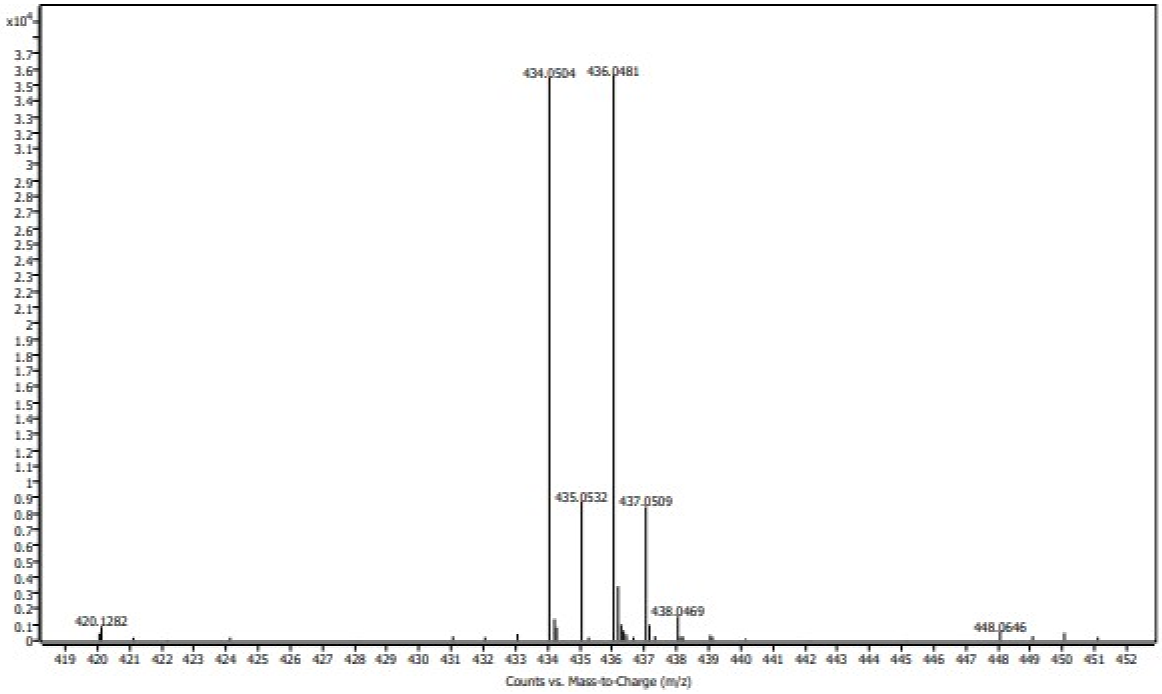
x10^4
434.0504
436.0481
435.0532
437.0509
438.0469
420.1282
448.0646
Counts vs. Mass-to-Charge (m/z)

^{1}HNMR, ^{13}CNMR, and mass spectra of compound 23

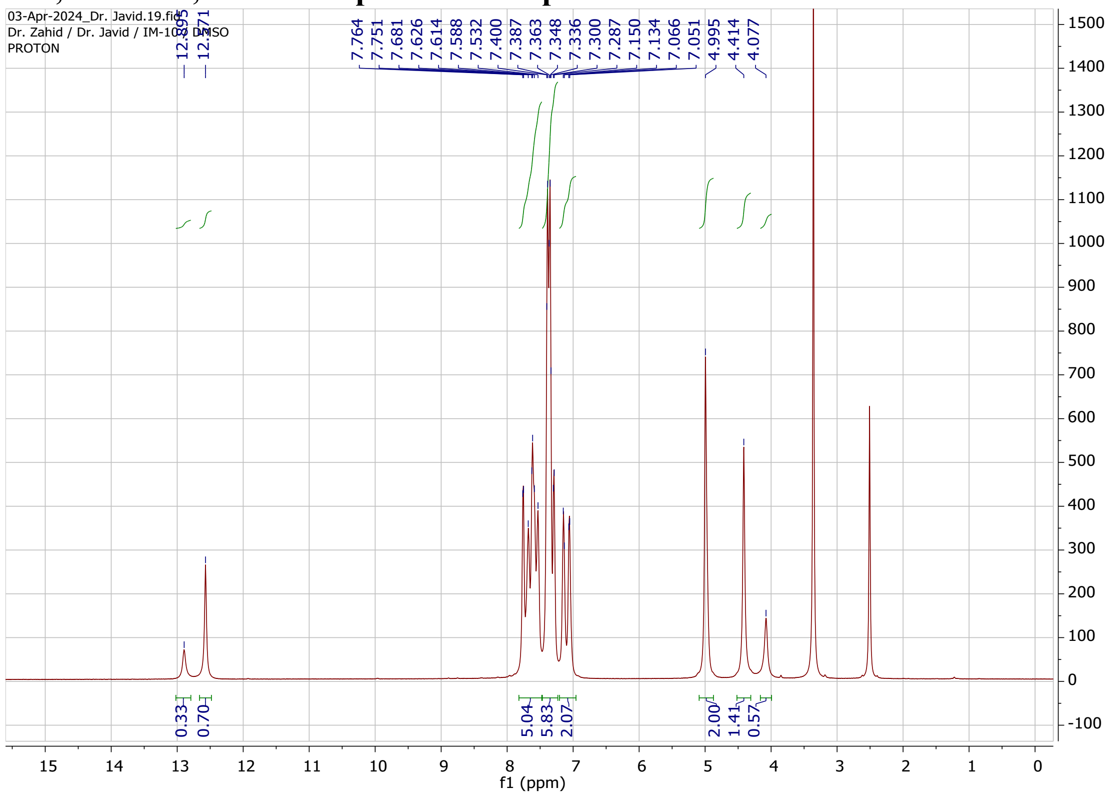

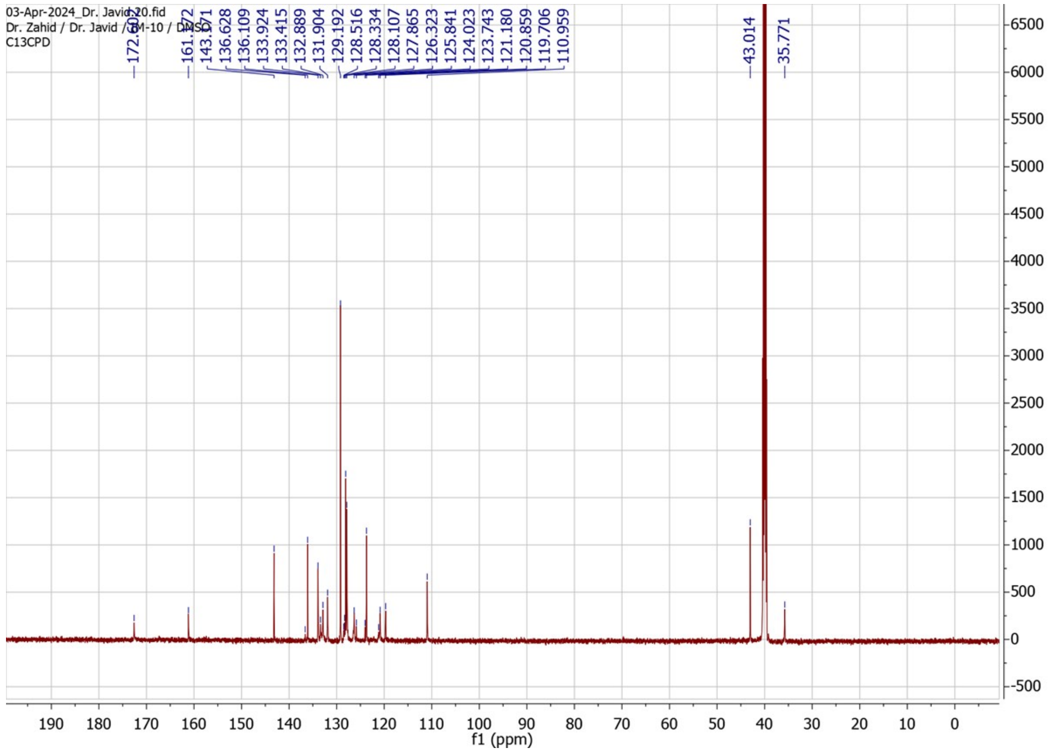

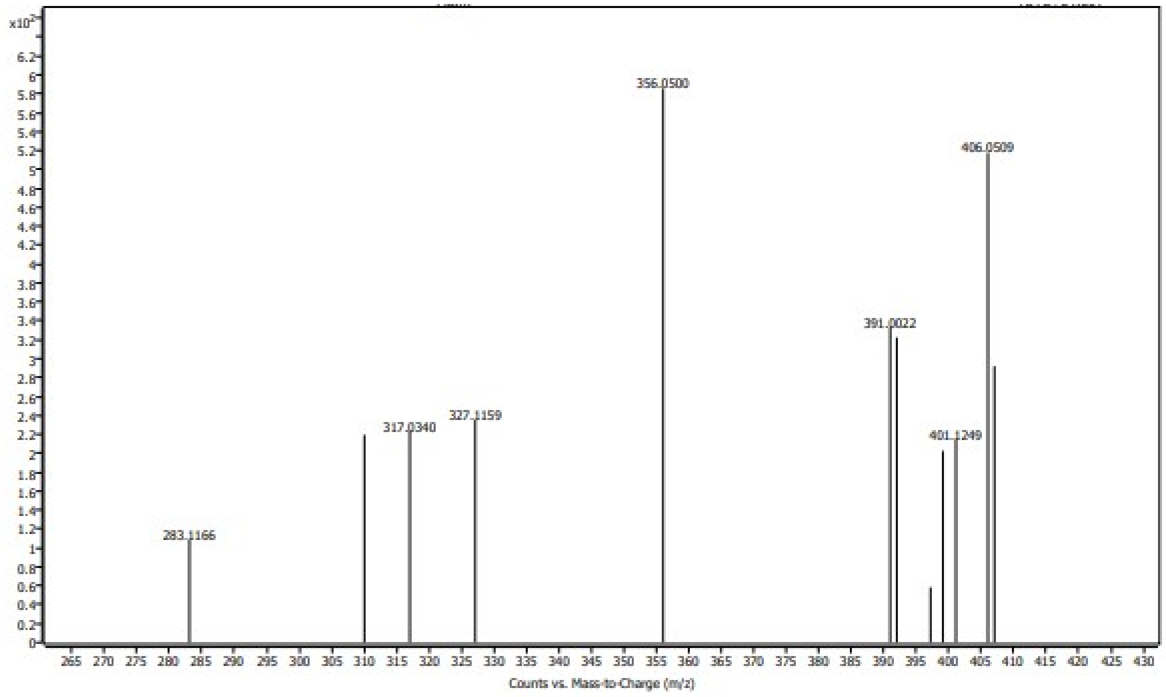

x10^2
356.0500
406.0509
391.0022
327.1159
317.0340
401.1249
283.1166
Counts vs. Mass-to-Charge (m/z)

^{1}HNMR, ^{13}CNMR, and mass spectra of compound 24

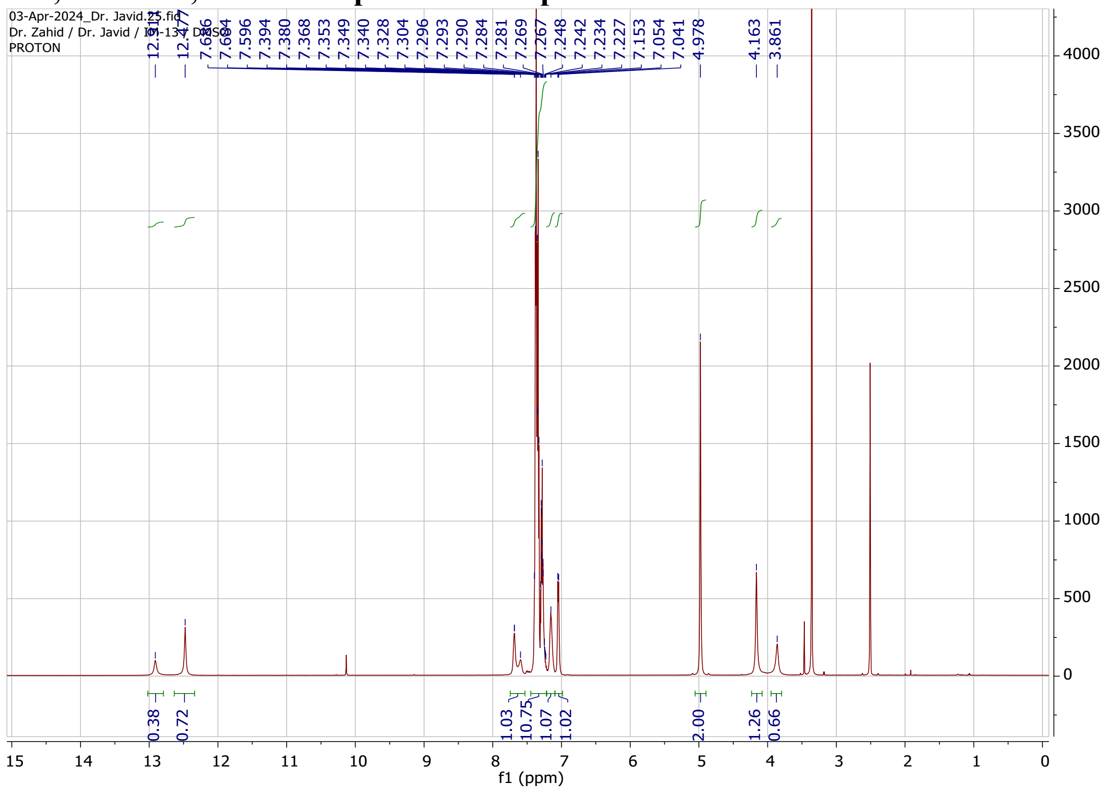

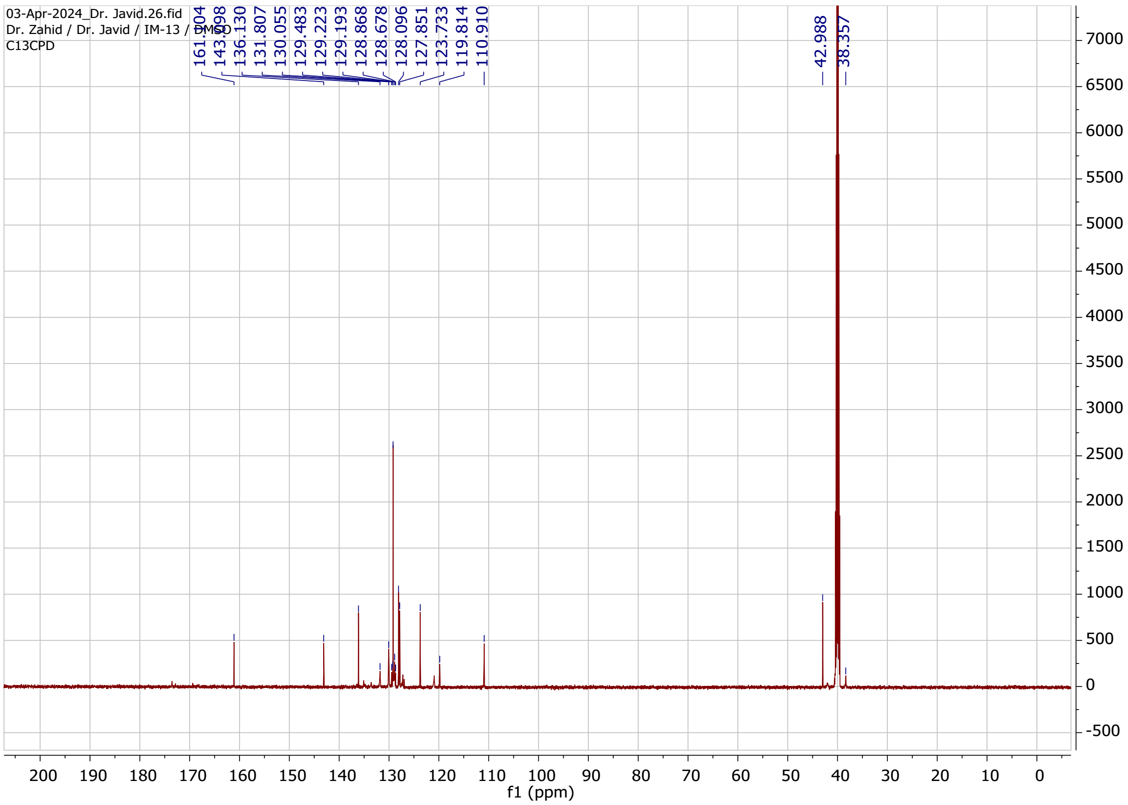

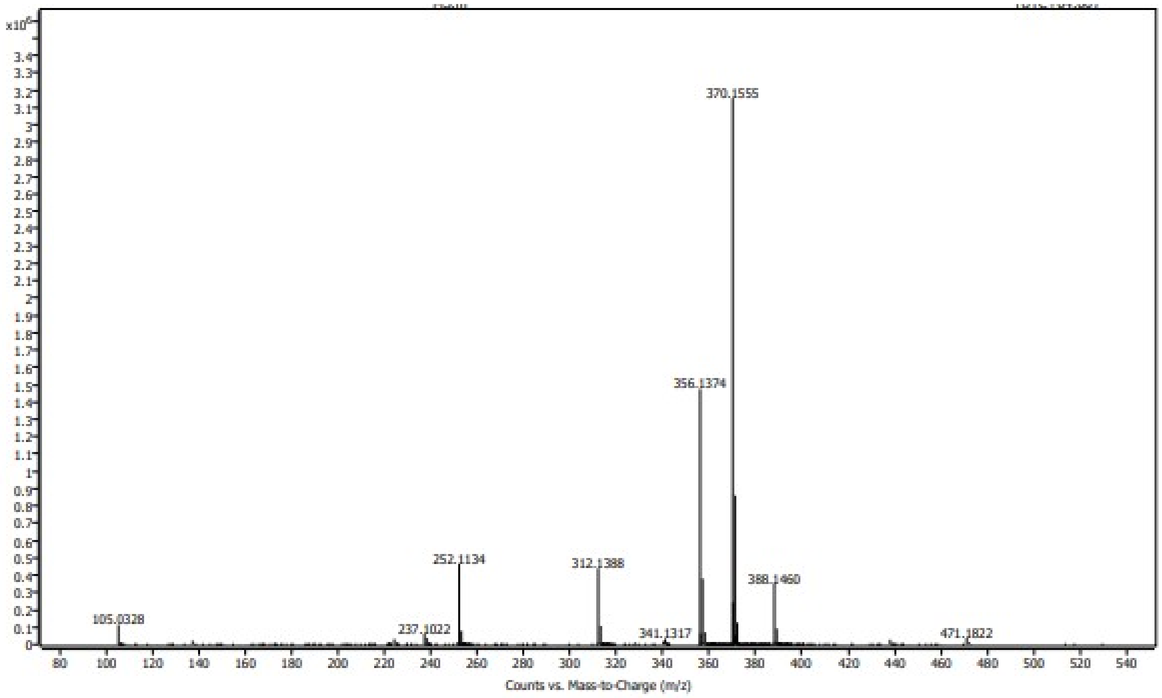

x10^6
370.1555
356.1374
252.1134
312.1388
388.1460
105.0328
237.1022
341.1317
471.1822
Counts vs. Mass-to-Charge (m/z)

^{1}HNMR, ^{13}CNMR, and mass spectra of compound 25

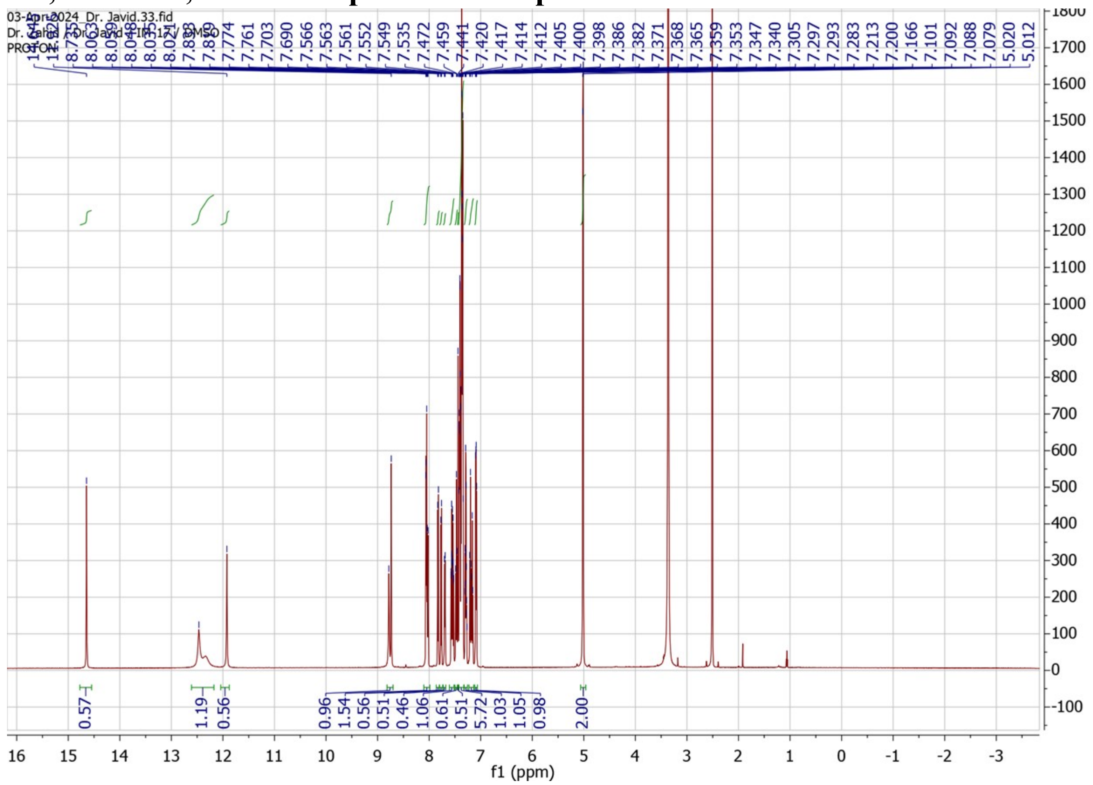

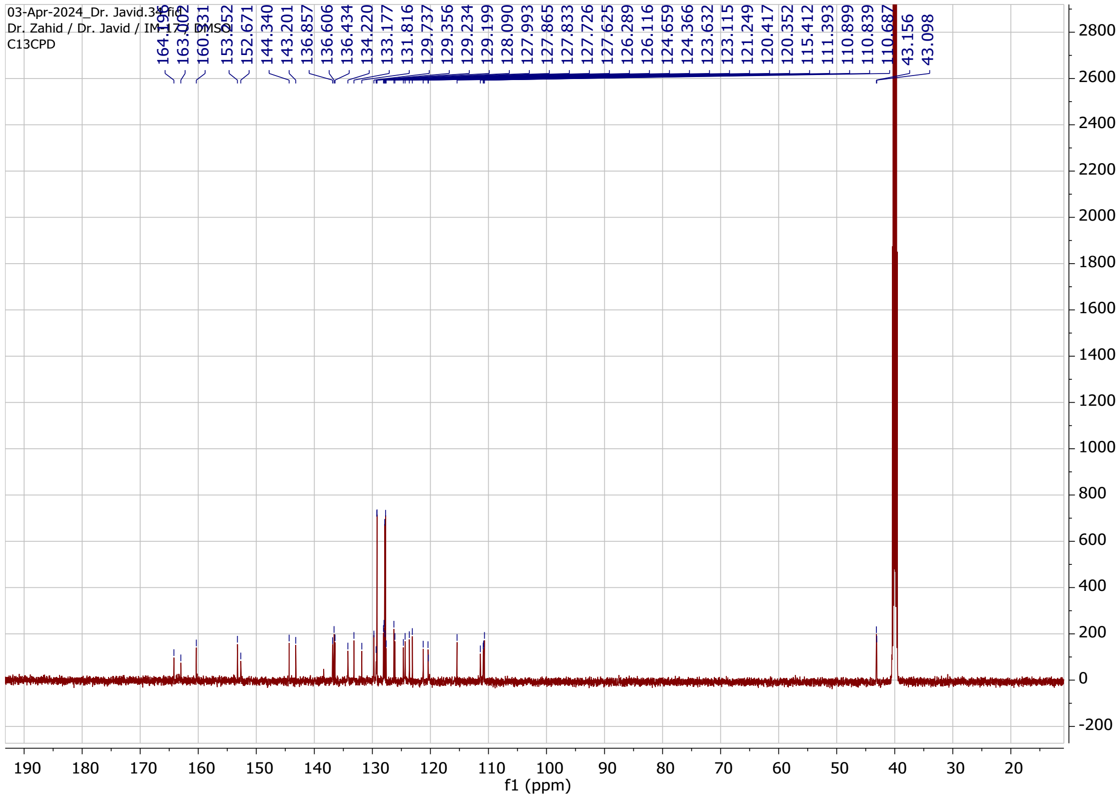

Sample Name	IM-17	Rack Position		Instrument	Instrument 1	Acq Operator	
Inj Vol (ul)	3	Plate Position		IRM Status	All ions missed		
Data File	IM-17.d	Acq Method	APCI POS ION DMSO MS.m	Comment		Acq Time (Local)	23-Sep-24 2:08:36 PM (UTC+04:00)

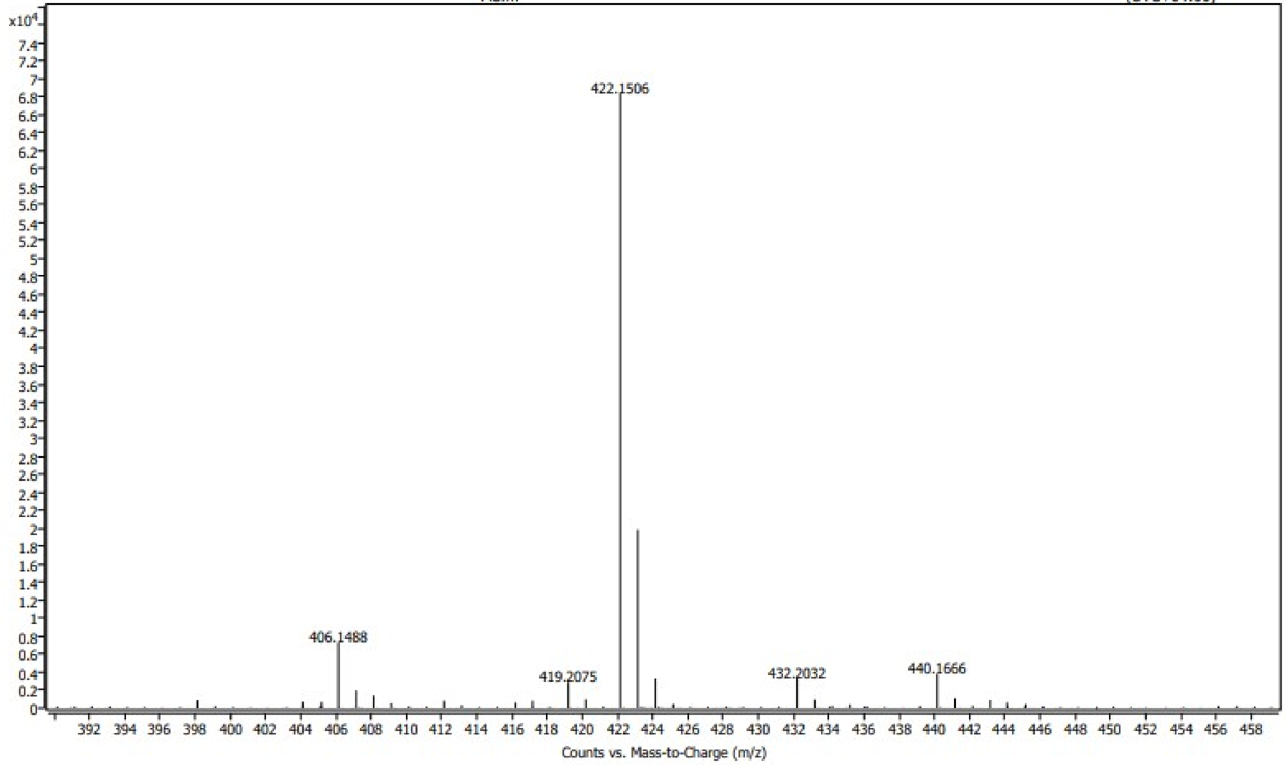

Supplement: RA-015-D4RA07650H-s001 [file RA-015-D4RA07650H-s001.pdf]
